# A charge transfer framework that describes supramolecular interactions governing structure and properties of 2D perovskites

**DOI:** 10.1038/s41467-022-31567-y

**Published:** 2022-07-08

**Authors:** Xiaoming Zhao, Melissa L. Ball, Arvin Kakekhani, Tianran Liu, Andrew M. Rappe, Yueh-Lin Loo

**Affiliations:** 1 Department of Chemical and Biological Engineering, Princeton University, Princeton, NJ 08544 USA; 2 Andlinger Center for Energy and the Environment, Princeton University, Princeton, NJ 08544 USA; 3 Department of Chemistry, University of Pennsylvania, Philadelphia, PA 19104-6323 USA; 4 Department of Electrical Engineering, Princeton University, Princeton, NJ 08544 USA

**Keywords:** Electronic materials, Solar cells

## Abstract

The elucidation of structure-to-function relationships for two-dimensional (2D) hybrid perovskites remains a primary challenge for engineering efficient perovskite-based devices. By combining insights from theory and experiment, we describe the introduction of bifunctional ligands that are capable of making strong hydrogen bonds within the organic bilayer. We find that stronger intermolecular interactions draw charge away from the perovskite layers, and we have formulated a simple and intuitive computational descriptor, the charge separation descriptor (CSD), that accurately describes the relationship between the Pb-I-Pb angle, band gap, and in-plane charge transport with the strength of these interactions. A higher CSD value correlates to less distortion of the Pb-I-Pb angle, a reduced band gap, and higher in-plane mobility of the perovskite. These improved material properties result in improved device characteristics of the resulting solar cells.

## Abstract (web summary)

Understanding structure-property relationships is important when designing functional materials. Here, authors propose a descriptor to help understand and predict the electronic properties of two-dimensional lead iodide perovskites for photovoltaic applications.

## Introduction

Hybrid organic-inorganic perovskite solar cells efficiently absorb light and generate electricity, with power-conversion efficiencies now exceeding 25%^1–7^. However, these solar cells are unstable because halide perovskites degrade when exposed to light, heat, or moisture^8–11^. Two-dimensional perovskites have been investigated as both a capping layer for three-dimensional hybrid inorganic-inorganic solar cells and as a complementary solar technology in recent years^12,13^. These lower-dimensional systems have the formula A′_*m*_A_*n*−1_B_*n*_X_3*n*+1_. A′ represents a plane of monoammonium or diammonium organic cations that separate one set of perovskite layers from the next, and *n* can be understood as the number of consecutive perovskite layers between the A′ organic layers. When a monofunctional cation (e.g., BA^+^) is used, the A′ plane needs two molecules per formula unit (*m* = 2), and these 2D perovskites are normally referred to as Ruddlesden-Popper (RP) phase 2D perovskites. 2D perovskites formed with bifunctional cations (e.g., BDA^2+^) generally need one per formula unit (*m* = 1), have the perovskite layers vertically aligned, and are referred to as Dion-Jacobson (DJ)-phase 2D perovskites. Two-dimensional perovskites with organic ammonium spacer ligands offer improved stability, but the power-conversion efficiencies of these solar cells are low, reflecting inefficient charge transport^14–19^.

The electronic and charge transport properties of 2D perovskites are closely related to the organization of their inorganic frameworks^20,21^. In particular, the intermolecular interactions between the organic cations and inorganic octahedra have a large effect on the degree of octahedral tilting, and, as a result, on the optoelectronic properties and device performance^20–26^. Thus, there is a pressing need for molecular design strategies that can manipulate the organic-inorganic interactions to affect the degree of octahedral tilting. Bifunctional ligands present an interesting class of molecules that can be used to manipulate the degree of octahedral tilting and Pb-X-Pb angles. These ligands contain one ammonium terminus, while the non-ammonium terminus contains a moiety capable of making strong lateral supramolecular interactions within the organic bilayer. The organic ligands and inorganic octahedra are molecularly stitched via charge transfer and hydrogen-bonding interactions between proton-like hydrogens of the ammonium terminus and the halide ions of the inorganic octahedra^27^. Such interactions are usually thought of as hydrogen bonds. Recent literature, however, shows that these interactions exhibit a stronger electrostatic component than conventional hydrogen bonds, given the cationic nature of the ammonium terminus^27^. The role of additional chemical functionality on the non-ammonium terminus has not been extensively explored, yet this class of ligands is interesting as the introduction of additional chemical moieties creates further opportunities for lateral supramolecular interactions^18–21^. Earlier works by Batail^24^ and Mercier^24–26^ discussed the perturbation in the Pb-X-Pb angle with the introduction of a halogen or hydrogen bonding functionality on the non-ammonium terminus. Ren et al. also recently employed a bifunctional strategy with the introduction of a thiomethyl-terminated organic ligand, which resulted in improved materials properties of 2D perovskites and performance of devices containing them^28,29^.

Inspired by these studies, we hypothesize that additional opportunities for lateral supramolecular interactions on the non-ammonium terminus can result in intra-plane couplings that influence the amount of octahedral tilting in the inorganic plane. Here, we synthesize, characterize, and model two-dimensional perovskites that contain bifunctional organic ligands; we investigate the molecular interactions that govern structural parameters, we elucidate how these supramolecular interactions induce favorable structural conformations, and we devise a descriptor that quantifies these interactions. We show how these interactions directly lead to chemical, electronic, and optoelectronic modifications that improve photovoltaic performance. This descriptor-based materials design paradigm can guide future innovations in solution-processable perovskite optoelectronics; case in point is our demonstration of (CN–EA)_2_(MA)_3_Pb_4_I_13_ solar cells with a power-conversion efficiency of 16.4% that are operationally stable >1000 h^30–32^.

## Results

**Fig. 1:**
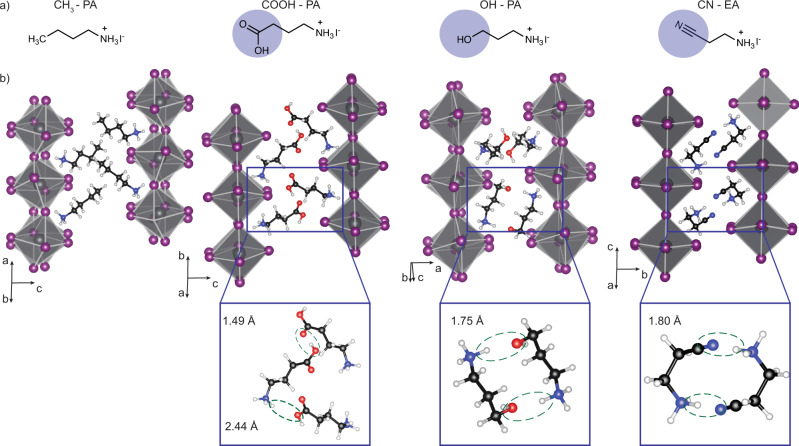
Interactions between bifunctional organic ligands and inorganic octahedra. **a** Organic ligands used in this study. The purple circles highlight the non-ammonium terminal functional groups; **b** the theoretical 2D perovskite structure for (CH_3_–PA)_2_PbI_4_, (COOH–PA)_2_PbI_4_, (OH–PA)_2_PbI_4_, and (CN– EA)_2_PbI_4_. The insets show hydrogen-bonding interactions between adjacent ligands across the organic bilayer. Green dashed ovals represent close contacts between the RH_2_N^+^–H…R‘, where R‘ = −OH in (COOH -  PA)_2_PbI_4_, −OH in (OH–PA)_2_PbI_4_, and −CN for (CN–EA)_2_PbI_4_.

We have investigated the role of three bifunctional ligands, each with a non-ammonium terminus capable of making hydrogen bonds within the organic bilayer (highlighted in purple) through a −CN, −OH, or −COOH moiety. We show the chemical structures of these ligands in Fig. 1a, b contains the crystal structures for the resulting *n* = 1 perovskites of (CH_3_–PA)_2_PbI_4_, (COOH–PA)_2_PbI_4_, (OH–PA)_2_PbI_4_, and (CN–EA)_2_PbI_4_ (PA = propylammonium, EA = ethyl ammonium). The Supplementary Information contains further structural information and a comparison of the experimental and theoretical structures (Supplementary Figs. 1–3 and Supplementary Table 1). The experimental and theoretical Pb-I-Pb angles and interlayer spacings are quantitatively similar, suggesting that theory has adequately captured the experimental structures of these perovskites. The ligands investigated have lengths of ~4 Å (CN–EA and OH–PA), 5 Å (CH_3_–PA), and 6 Å (COOH–PA) (Supplementary Table 2).

The key to tuning the macroscopic optoelectronic properties of 2D perovskites is the interactions between adjacent organic ligands within the organic bilayer in each materials system. Both (OH–PA)_2_PbI_4_ and (CN–EA)_2_PbI_4_ form dimer species across the organic bilayer. Structural analysis indicates that the pseudo-hexagonal shape of the (CN–EA)^+^ dimer is formed through a pair-wise hydrogen bond between the ammonium terminus of one ligand and the cyano group of the adjacent ligand across the organic bilayer, while the pseudo-rectangular shape of the (OH–PA)^+^ ligand reflects a pair-wise interaction between the ammonium terminus and the hydroxyl moiety of adjacent ligands. The (COOH–PA)^+^ ligands assemble into a “molecular zipper” rather than a dimer, reflecting their relative bulky size (Fig. 1b). While hydrogen-bonding interactions have been observed previously in these structures^24,26^, the goal of this study is to quantify the molecular interactions that govern structural parameters and relate the structural distortions to the macroscopic properties of the resulting 2D perovskites and the performance of solar cells incorporating these materials as active layers. To highlight the impact of the non-ammonium termini making point interactions within the organic bilayer, we also studied (CH_3_ –PA)_2_PbI_4_ as a control sample, as the CH_3_–PA^+^ ligand does not exhibit strong intermolecular interactions within the organic bilayer other than ubiquitous long-range dispersion-type interactions.

To better understand the underlying chemical interactions involved within the organic bilayer, we detail here our analysis of dimer formation in (CN –EA)_2_PbI_4_. Such analyses for (OH–PA)_2_PbI_4_, (COOH–PA)_2_PbI_4_, and (CH_3_–PA)_2_PbI_4_ are provided in Supplementary Note 1 and highlighted in Supplementary Figs. 4 and 5. The COOH ligand has previously been used as an additive to perovskite precursor solutions to yield three-dimensional perovskites that result in solar cells with enhanced performance and improved stability^33,34^, and also within structural studies for two-dimensional perovskites^35^.

**Fig. 2:**
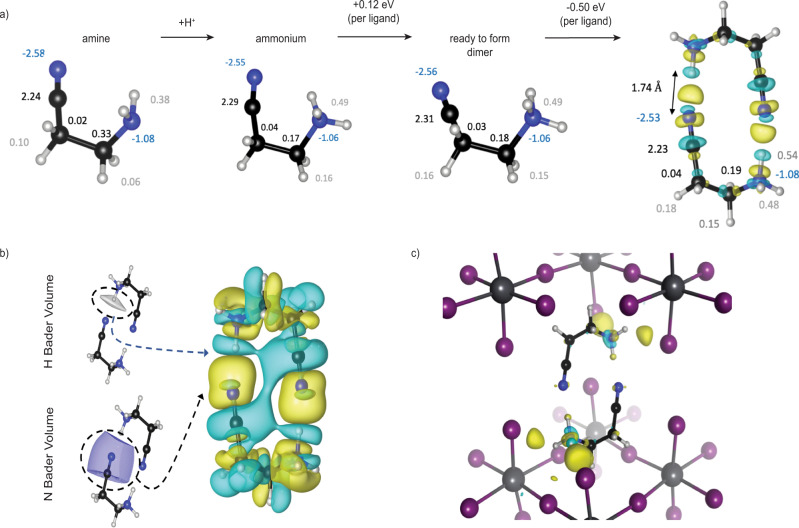
Organic interactions, and their influence on the inorganic backbone. **a** Protonation of an amine to form ammonium, mechanical deformation to create a dimer-ready ligand, and dimer formation. Numbers indicate atomic Bader charges. The electron-density difference (EDD) plot show changes in electron density upon dimer formation from two monomers. The rendered yellow and cyan regions correspond to electron accumulation and depletion. Color code: nitrogen (blue), carbon (black), hydrogen (white), lead (gray), and iodine (purple); **b** electron accumulation (bond) between two ligands, associated with a cyclic dipole formation around the ring, shifting electrons to electrophilic regions close to the proton-like hydrogens. EDD isosurfaces correspond to smaller charge transfers, relative to (**a**, **c**), to better portray dipole formation. Bader volumes indicate charge sharing (covalency) and a small charge transfer from the nitrogen on one ligand to the hydrogen on the other; **c** formation of strong hydrogen bonds between the dimer and the axial iodides of the inorganic backbone. We observe no strong interactions between the ammonium group and an equatorial iodide.

Point interactions between two neighboring organic ligands cause the ammonium group to reorient and the ligands to adopt non-ideal geometries (relative to the ground state geometry not embedded in a perovskite structure). For example, the curved “U” shape conformation of the five-atom chain of (CN–EA^+^) results in a tightly bound dimer with a pseudo-hexagonal geometry formed between the nitrogen lone pair of the cyano group of one ligand and the proton-like hydrogen on the adjacent ligand’s ammonium group (Figs. 1b and 2a). We find that dimer formation between two (CN–EA^+^) cationic monomers is aided by and is only possible due to the countercharge of the inorganic background; otherwise, the two cations do not favor the formation of a tightly bound dimer (Supplementary Figs. 6 and 7). We find that while the dimer-ready conformation costs ~0.12 eV in energy (relative to a relaxed ligand), there is an ~0.50 eV (per ligand) energy payback upon dimer formation (Fig. 2a) in the perovskite environment. Such computational information is provided by numerical experiments performed using plane-waves-based density functional theory (DFT) with add-on corrections for dispersion-type interactions. Using carefully designed computational setups, we applied this method to study both periodic systems (e.g., the perovskite structure) and isolated dimers investigated in our Gedanken experiments. Details on the theoretical methods, including the energetics involved in dimer formation, are found in the Methods section and Supplementary Note 1.

CN–EA experiences two types of interactions in the hybrid perovskite structure: point interactions within the (CN–EA)_2_ dimer and interactions with the inorganic backbone (PbI_6_ octahedra). Focusing first on the former, and employing DFT calculations to study the cationic organic ligands, we find that dimer bonding is associated with the electron cloud shifting from the lone pair on the cyano group (Lewis base) toward the positive center at the proton-like hydrogen on the ammonium cation (strong Lewis acid site) (Fig. 2b)^27,36,37^. This electron redistribution also leads to the formation of consecutive induced dipoles around the pseudo-hexagonal ring (Fig. 2b), represented by alternating charge accumulation and depletion regions around the ring. The collective effect of such induced dipoles is to place more electron density in the electrostatic potential well created by the proton-like hydrogen nearest to the cyano group. In the process, the proton-like hydrogen loses some electron density (and the associated ammonium N–H bond elongates) to maximize its attractive potential well and minimize electron-electron repulsion with the incoming cyano lone-pair (Supplementary Table 3)^27^. The interaction between the cyano group of one ligand and the proton-like hydrogen of the adjacent ligand also shows some ionic-covalent character; low-energy bonding molecular orbitals form between the two molecules and some charge transfers from the cyano group of one molecule to the proton-like hydrogen of the other (See Supplementary Note 1 and Supplementary Figs. 8–10).

The interactions between the organic ligand and inorganic backbone (PbI_6_ octahedra) are also important to understand. In order to assemble into a dimer, the (CN–EA)^+^ must withdraw the ammonium termini away from the inorganic sheet, achieved by tilting the alkylammonium group, effectively eliminating the ammonium’s interaction with the equatorial iodides and enhancing the hydrogen bonding between the ammonium terminus and the axial inorganic halides (Fig. 2c). The electron density difference (EDD) plot in Fig. 2c reveals such interactions between the dimer and the inorganic octahedral backbone. There are also non-covalent dispersion interactions with the inorganic cage (Supplementary Fig. 11). The structural changes to the dimer due to embedding in the perovskite structure are shown in Supplementary Fig. 12.

In analyzing the structures of the other 2D perovskites in this study, we find the point interactions between adjacent organic ligands to directly affect the ligands’ interactions with the inorganic backbone, and, thus, the assembly of the resulting perovskite. As the Pb-I-Pb angle and interlayer spacing strongly correlate with macroscopic materials properties, this link connects the interactions within the organic bilayer to the functional properties of our 2D perovskite-based devices.

### Charge separation descriptor

**Fig. 3:**
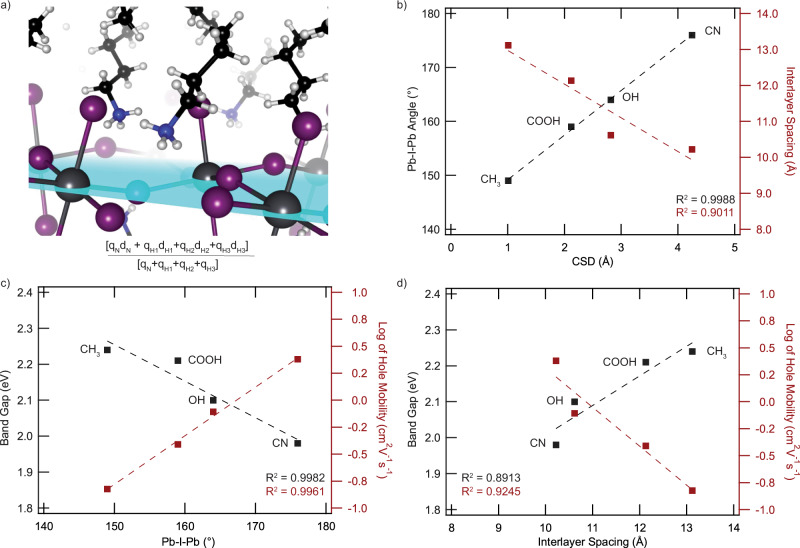
Charge separation descriptor (CSD) plots to show the relationship between CSD and structural properties and then structural properties to materials properties. **a** A schematic showing how the CSD value is calculated. index *i* indicates atoms in the ammonium groups, *q*_*i*_ is the Bader charge of an atom, an*d d*_*i*_ is its distance to the middle of the inorganic plane; **b** a close linear correlation is seen between descriptor CSD and both the average Pb-I-Pb angle and the interlayer spacing from experimental structural files; **c** correlation between the Pb-I-Pb angle (from experimental structural files) and log of hole mobility and band gap; and **d** correlation between interlayer spacing and log of hole mobility and band gap. Band gap was determined by UPS to determine the valence band maxima and subtracted this from the E_g_ from absorption of single crystals to determine the valence band minima. The hole mobility was determined by space charge limited current measurements. See the “Methods” section for further details.

To formulate how interactions in the organic bilayer can control the structural and electronic properties of 2D perovskites, we developed a simple and intuitive descriptor. The charge-separation descriptor, CSD, is defined as the separation between the center of (Bader) charge of the ^+^NH_3_ group and the middle of the inorganic plane (Fig. 2d):

1 $${{{{{\rm{CSD}}}}}}=\frac{{\sum }_{{{{{{\rm{i}}}}}}}{q}_{{{{{{\rm{i}}}}}}{d}_{{{{{{\rm{i}}}}}}}}}{{\sum }_{{{{{{\rm{i}}}}}}}{q}_{{{{{{\rm{i}}}}}}}}$$

where the index *i* indicates atoms in the ammonium groups, *q*_*i*_ is the Bader charge of an atom, and *d*_*i*_ is its distance to the middle of the inorganic plane (Fig. 3a). The CSD takes into account the ammonium nitrogen as well as its proton-like hydrogens. The “Methods” section contains further details on the calculation of the CSD.

Favorable interactions within the organic bilayer reorient the ammonium group and can cause the ligand to adopt a non-ideal geometry relative to the ground state geometry of a non-embedded ligand. The reorientation of the ammonium group moves the center of charge on the ammonium terminus away from the inorganic sheet toward the center of the organic bilayer, resulting in a larger CSD value. Across the four perovskites explored, the CSD directly correlates with the Pb-I-Pb angle, with *R*^2^ =  0.9988 (Fig. 3b). The largest CSD of the perovskites in this series, (CN– EA)_2_PbI_4_, has a Pb-I-Pb angle that is close to 180°, while (CH_3_– PA)_2_PbI_4_, with the smallest CSD, possesses a Pb-I-Pb angle of 145° (Fig. 3b). When deconvoluting the Pb-I-Pb into its in-plane and out-of-plane components, we find the correlation to be greater between CSD and the in-plane Pb-I-Pb distortion; this observation is analogous to others^20^, and is the subject of further studies (Supplementary Fig. 13). We also investigated the correlation between interlayer spacing and CSD, and find there to also be a strong correlation with an *R*^2^ = 0.9011 (Fig. 3b). As the interlayer spacing decreases, the CSD value monotonically decreases across the four perovskite structures.

Further, we were curious to understand the predicting power of the CSD model using a more diverse set of ligands. Supplementary Fig. 14 includes data for the four perovskites studied here, and that of commonly studied perovskite comprising phenethylamine (PEA), a relatively large aryl ligand, and butyldiammonium (BDA), a ligand that forms a Dion-Jacobson phase. We also include perovskite containing naphthalene-O-propylamine (NAPH), an ether-appended naphthalene ligand, to evaluate the robustness of our framework since a previous model that measured the nitrogen-to-axial iodide distance failed to correlate its structure with materials properties^38,39^. We find that the CSD model maintains its ability to correlate to the Pb-I-Pb angle as demonstrated with a *R*^2^ = 0.9634. Although the correlation between CSD and interlayer spacing for perovskites with alkyl ligands is strong, this correlation is weaker across a more diverse set of ligands, as shown in Supplementary Fig. 14. This weaker correlation suggests that factors—beyond the molecular interactions captured by CSD—dominate the interlayer spacing when one considers a broader set of ligands. Since ligand size and geometry also influence interlayer spacing, this weaker correlation between CSD with interlayer spacing across a wider range of chemistries may not be surprising retrospectively; future studies will assess whether this correlation holds more strongly within perovskites having specific classes of ligands (Supplementary Fig. 14). The lack of correlation in interlayer spacing also points to the importance of the Pb-I-Pb angle as a predictor of materials properties in two-dimensional perovskites. Supplementary Fig. 15 plots other distance-based descriptors; including the nitrogen to iodide(axial) distance, relative to both the Pb-I-Pb angle and band gap, which provides a benchmark against how well CSD captures point interactions in impacting perovskite assembly across a diverse set of ligands relative to other descriptors. We find that CSD shows the strongest correlations of all descriptors examined herein (Supplementary Fig. 15 and Supplementary Tables 3 and 4). Future work will assess how broadly the CSD framework can be applied to a larger set of ligands in an effort to identify any predicting limitations of the descriptor.

In order to probe whether molecular fluctuations have an impact on the correlations between CSD and structure, we also plotted CSD against the theoretically determined Pb-I-Pb angle and band gap using the calculated structural files. The deviation between the experimentally- and theoretically determined Pb-I-Pb angles and interlayer spacings are each <4%. Thus the correlations using the theoretical values are consistent with those obtained using the experimental data, and we interpret this observation to indicate that either the thermal fluctuations and dynamics are small and do not affect the estimation of structure and macroscopic properties (vida infra), or that the thermally averaged structures can capture the ensemble average structure and materials properties. Thus, our observations suggest that engineering the extent of interactions in the organic bilayer and the consequent influence on the inorganic backbone in 2D perovskites renders the CSD tunable and yields control over the octahedral distortions.

### Optoelectronic properties and charge transport

Both structural properties Pb-I-Pb angle and interlayer spacing show strong correlations with materials properties (bang gap and hole mobility) across the four perovskites studied (Fig. 3c, d, Supplementary Figs. 16–25, Supplementary Tables 4 and 5). We observe linear relationships between the Pb-I-Pb angle and both the band gap (*R*^2^ = 0.9982) and the log of the hole mobility (*R*^2^ = 0.9961). Having the highest CSD, (CN –EA)_2_PbI_4_ has the smallest band gap; with the lowest CSD, (CH_3_–PA)_2_PbI_4_ exhibits the widest band gap. A smaller deviation from the ideal Pb-I-Pb angle = 180° should also increase charge transport within the inorganic plane by providing better orbital overlap^20,21,40,41^. As such, we investigated hole mobilities of the *n* = 1 2D perovskite single crystals using space-charge limited current (SCLC) method. At 2.4 ± 0.4 cm^2^ V^−1^ s^−1^, (CN–EA)_2_PbI_4_ exhibits the highest in-plane hole mobility of the perovskites in this series, while (CH_3_–PA)_2_PbI_4_ has the lowest mobility of 0.5 ± 0.04 cm^2^ V^−1^ s^−1^ (Supplementary Tables 6 and 7). Interlayer spacing also shows a strong linear dependence with both the band gap and the log of the in-plane hole mobility, although the rationale for this relationship is less intuitive, given the insulating nature of the organic ligands and the location of the frontier orbitals within the inorganic plane. Case-in-point, the out-of-plane mobility is substantially lower and invariant across for both the *n* = 1 (single crystals) and *n* = 4 (thin films) perovskite structures with different interlayer spacings (Supplementary Fig. 26).

We fabricated thin films of the *n* = 4 perovskites with high phase purity^42^ to measure both hole mobility and band gap, and find the trend in materials properties for the *n* = 4 perovskites is in good agreement with that of the *n* = 1 data, as shown in Supplementary Table 7^42^. For example, the largest CSD *n* = 1 perovskite, (CN–EA)_2_PbI_4_, shows both the smallest band gap (1.90 eV) and highest in-plane hole mobility (2.4 ± 0.4 cm^2^ V^−1^ s^−1^) among our four *n* = 1 2D perovskites, while for our four *n* = 4 2D perovskites, CN–EA based 2D perovskites also show the smallest band gap (1.64 eV) and highest in-plane mobility (7.8 ± 1.2  cm^2^ V^−1^ s^−1^) among them. Although we were unable to obtain single-crystal structures for the *n* = 4 2D perovskites, the strong correlation in materials properties between the *n* = 1 and *n* = 4 perovskites suggests the trend in the Pb-I-Pb angles are also in good agreement between the two perovskite assemblies, an analogous finding to previously reported results that show *n* = 4 structures follow the same trend in the Pb-I-Pb angle and interlayer spacing as their *n* = 1 analogs^43^.

### Performance in solar cells

We fabricated *n* = 1 2D perovskite solar cells to demonstrate how altering the properties of 2D perovskites impact solar cell performance and find that solar cell performance correlates with CSD. (CN–EA)_2_PbI_4_ solar cells have a champion power conversion efficiency of 3.13%, which is triple that of (CH_3_–PA)_2_PbI_4_ solar cells (Supplementary Figs. 27 and 28 and Supplementary Table 8). The difference in solar cell performance reflects the more extended light absorption in the near-infrared region given lower bandgaps and higher carrier mobilities of 2D perovskite systems with higher CSD compared to lower-CSD systems. We find it interesting that the performance of solar cells comprising these materials, which can be influenced by a myriad of extrinsic factors, correlates with CSD, providing a handle on the molecular interactions that drive perovskite assembly (Supplementary Fig. 28).

**Fig. 4:**
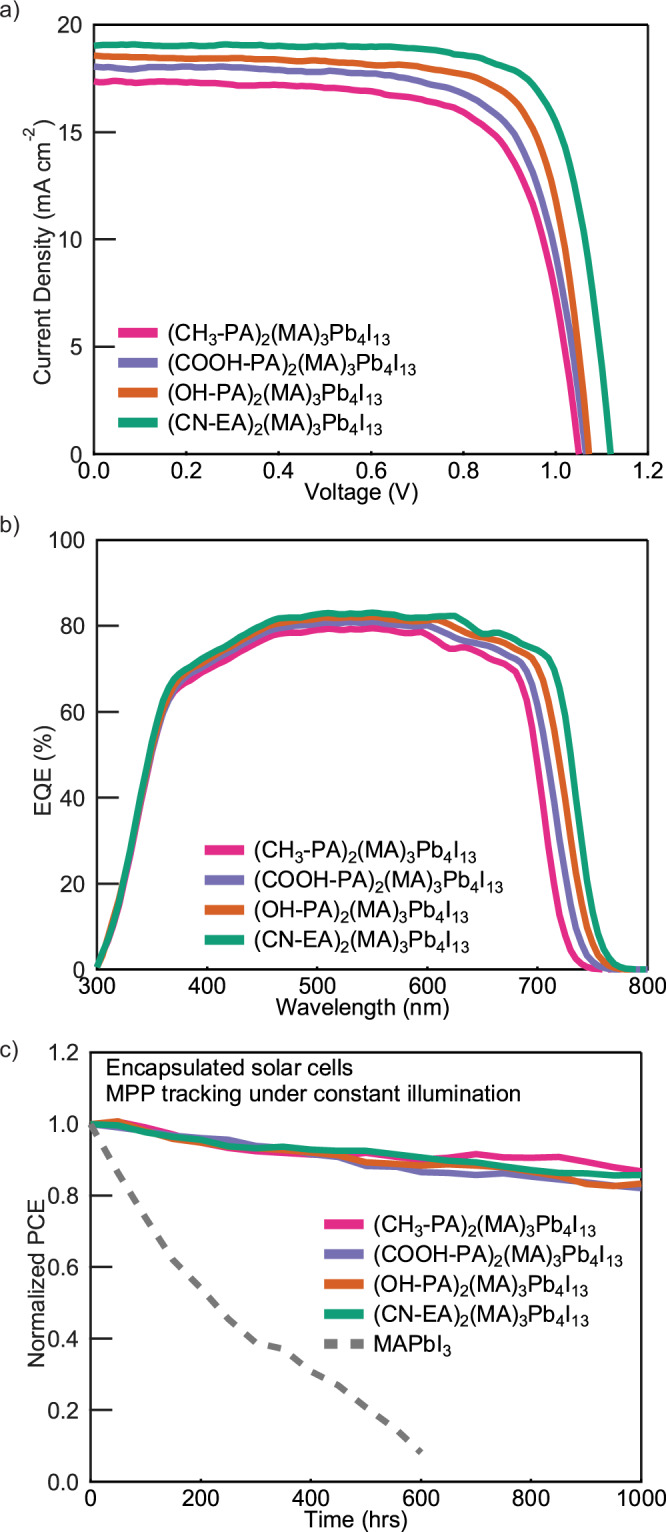
Device characterization for *n* = 4 perovskite devices. **a** *J-V* characteristics under reverse scan; **b** External quantum efficiency (EQE) of *n* = 4 2D perovskite solar cells; and **c** normalized PCE as a function of operation time of encapsulated 2D perovskite solar cells under continuous illumination at maximum power point (MPP) tracking.

We also fabricated and characterized solar cells with *n* = 4 2D perovskites films using the ligands studied above, as higher n systems are the subject of intense interest as their performance metrics are approaching values applicable for real-world applications. Figure 4a shows the J-V curves collected under simulated AM 1.5 G one-sun illumination. The lowest CSD photoactive layer (CH_3_–PA)_2_(MA)_3_Pb_4_I_13_ resulted in solar cells with the lowest PCE of 13.1%, while solar cells based on films quantified by higher CSD values show substantively improved PCEs. Among them, the (CN–EA)_2_(MA)_3_Pb_4_I_13_ solar cell exhibits a champion PCE of 16.4%. Similar to the solar cells containing *n* = 1 perovskites, the improvement in PCE is consistent with a narrowing of band gap and increased hole mobility (Fig. 4b). Therefore, we believe that the organic ligand and associated interactions in the organic bilayer play a meaningful role in the improved device characteristics across the series. Further details, e.g., film orientation, and performance metrics for all devices studied in solar cells for *n* = 4 devices can be found in Supplementary Figs. 29–37.

We find that all perovskites solar cell devices show superior operational stability than their 3D counterparts—MAPbI3 solar cells, with the time when solar cell PCE reaches 80% of its initial PCE (T_80_) >1000 h; we found no significant difference in the operational stability of solar cells containing the four different perovskite chemistries (Fig. 4c). This observation suggests that the introduction of organic bilayer interactions may not significantly affect perovskite stability under operating conditions. We also measured fresh unencapsulated devices as well as those aged for 6 months in the dark at 25 °C in 40–60% R.H. air comprising *n* = 4 2D perovskites, and find that all four devices show negligible environmental degradation—retaining over 96% of their initial PCE—while the unencapsulated MAPbI_3_ 3D perovskite solar cell shows a marked deterioration in performance from an initial PCE of 18.9% to 2.8% when aged under the same conditions (Supplementary Fig. 38).

### Remarks on design rules for organic ligands

Above, we showed that by tuning the CSD one can improve the efficiency of 2D hybrid perovskite solar cells. In order to render the process of screening systems for optimum optoelectronic properties more rational, we put forth the following observations: (1) Increased saturation around the non-ammonium terminus results in a larger deviation from the ideal 180° octahedral angle. In (OH–PA)^+^, each carbon atom in the aliphatic chain is saturated (bonded to two hydrogen atoms), providing maximal flexibility to both termini. Thus, the ammonium terminus does not have to withdraw as extensively from the interstitial site because the flexibility in the –C–C–C–O– chain can accommodate the (elongated pseudo-rectangular) dimer formation. We conjecture that the unsaturated nature of the cyano terminus, and the resulting rigidity required from an alkyne geometry, promotes the formation of a U-shaped dimer, as the relatively less rigid ammonium terminus must withdraw from the interstitial site and rotate to create favorable point interactions with the (axial) halides on the inorganic octahedra. (2) The larger size of (COOH–PA)^+^ relative to the other organic ligands in this study suggests that dimer formation requires less sterically demanding functionalities. (3) The choice of heteroatom appears to also impact the strength of point interactions in the organic bilayer. The greater negative Bader charge on the nitrogen atom of the cyano group on CN–EA^+^ relative to that of the oxygen on the hydroxyl group of OH–PA^+^ leads to stronger electrostatic point interactions via a more tightly bound dimer, resulting in a less distorted inorganic layer.

In this work, we show a critical link between tunable supramolecular interactions in 2D hybrid perovskites and high-performing solar devices. We show that equipping the non-ammonium terminus of the spacer organic ligands with a chemical moiety that can interact with the ammonium group’s proton-like hydrogens on the adjacent ligand across the organic bilayer is a type of bifunctionality that can be used to tune the optoelectronic properties of the hybrid perovskite system. We believe the CSD differs from others in the literature as it lends insight into the molecular interactions that govern structural parameters. Our theoretical framework offers insights to predicting the octahedral tilt, which to first order, dictates perovskite optoelectronic properties. The CSD correlates well with experimental quantities, including band gap and charge carrier mobilities. It thus provides structure-to-function relationships that can be used to optimize the performance of perovskite optoelectronic devices. Future studies will assess whether the CSD framework has predictive power across (1) a more diverse ligand set, other than small alkyl ammonium ligands; (2) in perovskites with different compositions such as substituting other halogens in place of iodides and other metal centers such as tin; and (3) in higher-order perovskite systems such as *n* > 1 assembly. We believe the solar cell community will be able to leverage our proposed design rules to guide future exploration of various types of bifunctional organic ligands or even point interactions beyond hydrogen bonding to advance materials design in 2D perovskites.

## Methods

### Materials

PbI_2_ (99.99%) was purchased from TCI America. MAI (>99%), BAI (> 99%), and TiO_2_ paste (30 NR-D) were purchased from Greatcell Solar. DMF (>99.8%), ACN (>99.8%), THF (>99.8%), and IPA (>99.8%) were purchased from Acros Organics. Acetylacetone (>99%), titanium diisopropoxide (75 wt. % in isopropanol), Spiro-OMeTAD (>99%), Li-TFSI (99.95%), t-BP (98%) were purchased from Sigma Aldrich. Acetone, ethanol and other solvents were purchased from Fisher Scientific. All chemicals were used as received without further purification. Solar cell substrates are pre-patterned fluorine-doped tin-oxide-coated (FTO) glass (<15 Ω/square) obtained from Yingkou Advanced Election Technology Co., Ltd. The CIFs we employed for this study were downloaded from CCDC. Their reference numbers are:

(CH3-PA)_2_PbI_4_: CCDC 665689 [10.5517/ccqbpvk]

(COOH–PA)_2_PbI_4_: CCDC 267398 [10.5517/cc8z7r6],

(OH–PA)_2_PbI_4_: CCDC 746125 [10.5517/cct1dks],

(CN–EA)_2_PbI_4_: CCDC 705087 [10.5517/ccrnprt]

(PEA)_2_PbI_4_: CCDC 1841681 [10.5517/ccdc.csd.cc1ztf29],

(BDA)PbI_4_: CCDC 1053651 [10.5517/cc14cdrn]

(NAPH)_2_PbI_4_: CCDC 1840803 [10.5517/ccdc.csd.cc1zshr0].

### Synthesis and characterization

*n* = 1 2D perovskite single crystals were synthesized by a revised method to the reference (Nano Lett.2018, 18, 3221−3228). 5 mmol PbI_2_ were dissolved in 5 mL HI solution with 0.85 mL hypophosphorous acid and heated to 110 °C to get a clear yellow solution. In a separate vial, 10 mmol amine (i.e., BA, COOH–PA, OH–PA or CN–EA) was dissolved with 5 mL HI solution under ice bath and thereafter this solution was added dropwise into the hot PbI_2_ solution with continuous stirring. Once a clear yellow solution was formed, the stir bar was removed from the vial and the vial was put on a programmable hotplate and slowly cooled down from 110 to 40 °C at a rate 1 °C/h. The obtained single crystals were filtered and washed by copious amount of diethyl ether. Finally, the crystals were put in a vacuum oven at 40 °C overnight to remove residual solvents. COOH-PAI, OH-PAI, and CH_3_-PAI are known compounds^25,26^. CN-EAI is characterized in the Synthesis and Characterization section of the SI. Both proton nuclear magnetic resonance (^1^HNMR) spectra and carbon nuclear magnetic resonance (13CNMR) spectra were recorded on a Bruker 500 AVANCE equipped with a cryoprobe (500 and 125 MHz, respectively) at 25 °C. Chemical shifts for protons are reported in parts per million (ppm) downfield from tetramethylsilane and are referenced to residual protium in the NMR solvent (DMSO = 2.50 ppm). Chemical shifts for carbon are reported in parts per million downfield from tetramethylsilane and are referenced to the carbon resonance of the solvent peak (DMSO = 39.52 ppm). NMR splitting patterns are as follows: s = singlet, d = doublet, t = triplet, q  = quartet, multiplet, br = broad. All coupling constants (J) are in hertz (Hz). High-resolution mass spectra (HR/MS) were obtained on an Agilent 6220 LC/MS with an electrospray ionization time-of-flight (ESI-TOF) detector.

### 2D perovskite precursor solutions and thin-film processing procedures

All 2D perovskite precursor solutions were prepared in a nitrogen-filled glovebox. For *n* = 1 2D perovskites, the associated single crystals were dissolved in DMF with a concentration of 0.5 M. For *n* = 4 2D perovskites, stoichiometric ammonium salts, MAI, and PbI_2_ were dissolved in mixed solvent of THF and ACN (3:1) with a concentration of 0.8 M. Perovskite thin films were prepared by spin-coating the precursor solution at 3000 rpm for 30 s, with an acceleration rate of 1000 rpm/s. The films were then thermally annealed at 100 °C for 1 h to remove residual solvent. The thermal annealed films were put on a 70 °C hotplate covered by a glass jar for one hour. An IPA reservoir was used to produce IPA vapor into the solvent annealing jar. The flow rate of IPA vapor was calculated to be 7.85 g h^−1^.

### Device fabrication

Perovskite solar cells were fabricated on pre-patterned FTO glass substrates. The substrates were cleaned with deionized water, acetone, and isopropanol and UV-Ozone cleaner, each step taking 15 min. A compact titanium dioxide (TiO_2_) layer of about 40 nm was deposited by spray pyrolysis of 7 mL 2-propanol solution containing 0.6 mL titanium diisopropoxide bis(acetylacetonate) solution and 0.4 mL acetylacetone at 450 °C using oxygen as the carrier gas. On top of this layer, about 150 nm of mesoporous TiO_2_ layer was formed by spin-coating TiO_2_ paste diluted in ethanol (1:5.5 w/w) at 4500 rpm for 20 s and then the stack was sintered at 500 °C for 30 min. The active layer was deposited by spin-coating, with the same conditions that were used to prepare the perovskite films. Subsequently, the hole-transport layer was deposited on top of the active layer by spin-coating Spiro-OMeTAD solution at 4000 rpm for 20 s. The Spiro-OMeTAD solutions were prepared by dissolving the Spiro-OMeTAD in 1 mL chlorobenzene at a concentration of 60 mM, with the addition of 30 mM Li-TFSI from a stock solution in acetonitrile and 200 mM of *t*-BP. Finally, the devices were completed by thermal evaporation of 80-nm thick gold as top contacts.

### Characterizations

UV-vis absorption spectra were obtained on an Agilent Technologies Cary 5000 spectrophotometer. PL spectra were measured with an Edinburgh Instruments FLS980 photoluminescence spectrometer. XRD measurements were conducted on a Bruker D8 Discover diffractometer using Cu Kα radiation source (*λ* = 1.54 Å). The step size was 0.01°. GIWAXS measurements were performed at the Complex Materials Scattering (CMS) beamline of the National Synchrotron Light Source II (NSLS-II), Brookhaven National Lab. The X-ray beam with an energy of 13.5 keV shone upon the samples with the incident angles of 0.15° with respect to the Si substrate. A custom-made Pilatus-800K detector was placed at a distance of 257 mm from the sample center to capture 2D-GIWAXS images with an exposure time of 10 s. All 2D-GIWAXS images have been background subtracted.

### Solar cell characterization

The current density‐voltage (*J*‐*V*) characteristics of the photovoltaic devices were measured using a Keithley 2635 source‐measurement unit. A solar simulator with Xenon lamp (300 W) and an AM 1.5 G filter were used as the solar simulator. A Newport reference cell (model 71582) was used for calibration. To calibrate the light intensity of the solar simulator (100 mW cm^−2^), the power of the Xenon lamp was adjusted to match the short‐circuit current density (*J*_SC_) of the reference cell under simulated sunlight to that specified by the manufacturer. A light aperture with the same size as the active area was used to shadow the devices. External quantum efficiency measurements were performed using a 300 W Xenon arc lamp (Newport Oriel), with filtered monochromatic light from a Cornerstone 260 1/4 M double-grating monochromator (Newport 74125). A silicon photodiode (model 71580) calibrated at Newport was used as the reference cell. The step size for EQE measurement is 1 nm. The *J*-*V* measurements of all devices were performed on unencapsulated cells in ambient environment without any preconditioning. The scan speed and dwell time were 0.01 V s^−1^ and 0.05 s (reverse scan: 1.2 to −0.2 V, forward scan: −0.2 to 1.2 V), respectively.

### Mobility measurement

Hole-only devices (Au/perovskite/Au) were fabricated to extract the hole mobility of the active layers. The dark *J*-*V* characteristics of the hole-only devices were measured by a Keithley 2635 source following a reported PV-SCLC procedure (0–3 V scan range, 0.02 V step size, 70 ms pulse with 3-min intervals). The mobility was extracted by fitting the *J*-*V* curves to the Mott-Gurney equation.

### Encapsulation and stability measurement under continuous illumination

Devices were encapsulated in a nitrogen glovebox using a cover glass with an attachment of Dynic HG Sheet Desiccant (SF type, 100 μm thick) and a UV-curable epoxy (Epoxy Technology, OG159-2) applied along its perimeter. The stability study on devices was conducted according to the ISOS-L-1I protocol. Encapsulated devices were mounted on homebuilt printed circuit boards and connected to a Keithley 2401 and a Keithley 2700 with two 7705 multiplexer units. Devices were aged under continuous illumination from a Philips metal-halide lamp (Philips MSR 1200 HR). The lamp spectrum (powder density ≈ 1.1 sun) was monitored by an Ocean Optics spectrometer throughout the entire aging test and no significant changes in the spectrum were observed. A liquid cold plate was used to hold the packaged devices. The temperature of the cold plate was maintained at 30 ± 2 °C for the duration of the experiment. The current density versus voltage (*J-V*) characteristics were measured every 6 min. The initial values at the maximum power point (MPP) were obtained from each *J-V* sweep, and then devices were kept at the MPP during operation by an active load system developed by infinityPV ApS.

### General information on our DFT calculations

DFT calculations are performed using plane-wave basis sets via the Quantum Espresso software package^44^. We employ Ultrasoft GBRV pseudopotentials^45^ and PBE exchange correlations (XC)^46^. Dispersion and van der Waals interactions are accounted for using the Grimme DFT-D3 method^47^. The kinetic energy cutoff for wavefunctions is chosen to be 680 eV while the charge cutoff is ten times larger, as we are using ultrasoft pseudopotentials. To find the self-consistent theoretical structures for perovskites with different ligands we start from experimental structures as an initial guess (naturally the placement of hydrogen in these structures does not have enough accuracy), introduce some random displacements to reduce the symmetry of our structure (to make it easier to capture a global rather than local energy minimum), and then perform variable-cell relaxations (vc-relax), where both the atomic coordinates and (all components of) the cell parameter vectors are optimized to find minimum internal energy structures with both (approximately) zero force and (approximately) zero stress. No constraints in the cell shape, volume, or the molecular bond distances have been introduced and the final structures are “fully-relaxed”. The force convergence criterion is that each force component on any atom is smaller than 10^−4^ Ry/au. The convergence threshold on the pressure is 0.5 Kbar. These high-precision calculations are necessary to get a proper convergence in binding energies as well as the structural properties, as relative to the regular solid-state structures, we have molecular groups as part of the structure. Consequently, the energy landscape has regions with very small energy gradients (mostly corresponding to the rotations of the molecular groups); thus, precise calculations of forces and low enough force convergence thresholds are necessary to properly explore the real local minima of these hybrid organic-inorganic structures. The smearing scheme for the Kohn−Sham orbitals’ occupations is the cold smearing of Marzari and Vanderbilt^48^ with a temperature equal to 5 mRy/*k*_*B*_. For post-processing analysis (e.g., charge density differences plots) we mostly perform self-consistent field calculations (scf) on the vc-relaxed structures; while to generate the projected density of states (PDOS) plots we use non-self-consistent field calculations (nscf) with double k-mesh sampling in each direction and extra empty bands to capture more of the conduction bands region. We use a combination of molecule-in-box and periodic extended systems simulations to achieve various computational purposes. For example, to derive the CSD and structural properties we fully relax the extended full perovskite structures, while to derive molecular orbitals and deformation energy of monomer and dimer spacer molecules we perform molecule in box calculation. In such molecular calculations, the structures are relaxed in a compensating charge background and the perovskite-related structures are not fixed, unless otherwise noted for purpose of a gedankenexperiment.

### Calculating the CSD

The CSD is calculated as the center of charge of the ammonium group measured relative to the inorganic plane (in our *N* = 1 layered structures). Here, the plane equation for the inorganic sheet (passing through the Pbs) is obtained, and the distance of each atom in the ammonium group from this plane (*d*) is calculated. We then multiply this distance by the Bader charge for that atom calculated using the 3D charge density maps and the Henkelman’s code^49^. As we are interested in a quantity that is defined as the relative center of charge with a distance dimensionality, we then divide the aforementioned quantity by the total Bader charge of the ammonium group. In short, the CSD is calculated as ${{{{{\rm{CSD}}}}}}=\frac{({q}_{N}{d}_{N}\,+\,{q}_{{{{{\rm{H1}}}}}}{d}_{{{{{\rm{H1}}}}}}\,+\,{q}_{{{{{\rm{H2}}}}}}{d}_{{{{{\rm{H2}}}}}}\,+\,{q}_{{{{{\rm{H3}}}}}}{d}_{{{{{\rm{H3}}}}}})}{({q}_{N}\,+\,{q}_{{{{{\rm{H1}}}}}}\,+\,{q}_{{{{{\rm{H2}}}}}}\,+\,{q}_{{{{{\rm{H3}}}}}})}$, where H_1_, H_2_, and H_3_ are labels for the ammonium groups proton-like hydrogens. It should be noted that CSD can increase both by uplifting of the ammonium’s *N* (relative to the inorganic sheet), or by rotation of the ammonium group so that the proton-like hydrogens face away from the inorganic sheet.

### Reporting summary

Further information on research design is available in the Nature Research Reporting Summary linked to this article.

## Supplementary information

Supplementary Information

Peer Review File

Reporting Summary

Author Checklist

## Data Availability

Data supporting the findings of this work are provided in the paper and/or the Supplementary Information. The single-crystal X-ray structures data used in this study are available in the Cambridge Crystallographic Data Center (CCDC) database under accession code CCDC 665689 [10.5517/ccqbpvk], CCDC 267398 [10.5517/cc8z7r6], CCDC 746125 [10.5517/cct1dks], CCDC 705087 [10.5517/ccrnprt], CCDC 1841681 [10.5517/ccdc.csd.cc1ztf29], CCDC 1053651 [10.5517/cc14cdrn] and CCDC 1840803 [10.5517/ccdc.csd.cc1zshr0]. Other relevant data can be obtained from the corresponding authors upon request.

## References

[1] Kim G (2020). Impact of strain relaxation on performance of α-formamidinium lead iodide perovskite solar cells. Science.

[2] Min H (2019). Efficient, stable solar cells by using inherent bandgap of α-phase formamidinium lead iodide. Science.

[3] Kojima A, Teshima K, Shirai Y, Miyasaka T (2009). Organometal halide perovskites as visible-light sensitizers for photovoltaic cells. J. Am. Chem. Soc..

[4] NREL. *Best Research-Cell Efficiencies* https://www.nrel.gov/pv/assets/pdfs/best-research-cell-efficiencies-190416.pdf (2019).

[5] Turren-Cruz SH, Hagfeldt A, Saliba M (2018). Methylammonium-free, high-performance, and stable perovskite solar cells on a planar architecture. Science.

[6] Zhang F (2022). Metastable Dion-Jacobson 2D structure enables efficient and stable perovskite solar cells. Science.

[7] Zhao X, Liu T, Loo Y-L (2022). Advancing 2D perovskites for efficient and stable solar cells: challenges and opportunities. Adv. Mater..

[8] Tiep NH, Ku Z, Fan HJ (2016). Recent advances in improving stability of PSCs. Adv. Energy Mater..

[9] Zhao X (2020). A hole-transport material that also passivates perovskite surface defects for solar cells with improved efficiency and stability. Energy Environ. Sci..

[10] Liu T (2019). Enhanced control of self-doping in halide perovskites for improved thermoelectric performance. Nat. Commun..

[11] Zhao X (2019). Extending the photovoltaic response of perovskite solar cells into the near‐infrared with a narrow‐bandgap organic semiconductor. Adv. Mater..

[12] Zhang F (2020). Advances in two-dimensional organic–inorganic hybrid perovskites. Energy Environ. Sci..

[13] Zhao X, Liu T, Kaplan AB, Yao C, Loo Y-L (2020). Accessing highly oriented two-dimensional perovskite films via solvent-vapor annealing for efficient and stable solar cells. Nano Lett..

[14] Bai S (2019). Planar perovskite solar cells with long-term stability using ionic liquid additives. Nature.

[15] Tsai H (2016). High-efficiency two-dimensional Ruddlesden–Popper perovskite solar cells. Nature.

[16] Liang C (2021). Two-dimensional Ruddlesden–Popper layered perovskite solar cells based on phase-pure thin films. Nat. Energy.

[17] Jung EH (2019). Efficient, stable and scalable perovskite solar cells using poly(3-hexylthiophene). Nature.

[18] Cao DH, Stoumpos CC, Farha OK, Hupp JT, Kanatzidis MG (2015). 2D homologous perovskites as light-absorbing materials for solar cell applications. J. Am. Chem. Soc..

[19] Smith IC, Hoke ET, Solis-Ibarra D, McGehee MD, Karunadasa HI (2014). A layered hybrid perovskite solar-cell absorber with enhanced moisture stability. Angew. Chem. - Int. Ed..

[20] Knutson JL, Martin JD, Mitzi DB (2005). Tuning the band gap in hybrid tin iodide perovskite semiconductors using structural templating. Inorg. Chem..

[21] Du K (2017). Two-dimensional lead(II) halide-based hybrid perovskites templated by acene alkylamines: crystal structures, optical properties, and piezoelectricity. Inorg. Chem..

[22] Qi T, Grinberg I, Rappe AM (2011). Band-gap engineering via local environment in complex oxides. Phys. Rev. B.

[23] Liao Z (2018). Metal–insulator-transition engineering by modulation tilt-control in perovskite nickelates for room temperature optical switching. Proc. Natl Acad. Sci. USA.

[24] Mercier N, Poiroux S, Riou A, Batail P (2004). Unique hydrogen bonding correlating with a reduced band gap and phase transition in the hybrid perovskites (HO(CH_2_)_2_NH_3_)_2_PbX_4_ (X = I, Br). Inorg. Chem..

[25] Mercier N (2005). (HO_2_C(CH_2_)_3_NH_3_)_2_(CH_3_NH_3_)Pb2I7: a predicted non-centrosymmetrical structure built up from carboxylic acid supramolecular synthons and bilayer perovskite sheets. CrystEngComm.

[26] Mercier N, Louvain N, Bi W (2009). Structural diversity and retro-crystal engineering analysis of iodometalate hybrids. CrystEngComm.

[27] Kakekhani A, Katti RN, Rappe AM (2019). Water in hybrid perovskites: Bulk MAPbI3 degradation via super-hydrous state. APL Mater..

[28] Ren H (2020). Efficient and stable Ruddlesden–Popper perovskite solar cell with tailored interlayer molecular interaction. Nat. Photonics.

[29] Zhu M (2020). Interaction engineering in organic–inorganic hybrid perovskite solar cells. Mater. Horiz..

[30] Yang, Y. et al. Universal approach toward high-efficiency two-dimensional perovskite solar cells via a vertical-rotation process. *Energy Environ. Sci*. 10.1039/D0EE01833C (2020).

[31] Wu G (2019). Fine multi-phase alignments in 2D Perovskite solar cells with efficiency over 17% via Slow post-annealing. Adv. Mater..

[32] Xu Z (2020). Phase distribution and carrier dynamics in multiple-ring aromatic spacer-based two-dimensional Ruddlesden–Popper Perovskite solar cells. ACS Nano.

[33] Grancini G (2017). One-Year stable perovskite solar cells by 2D/3D interface engineering. Nat. Commun..

[34] Alanazi AQ (2019). Atomic-level microstructure of efficient formamidinium-based perovskite solar cells stabilized by 5-Ammonium valeric acid iodide revealed by multinuclear and two-dimensional solid-state NMR. J. Am. Chem. Soc..

[35] Ashari-Astani N (2019). Ruddlesden–Popper phases of methylammonium-based two-dimensional perovskites with 5-Ammonium valeric acid AVA2MAn–1PbnI3n+1 with n = 1, 2, and 3. J. Phys. Chem. Lett..

[36] Kakekhani A (2018). Nature of lone-pair–surface bonds and their scaling relations. Inorg. Chem..

[37] Fu Z, Yang B, Wu R (2020). Understanding the activity of single-atom catalysis from frontier orbitals. Phys. Rev. Lett..

[38] Passarelli JV (2018). Enhanced out-of-plane conductivity and photovoltaic performance in n = 1 layered perovskites through organic cation design. J. Am. Chem. Soc..

[39] Gao Y (2019). Molecular engineering of organic–inorganic hybrid perovskites quantum wells. Nat. Chem..

[40] Lee J-H, Deng Z, Bristowe NC, Bristowe PD, Cheetham AK (2018). The competition between mechanical stability and charge carrier mobility in MA-based hybrid perovskites: insight from DFT. J. Mater. Chem. C.

[41] Duijnstee EA (2020). Toward understanding space-charge limited current measurements on metal halide perovskites. ACS Energy Lett..

[42] Liu L (2020). Cation diffusion guides hybrid halide perovskite crystallization during the gel stage. Angew. Chem. Int. Ed..

[43] Li X (2019). Two-dimensional dion-jacobson hybrid lead iodide perovskites with aromatic diammonium cations. J. Am. Chem. Soc..

[44] Giannozzi P (2009). QUANTUM ESPRESSO: a modular and open-source software project for quantum simulations of materials. J. Phys. Condens. Matter.

[45] Garrity KF, Bennett JW, Rabe KM, Vanderbilt D (2014). Pseudopotentials for high-throughput DFT calculations. Comput. Mater. Sci..

[46] Perdew JP, Burke K, Ernzerhof M (1996). Generalized gradient approximation made simple. Phys. Rev. Lett..

[47] Grimme S, Antony J, Ehrlich S, Krieg H (2010). A consistent and accurate ab initio parametrization of density functional dispersion correction (DFT-D) for the 94 elements H-Pu. J. Chem. Phys..

[48] Marzari N, Vanderbilt D, De Vita A, Payne MC (1999). Thermal contraction and disordering of the Al(110) surface. Phys. Rev. Lett..

[49] Henkelman G, Arnaldsson A, Jónsson H (2006). A fast and robust algorithm for Bader decomposition of charge density. Comput. Mater. Sci..

