# Supplementary material for: A charge transfer framework that describes supramolecular interactions governing structure and properties of 2D perovskites
Source: Nat Commun. 2022 Jul 8;13:3970. doi: 10.1038/s41467-022-31567-y (PMC9270412; doi:10.1038/s41467-022-31567-y)
Supplement: Supplementary file 1 — Supplementary Information [file 41467_2022_31567_MOESM1_ESM.pdf]

Supplementary Information

A Charge Transfer Framework That Describes Supramolecular Interactions Governing Structure and Properties of 2D Perovskites

Author list: Xiaoming Zhao[‖,1], Melissa L. Ball[‖,2], Arvin Kakekhani[‖,3], Tianran Liu[4], Andrew M. Rappe[3] and Yueh-Lin Loo[1,2,*]

[1] Department of Chemical and Biological Engineering, Princeton University, Princeton, NJ 08544, USA

[2] Andlinger Center for Energy and the Environment, Princeton University, Princeton, NJ 08544, USA

[3] Department of Chemistry, University of Pennsylvania, Philadelphia PA 19104-6323, USA

[4] Department of Electrical Engineering, Princeton University, Princeton, NJ 08544, USA

[‖] These authors contributed equally to the work

*Corresponding author: Yueh-Lin Loo, email: lloo@princeton.edu

Supplementary Figures

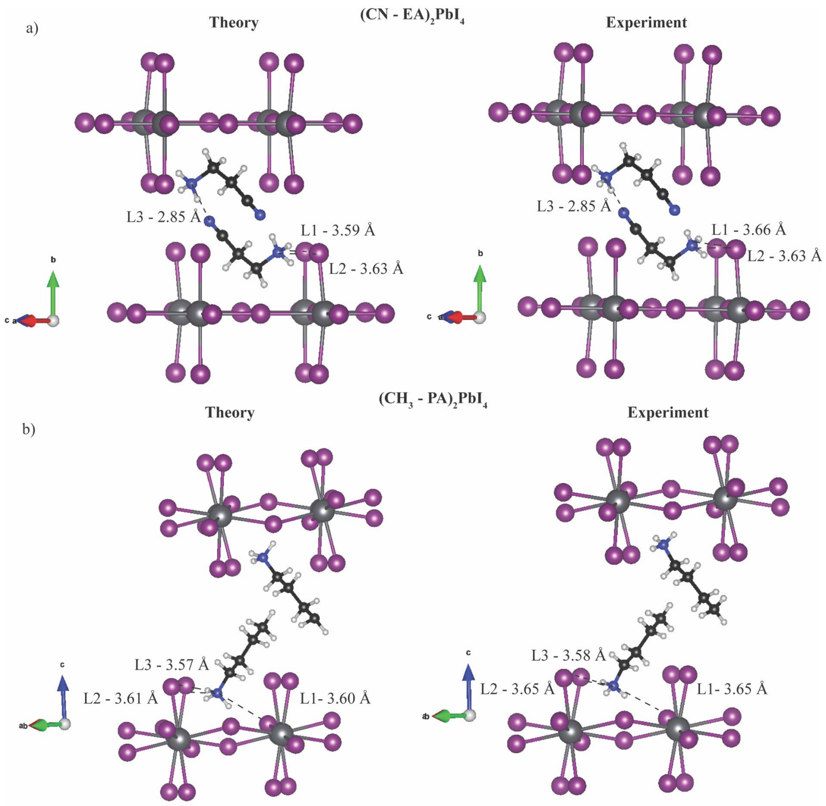

Supplementary Figure 1. Comparison of calculated and experimental .CIF files showing main organic-organic and organic-inorganic hydrogen bonds (distance measured from ammonium nitrogen atom to either the proton-like hydrogen or the iodide moiety) for (CN - EA)$_2$PbI$_4$ and (CH$_3$ - PA)$_2$PbI$_4$. In the latter, the ammonium makes a hydrogen bonding interaction with an in-plane iodide, which does not occur in (CN - EA)$_2$PbI$_4$.

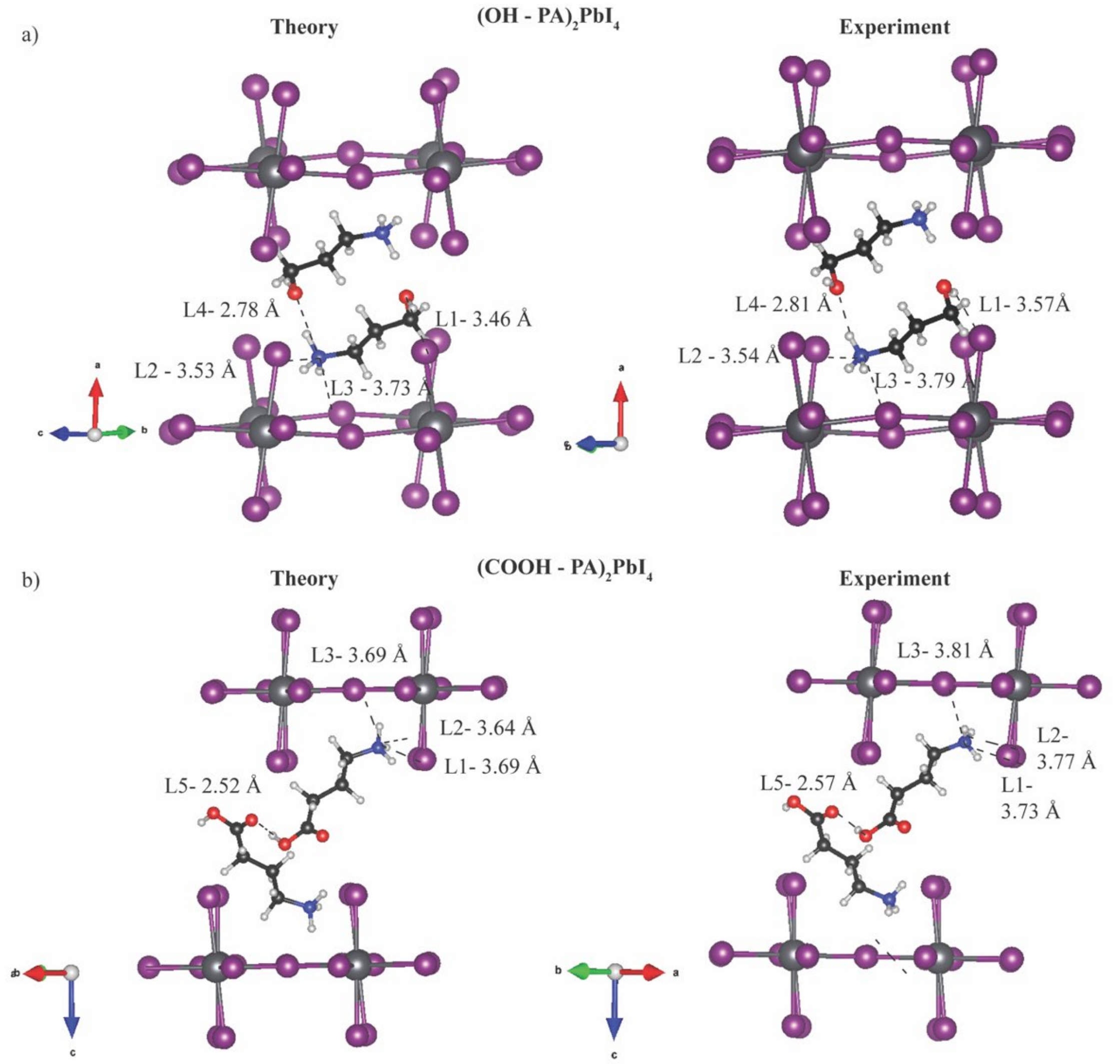

Supplementary Figure 2. Comparison of calculated and experimental .CIF files showing main organic-organic and organic-inorganic hydrogen bonds (distance measured from ammonium nitrogen atom to either the proton-like hydrogen or the iodide moiety) for (OH - PA)$_2$PbI$_4$ and (COOH - PA)$_2$PbI$_4$.

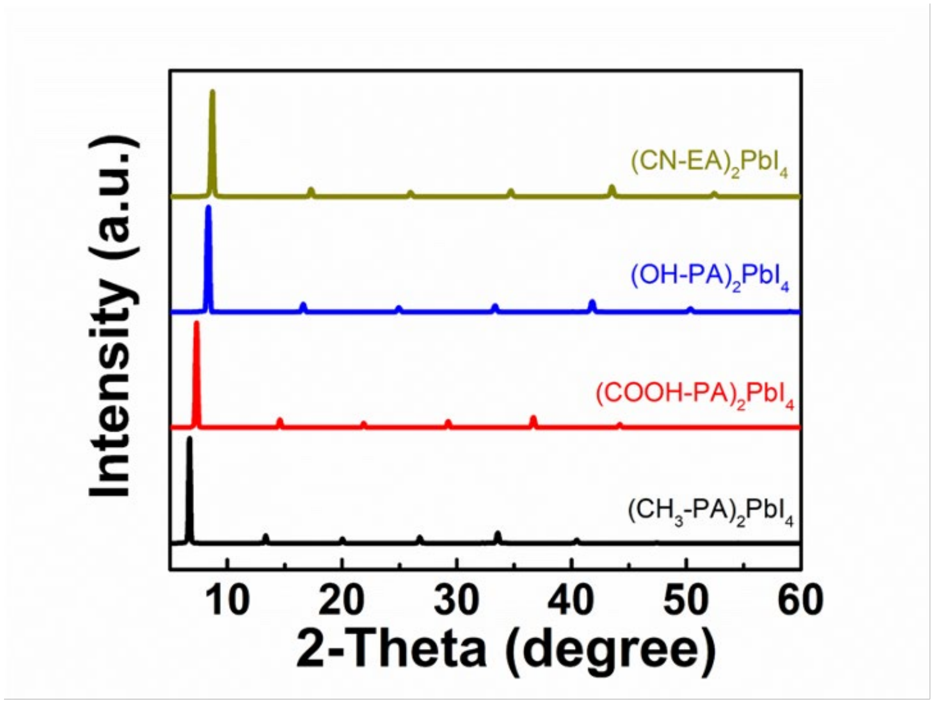

Supplementary Figure 3. XRD patterns of 2D perovskite single crystals.

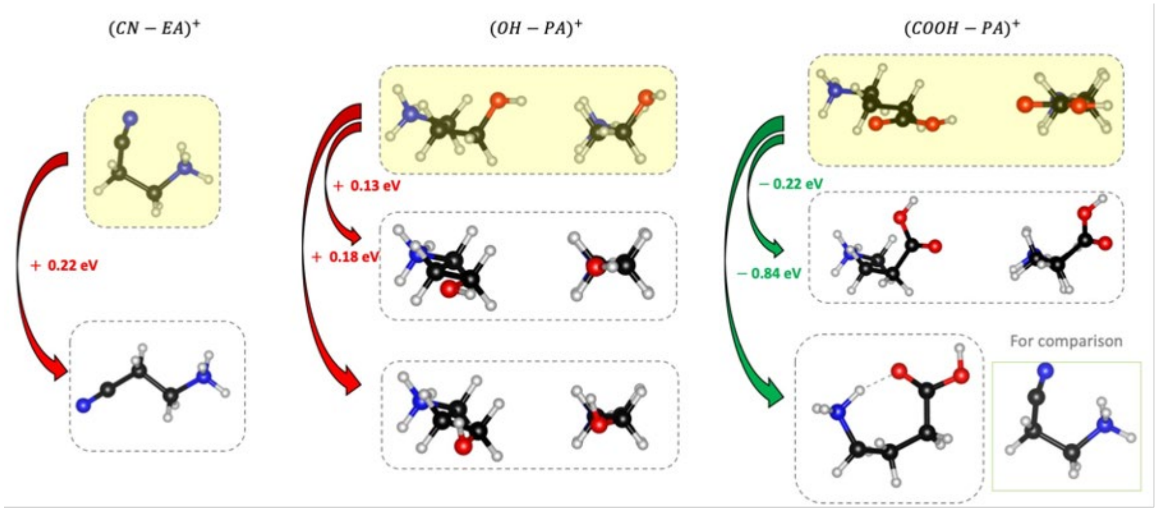

Supplementary Figure 4. Studying the possible conformations for the three functionalized organic cation monomers. The conformations that are closest to the structure of the organic cations once embedded in the perovskite structure are color-coded by yellow. For both CN-EA and OH-PA, such conformation is a low-energy conformation even for a monomer. For CN-EA and OH-PA, this conformation is one that can readily form a dimer structure, as the terminating N or O group is at the same plane as one of the proton-like hydrogens, and away from the rest of the alkylammonium chain. For CN-EA, the curved shape of the alkylammonium chain makes it especially ideal to form a dimer that can lift the ammonium group away from the inorganic plane of the perovskite. For the case of COOH-PA, the conformation closest to the perovskite-embedded one is not the ground state conformation. Also, the perovskite-embedded conformation does not have the proper geometry to form a dimer. The ground-state conformation of COOH-PA can, in principle, form a dimer; nevertheless, the size of the ligand and the existence of both O and OH groups on the functionalized end sterically prohibit formation of such a dimer in the limited space of the perovskite. The COOH-PA forms a molecular zipper structure instead.

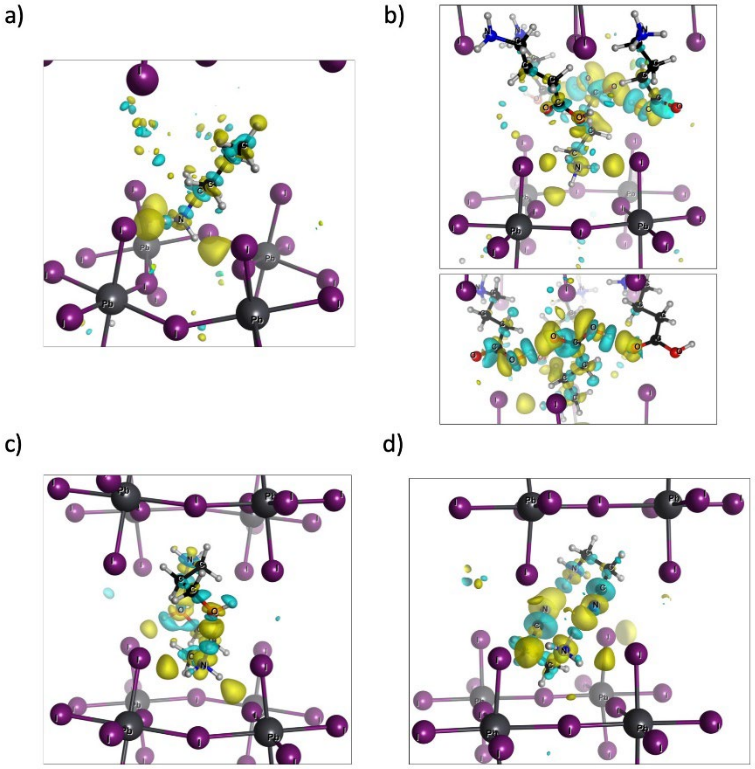

Supplementary Figure 5. Electron density difference (EDD) plots, showing the formation of hydrogen bonds for different organic cations a) $(CH_3-PA)^+$; b) $(COOH-PA)^+$; c) $(OH-PA)^+$; and d) $(CN-EA)^+$, in the layered perovskite structure. For $(COOH-PA)^+$, the upper panel shows the interaction between the ammonium group and the iodines, as well as the smaller interaction with the nearby OH group; the lower panel shows the interaction between the COOH groups in the molecular zipper structure. Here the yellow isosurfaces show electron accumulation, while cyan depicts regions associated with electron depletion (upon introducing an organic group to the system).

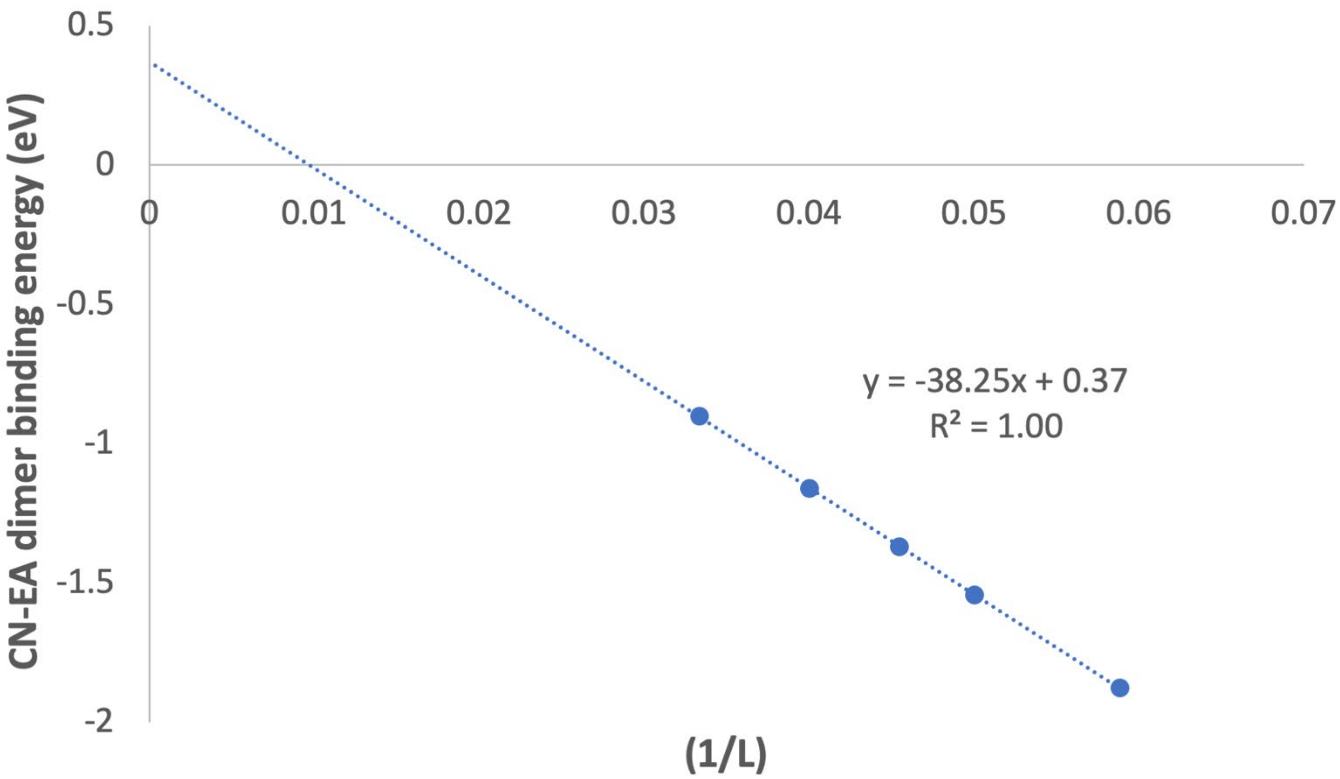

Supplementary Figure 6. The change in the calculated binding energy of $(CN\text{-}EA)^{+}$ dimer as a function of inverse box size in DFT calculations with periodic boundary conditions. For charged systems, periodic DFT calculations add a uniform neutralizing charge background to the simulation unit cell which leads to a Madelung energy term due to artificial interactions associated with this neutralizing charge background. In the limit of an infinite box ($\frac{1}{L} = 0$), i.e., infinitely dilute neutralizing charge background, the binding energy for the cationic (CN-EA) dimer is a positive value. This means without a neutralizing charge background the two monomers do not favor forming a dimer, and the bound-state is merely a local energy minimum.

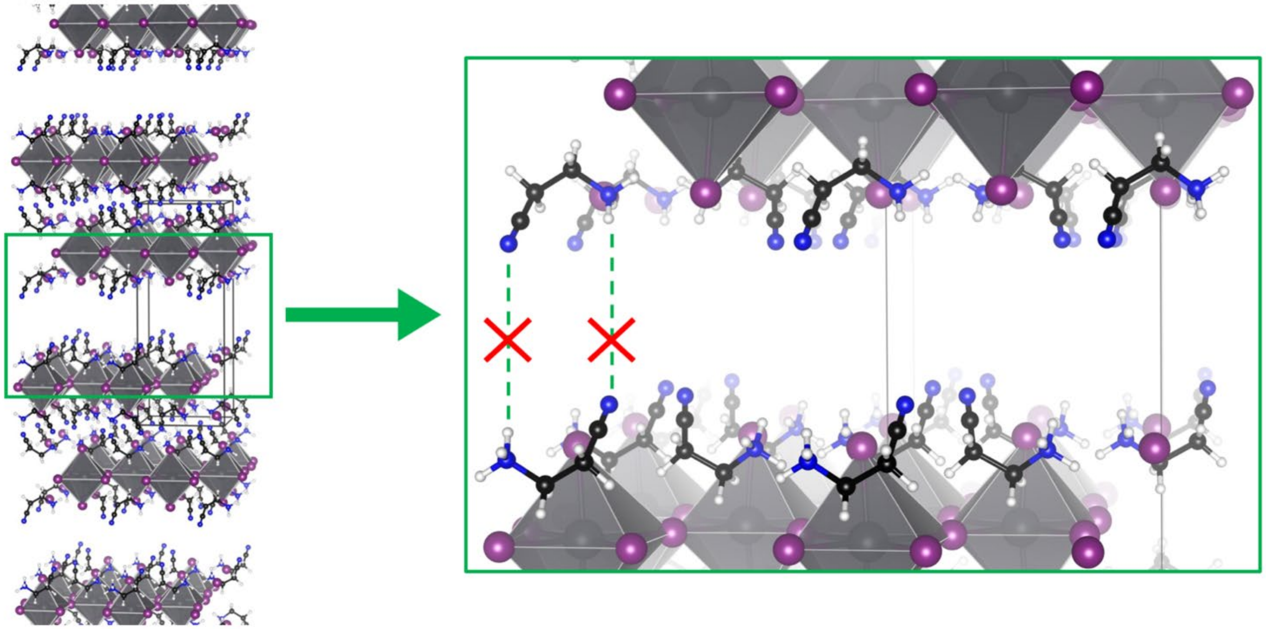

Supplementary Figure 7. A numerical experiment designed to estimate the bond energy for CN-H bonds in the CN-EA dimers (embedded in the perovskite structure). We turn off the van der Waals add-on (Grimme-D3) correction, and calculate the energy of the bare perovskite structure and broken perovskite structures. With van der Waals interactions absent in the calculations the energy difference between the bare and broken (scf calculation with monomers displaced by 5 Å along the perpendicular direction to inorganic plane) perovskite structures, is dominated by the penalty for breaking the dimer bonds. We will add the effect of vdW using another numerical experiment as explained in the Supplementary Note 1 of the SI.

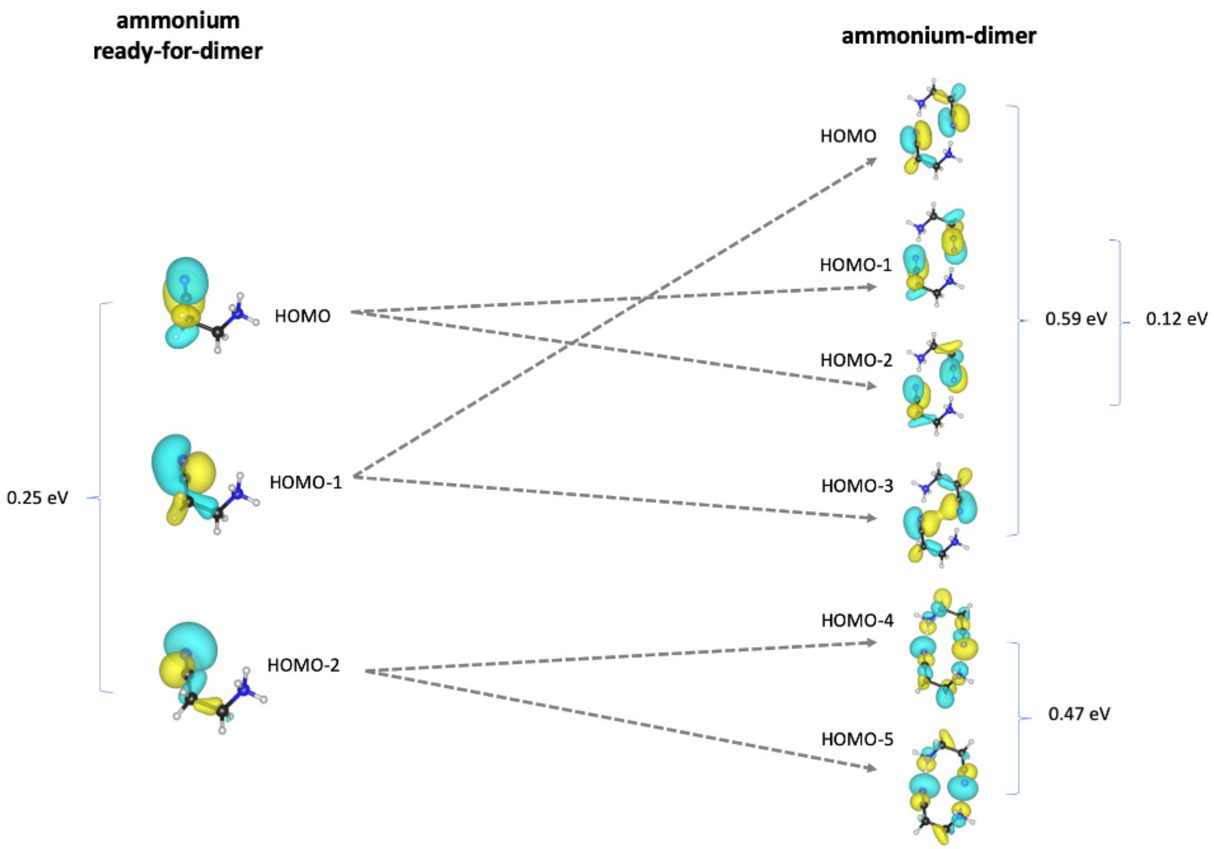

Supplementary Figure 8. Scheme showing how the highest occupied molecular orbitals (MOs) of a ready-to-form-dimer $(CN - EA)^{+}$ split into bonding and anti-bonding combinations, anti-symmetrizing the new wavefunction, upon forming a dimer.

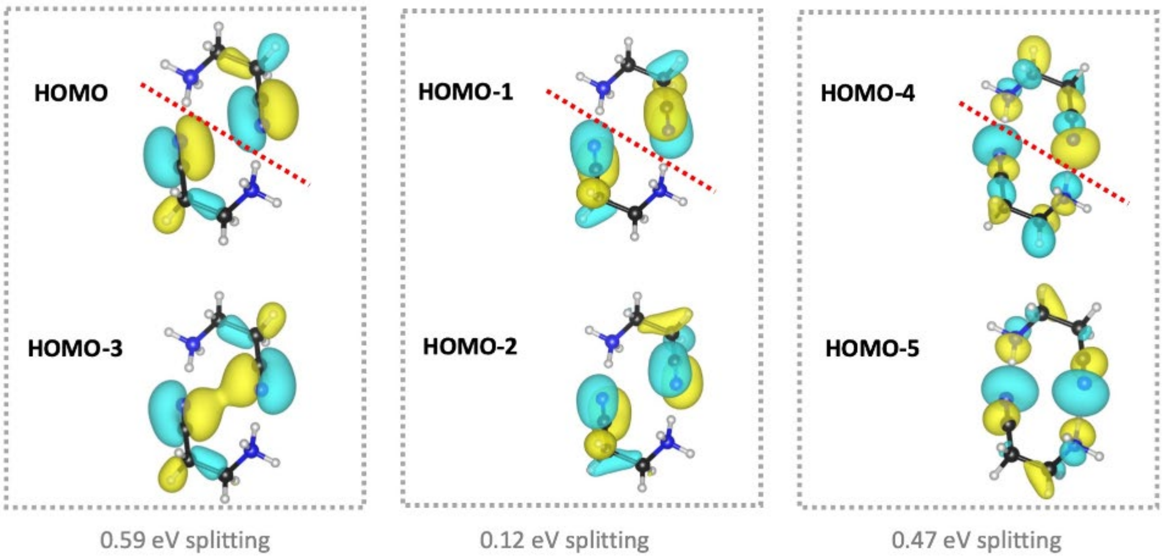

Supplementary Figure 9. The energy splitting between the bonding and anti-bonding states forming the frontier orbitals of the $(CN - EA)^{+}$. The anti-bonding states possess a node (regions of space with zero probability of electrons to be found) on the boundary between the two ammonium groups, where the electrostatics provided by the proton-like hydrogens are favorable for electrons, and thus higher in energy. For orbitals which are in near distance (left and right panel) and point more directly at each other (the left panel) the energy splitting is maximal.

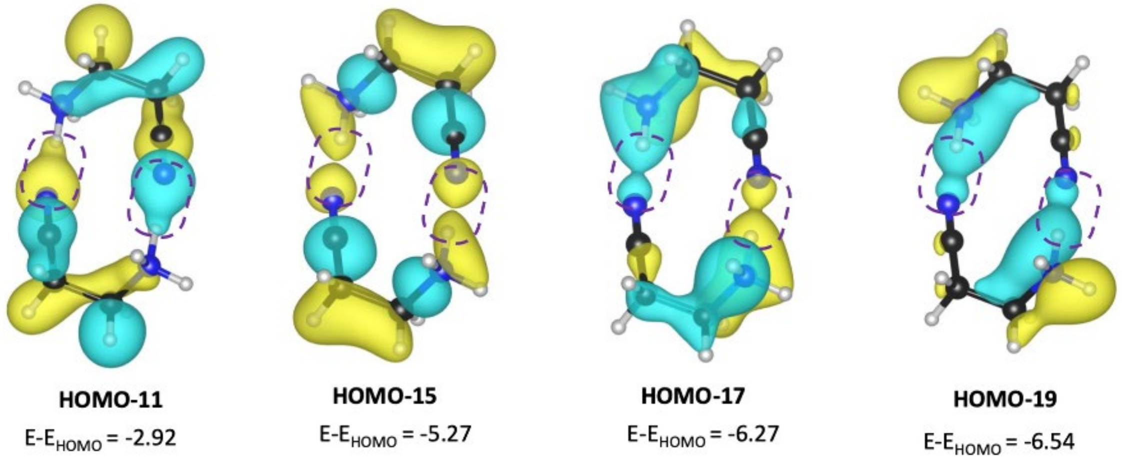

Supplementary Figure 10. The anti-symmetrized wavefunctions of the $(CN - EA)^+$ dimers for relatively deep states below the frontier orbitals. It can be seen that bonding states between the two ligands form, connecting the N group in the cyano group to the proton-like hydrogen on the opposite ammonium group.

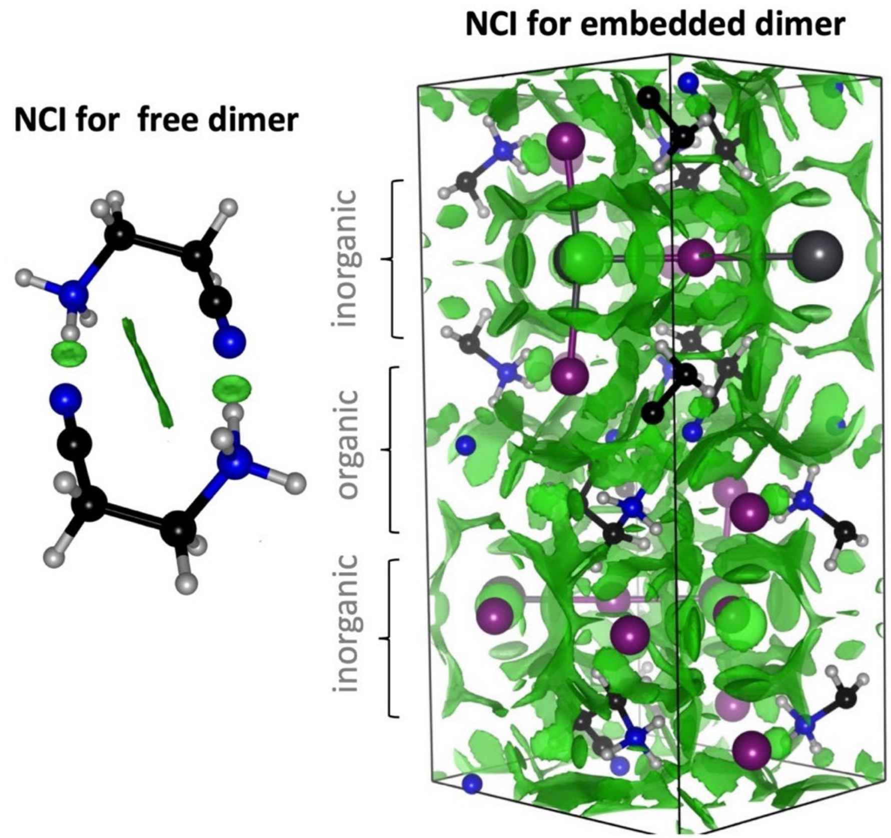

Supplementary Figure 11. Non-covalent interactions for (on the left) a CN-EA$^+$ dimer in uniform charge neutralizing background (jellium), and (on the right) the whole hybrid perovskite, shown by green regions corresponding to an NCI (isovalue = 0.5). For the free dimer, on the left, the regions of non-covalent interactions correspond to the electrostatically-driven bond between the proton-like hydrogen and the cyano group, and the center of the dimer which corresponds to dipole-dipole interactions. For the perovskite system, the non-covalent interactions in form of dispersion interactions are prevalent and almost isotropic; thus, in addition to the additional hydrogen bonds (with axial iodides), the dimers get stabilized by dispersion interactions once embedded in the perovskite (rather than their free form)

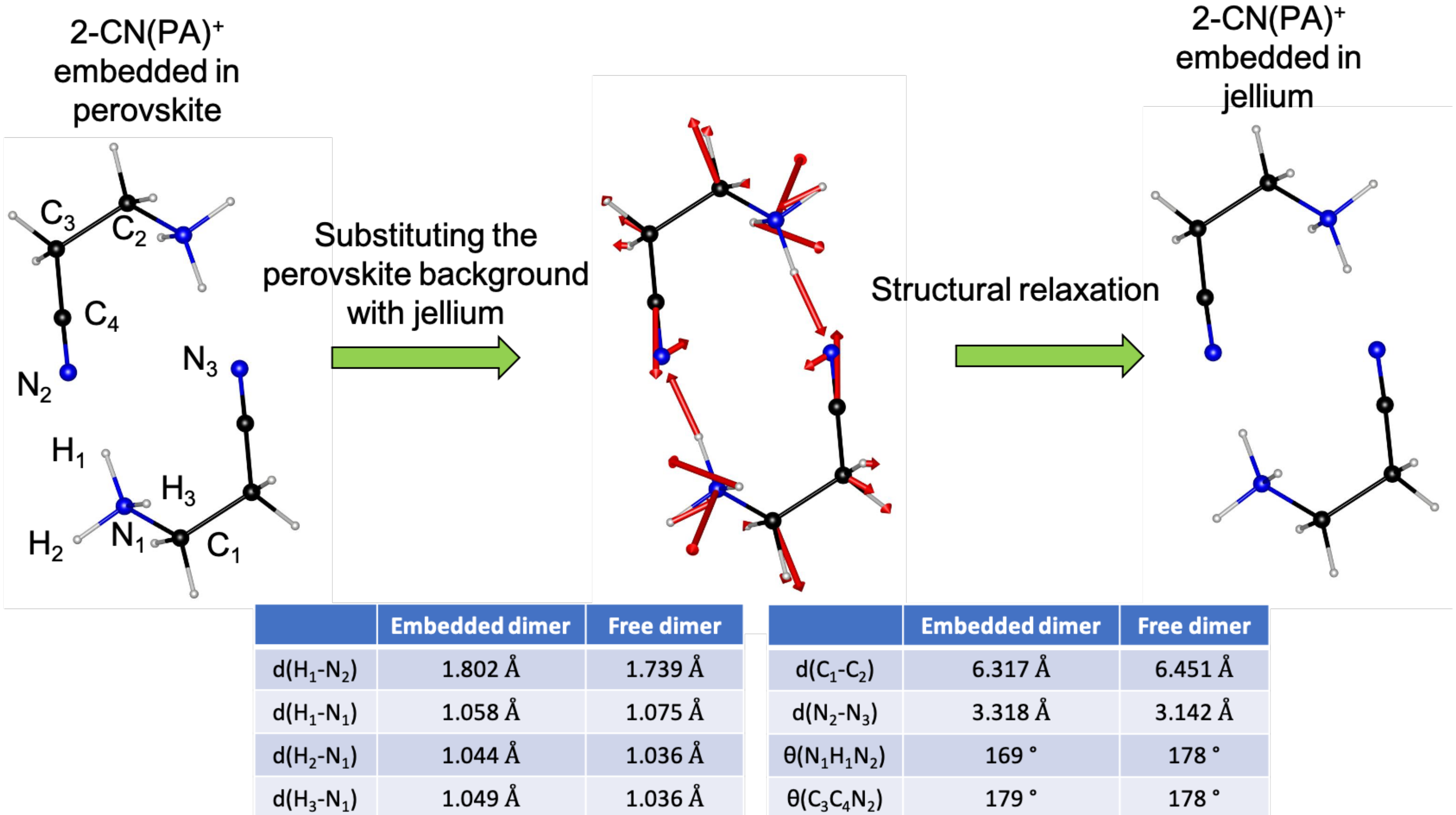

	Embedded dimer	Free dimer
d(H_1-N_2)	1.802 Å	1.739 Å
d(H_1-N_1)	1.058 Å	1.075 Å
d(H_2-N_1)	1.044 Å	1.036 Å
d(H_3-N_1)	1.049 Å	1.036 Å

	Embedded dimer	Free dimer
d(C_1-C_2)	6.317 Å	6.451 Å
d(N_2-N_3)	3.318 Å	3.142 Å
θ($N_1H_1N_2$)	169 °	178 °
θ($C_3C_4N_2$)	179 °	178 °

Supplementary Figure 12. Changes in the structure of 2-CN(EA)$^+$ dimer as a result of removing the perovskite background and substituting it with a uniform neutralizing charge background. On the left: two cations forming dimer and embedded in perovskite. In the middle: substituting the perovskite background with jellium, and calculating the resultant forces (red arrows) on each atom. On the right: relaxed structure of 2-CN(EA)$^+$ dimer.

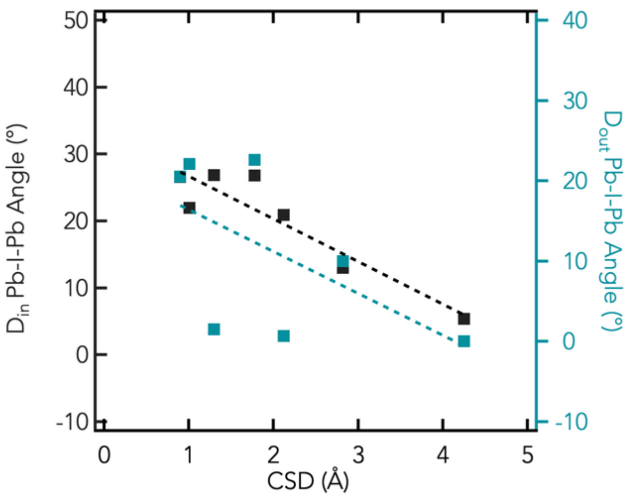

	D_{in} (°)	D_{out} (°)	D_{tilt} (°)	CSD (Å)
CH3	21.936	22.083	30.755	1.01
COOH	20.869	0.679	20.879	2.12
OH	12.963	10.002	16.321	2.82
CN	5.358	0.000	5.358	4.25
PEA	26.571	20.508	33.131	1.78
NAPH	26.784	22.614	34.542	0.90
BDA	26.840	1.523	26.880	1.30

Supplementary Figure 13. CSD verse in- and out-of-plane components of the octahedral tilt.

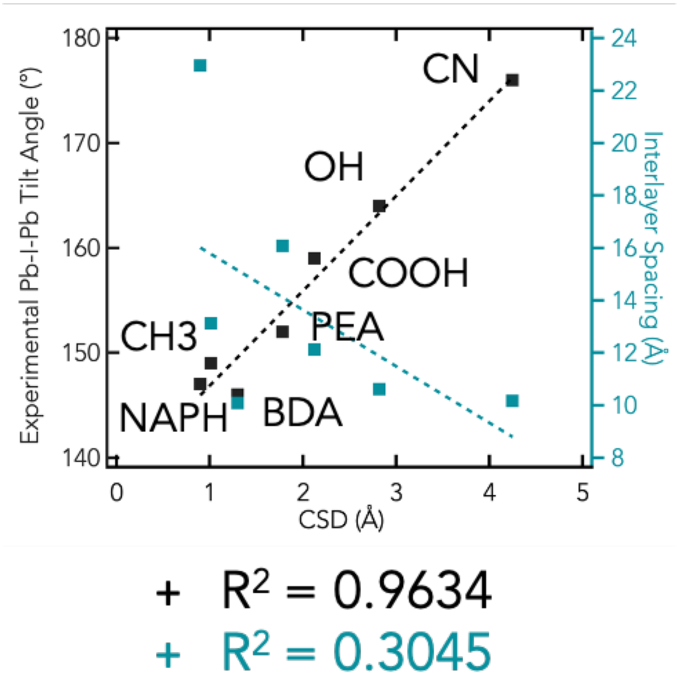

	d-spacing (Å)	Length of ligand (Å)
CH3-PA	13.12	4.91
COOH-PA	12.13	6.13
OH-PA	10.62	4.29
CN-EA	10.17	3.97
PEA	16.07	5.03
BDA	10.10	5.67
NAPH	22.95	8.24

Supplementary Figure 14. Correlations between CSD descriptor and structural properties for a broader set of A-cation molecules. Pb-I-Pb angle correlates well with CSD, while the interlayer spacing is weakly correlated. Across the broad set of ligands, interlayer spacing also only weakly correlates to materials properties band gap and hole mobility.

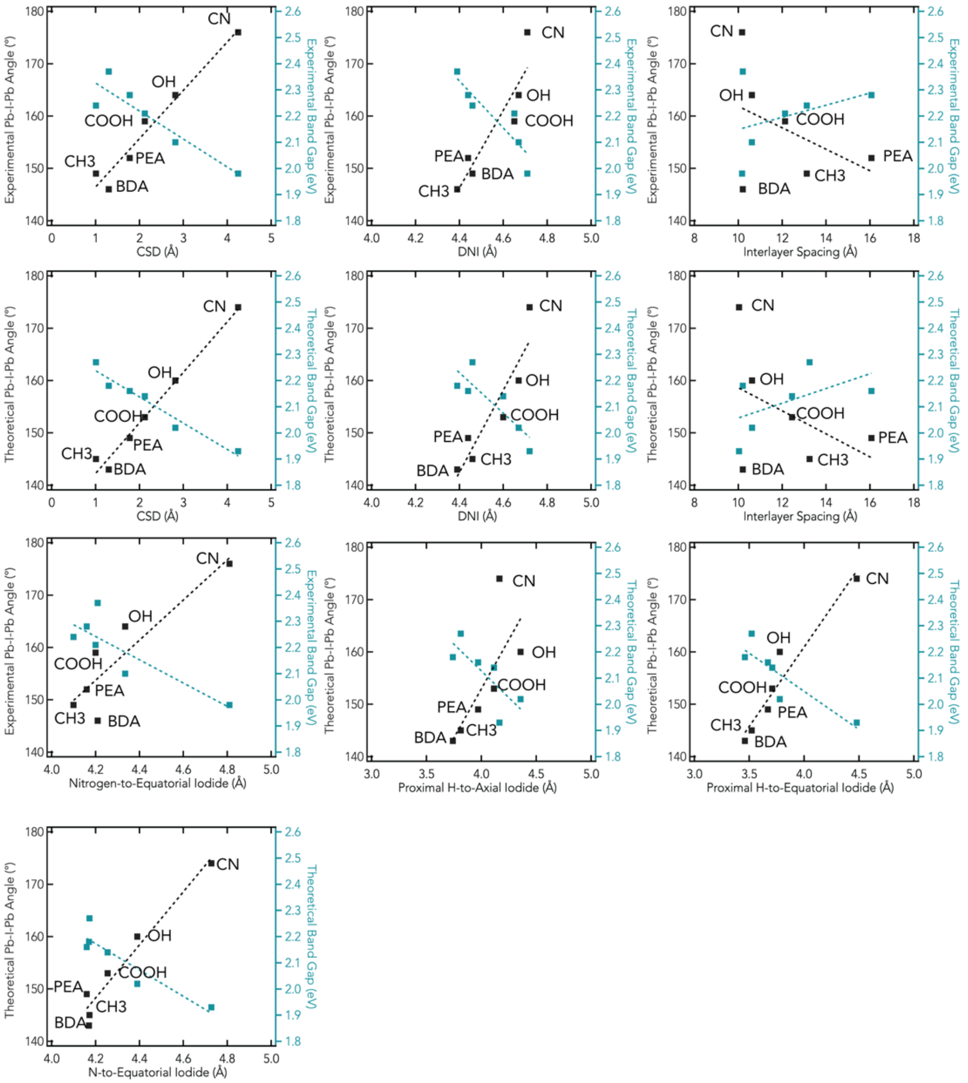

Supplementary Figure 15. Correlations between various descriptors and octahedral tilt and band gap. We also include summary tables with the respective R^2 values for each of the fits below.

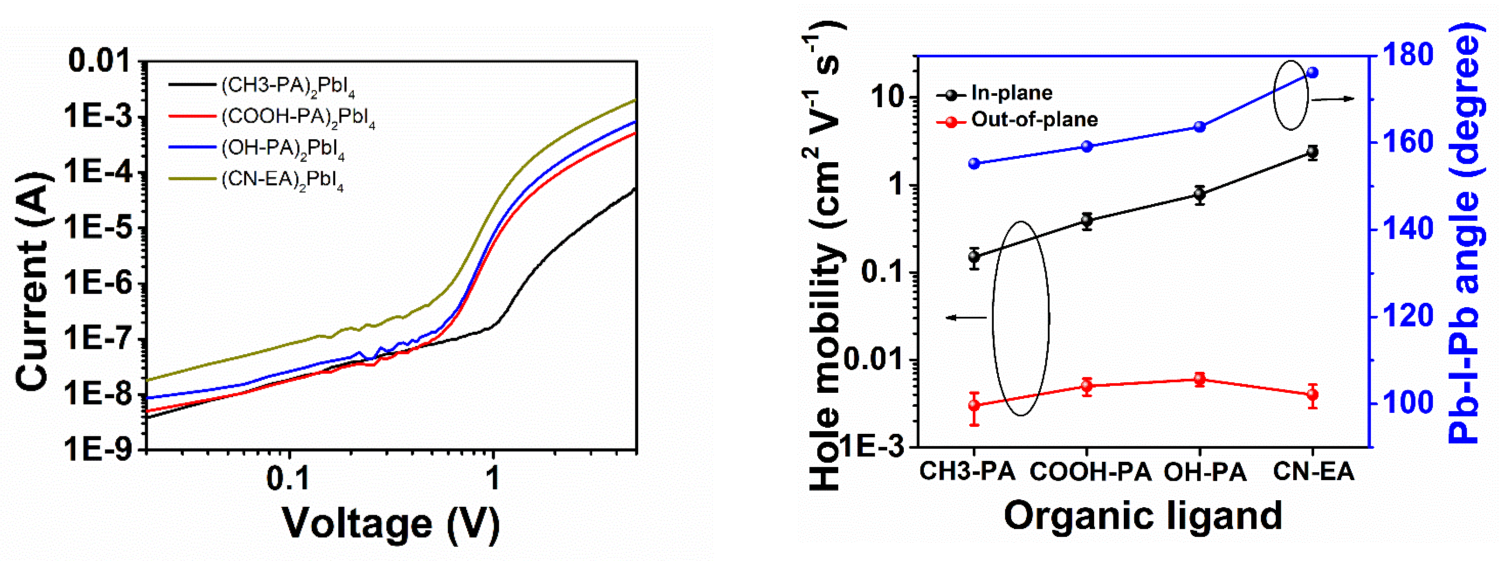

Supplementary Figure 16. (left) *I-V* characteristics in the dark of hole-only devices for four perovskite structures; (right) in-plane and out-of-plane hole mobility of 2D perovskite single crystals containing different organic ligands. Average and standard deviation values were obtained on 16 devices fabricated and tested under the same conditions. Supplementary Table 6 contains these average and standard deviation values.

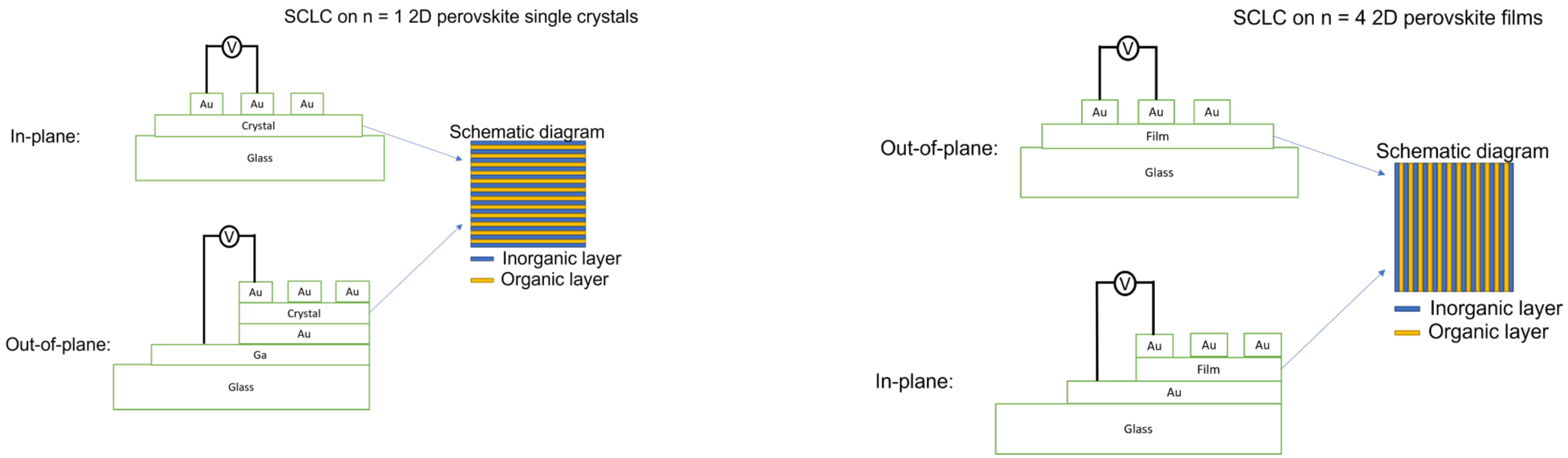

Supplementary Figure 17. Structure of hole-only devices to extract hole mobilities of 2D perovskite single crystals for n = 1 (left) and n = 4 thin films (right).

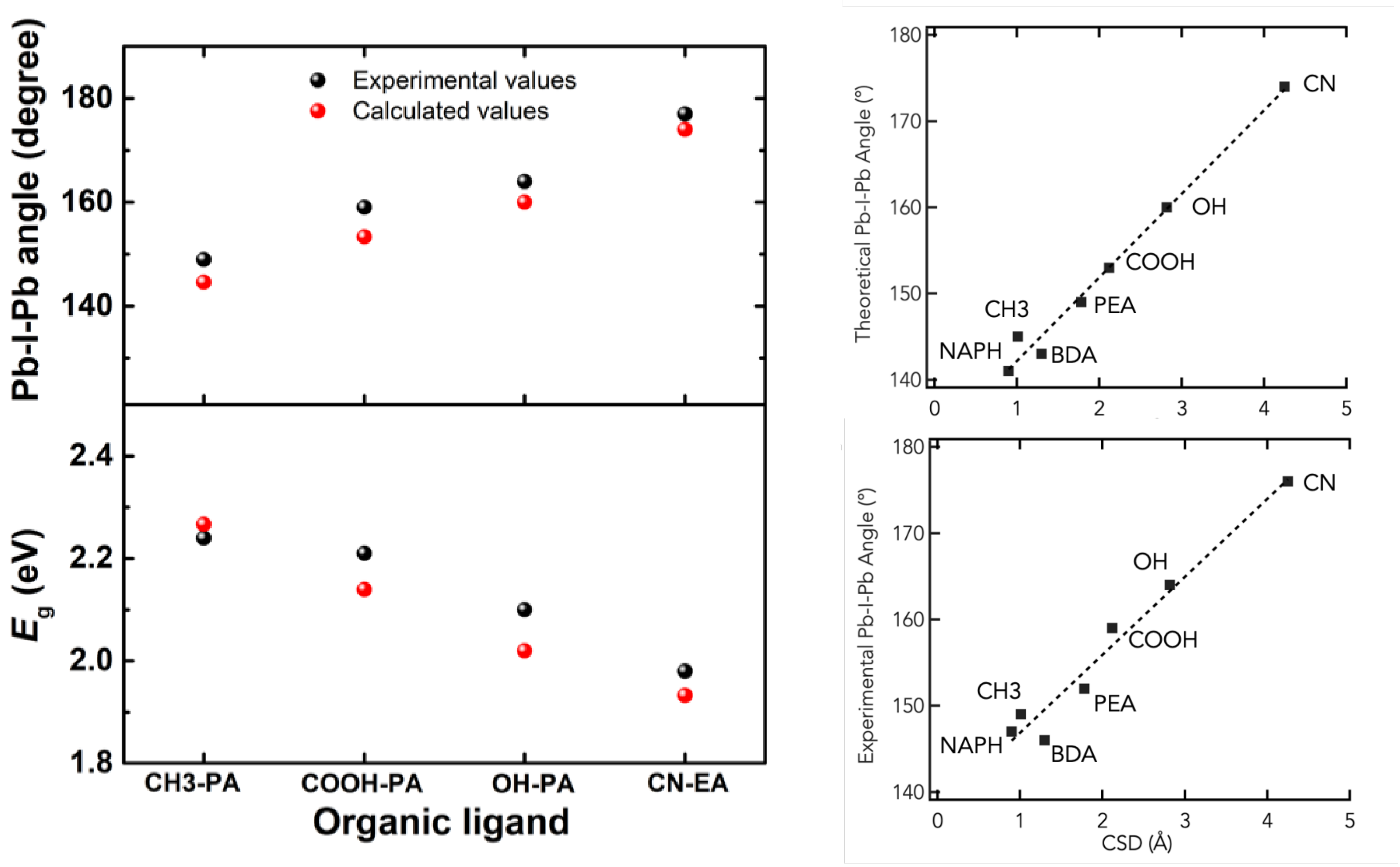

Supplementary Figure 18. Both the experimentally- and theoretically-determined E_g and Pb-I-Pb angles correlate with CSD values. In addition, the experimental and theoretical values are in good agreement.

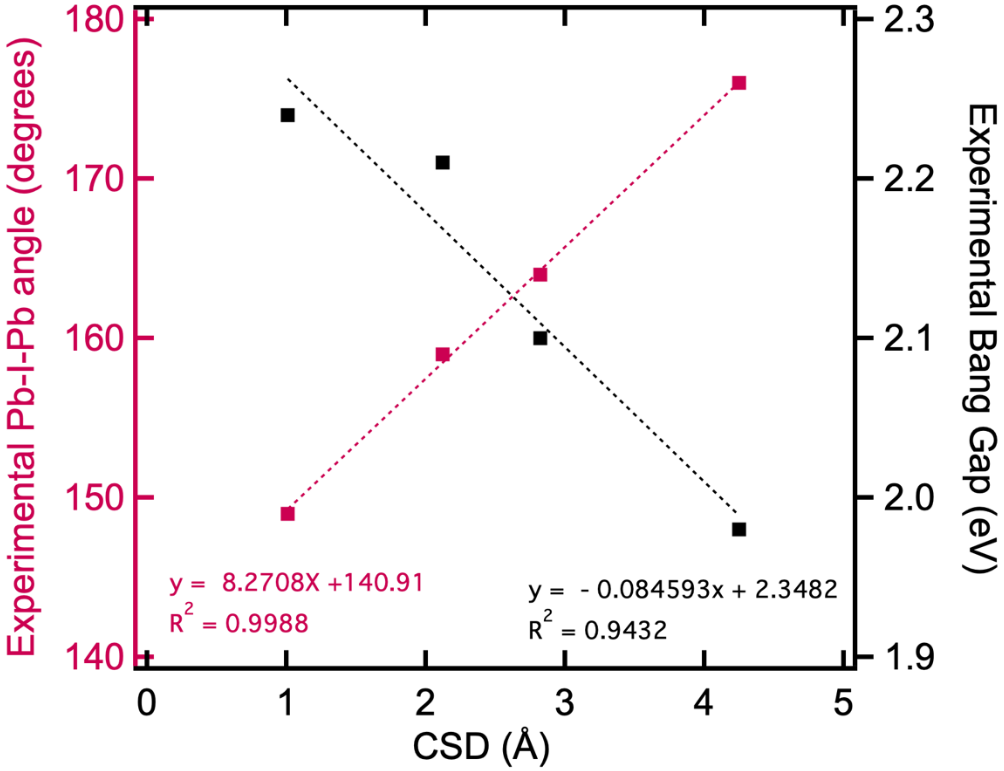

Supplementary Figure 19. The experimental Pb-I-Pb angle *vs*. CSD plot showing a strong correlation between the two parameters, analogous to the theoretically-determined Pb-I-Pb angles. CSD values are 1.01 Å = $(CH_3 – PA)_2PbI_4$; 2.12 Å = $(COOH – PA)_2PbI_4$; 2.82 Å = $(OH – PA)_2PbI_4$; and 4.25 Å = $(CN – EA)_2PbI_4$.

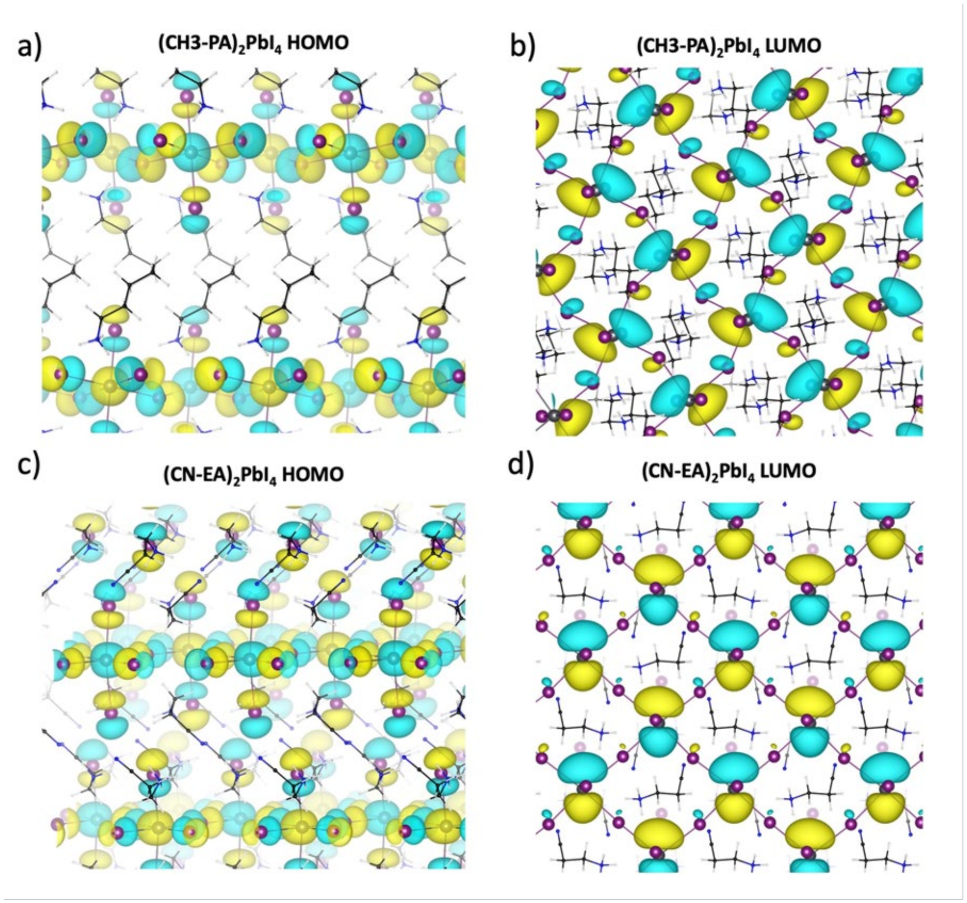

Supplementary Figure 20. The HOMO and LUMO wavefunctions plotted for (CH_3-PA) and (CN-EA) based N=1 layered hybrid perovskite. The HOMO are antibonding combinations of Pb_{6s} and I_{5p} states, while the LUMO is mostly dominated by the Pb_{6p} states. Different colors of isosurfaces indicate the phase of the wavefunction.

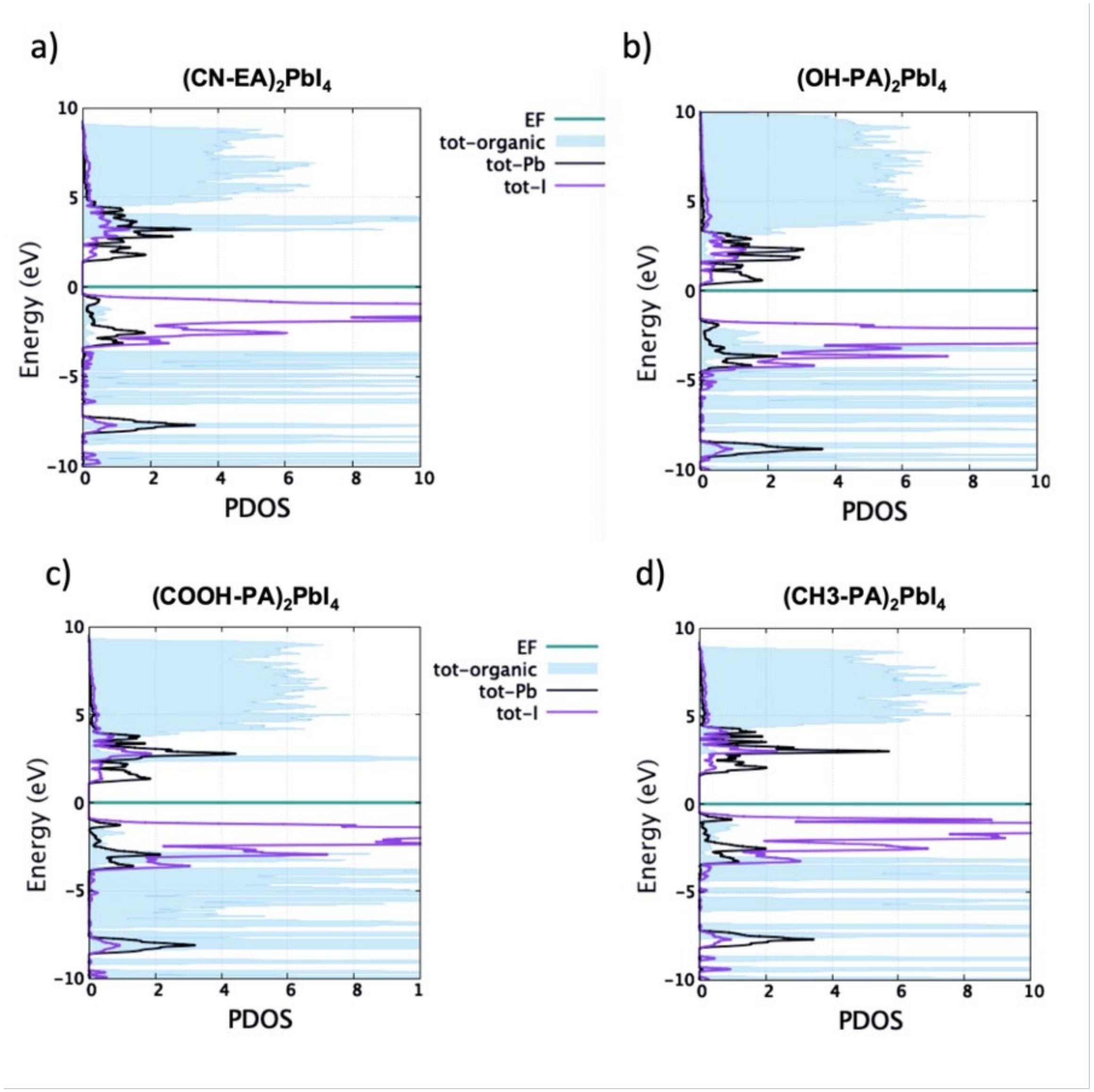

Supplementary Figure 21. Projected density of states (PDOS) for *N*=1 layered hybrid perovskites based on different organic cations. The VBM and CBM are mostly dominated by the inorganic backbone.

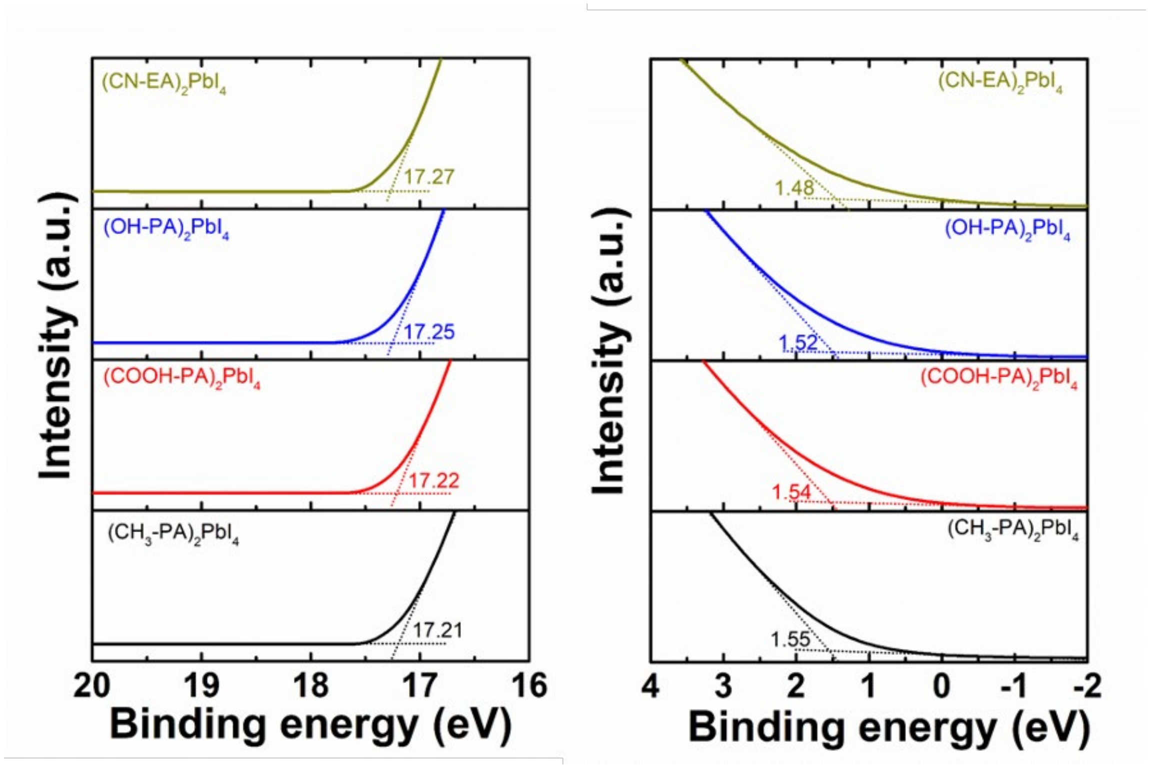

Supplementary Figure 22. UPS spectra of 2D perovskite single crystals in (left) secondary electron cut-off region and (right) valence band region.

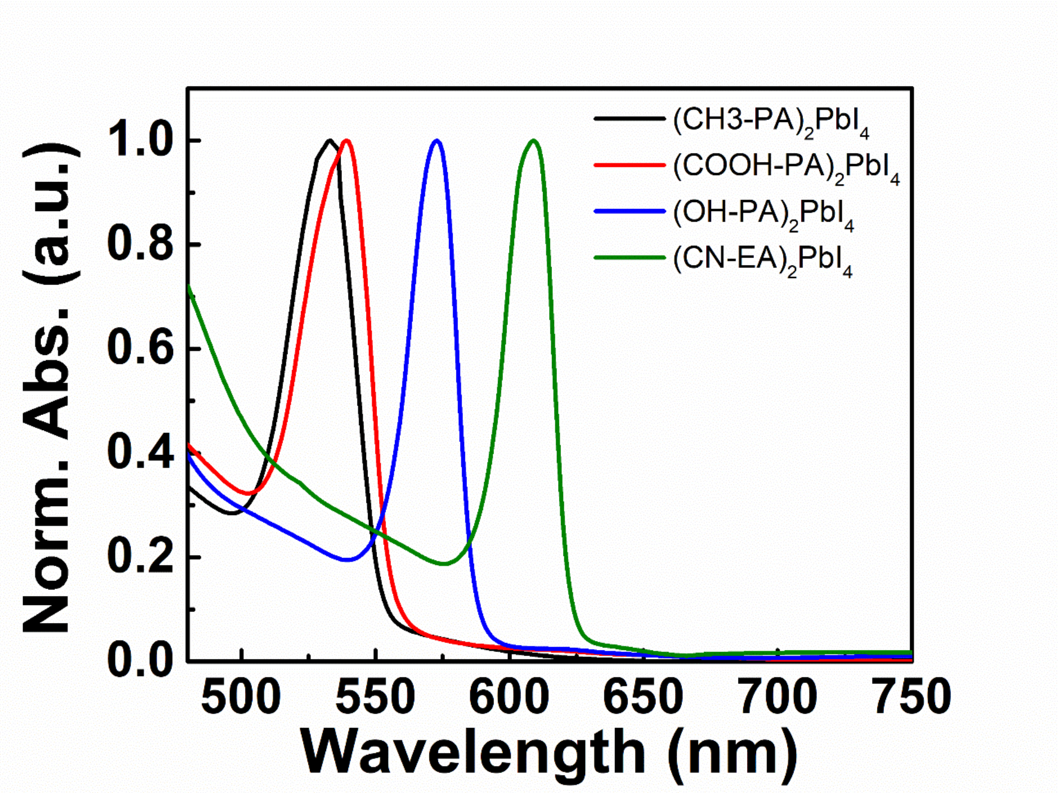

Supplementary Figure 23. Absorption spectra of 2D perovskite single crystals.

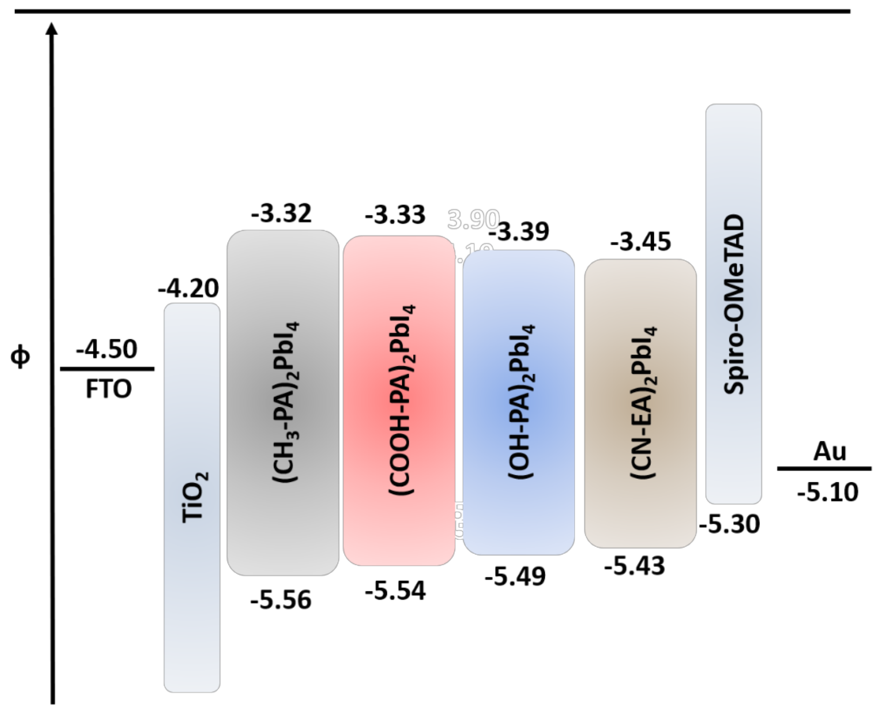

Supplementary Figure 24. Energy diagram for 2D perovskite single crystals relative to HTL/ETL layers and electrodes.

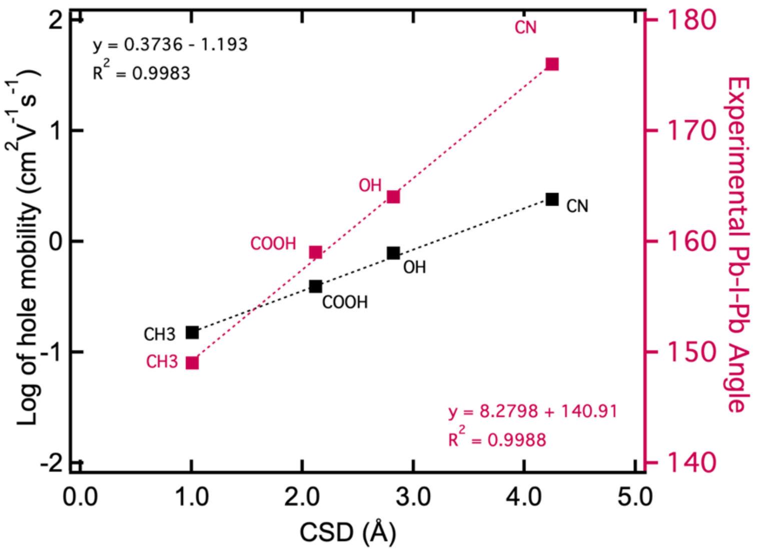

Supplementary Figure 25. A plot showing the correlation between CSD and the log of in-plane hole mobility and experimental Pb-I-Pb angle of n = 1 2D perovskites.

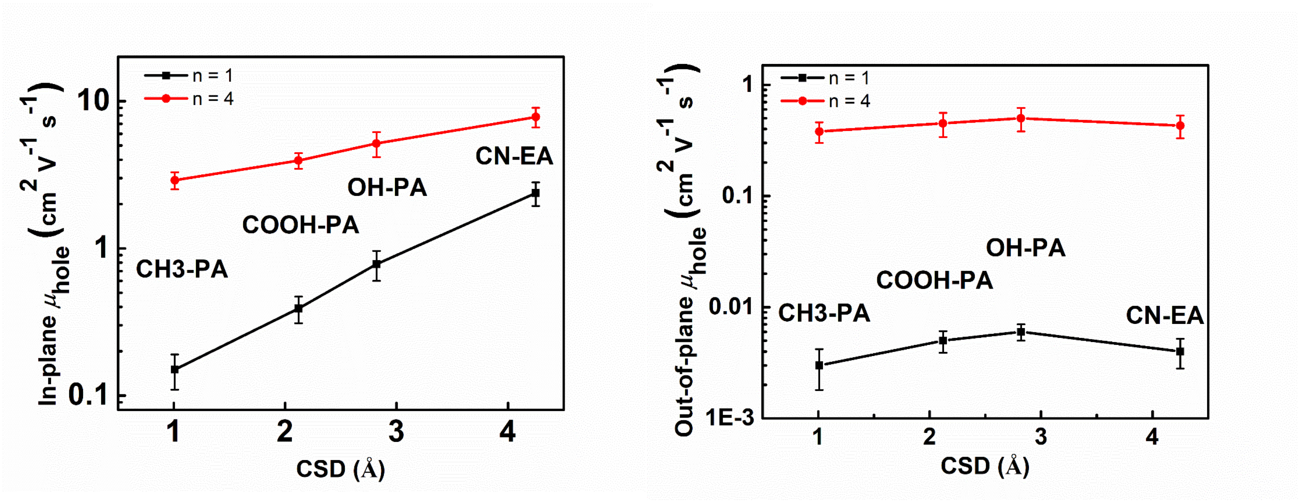

Supplementary Figure 26. In-plane and out-of-plane hole mobility for the four ligands studied. Single crystals for n = 1 and thin films for n = 4. Average and standard deviation values were obtained on 16 devices fabricated and tested under the same conditions. Supplementary Table 7 contains these average and standard deviation values.

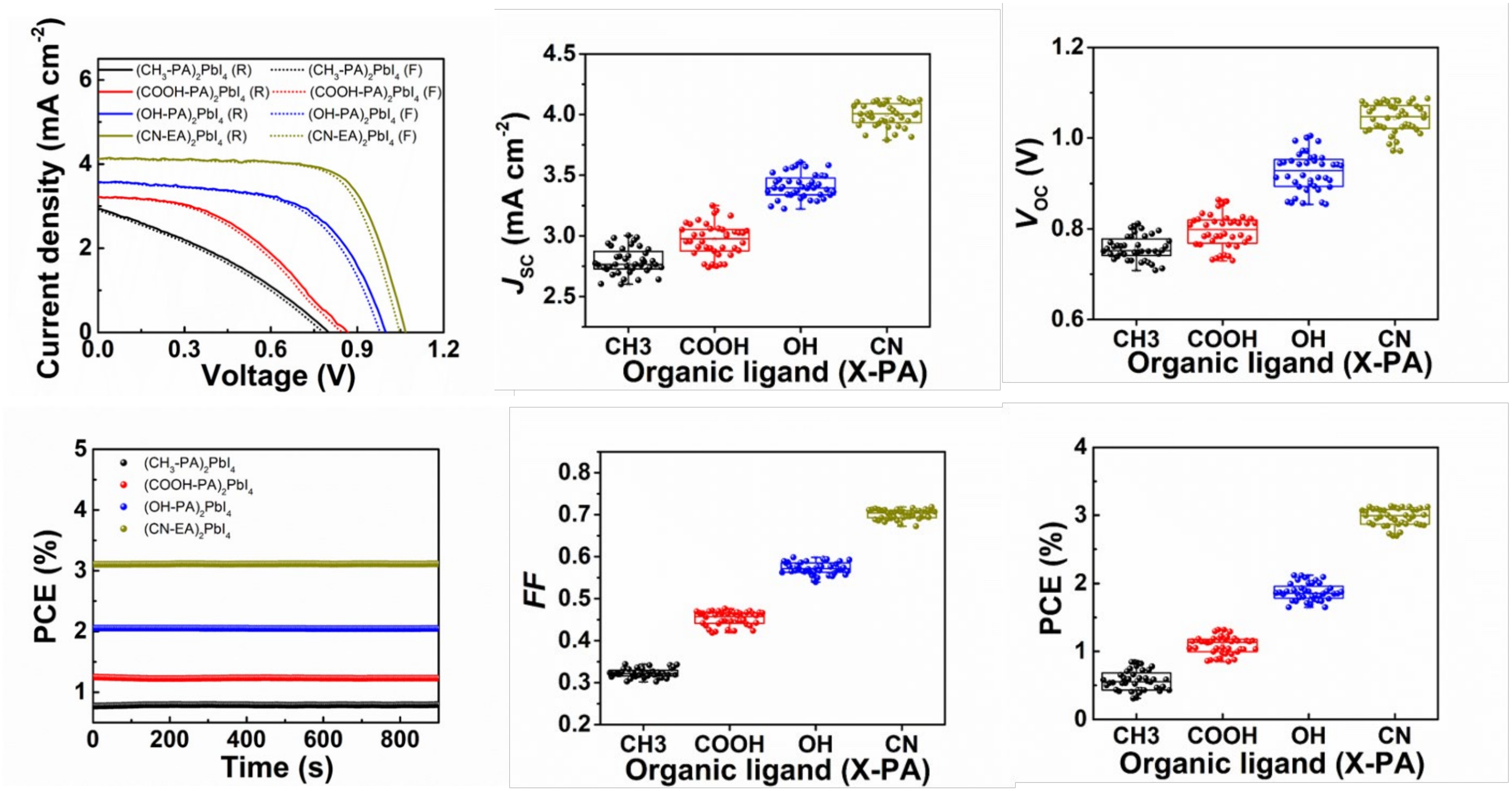

Supplementary Figure 27. *J-V* characteristics under forward (F) or reverse (R) voltage scan direction of and stabilized PCEs of solar cells using n=1 2D perovskite thin films. Solar-cell performance statistics of PSCs with n=1 2D perovskite thin films. Values were extracted from testing 40 devices at each condition. Note: the organic ligand is CN-EA is denoted CN in these graphs.

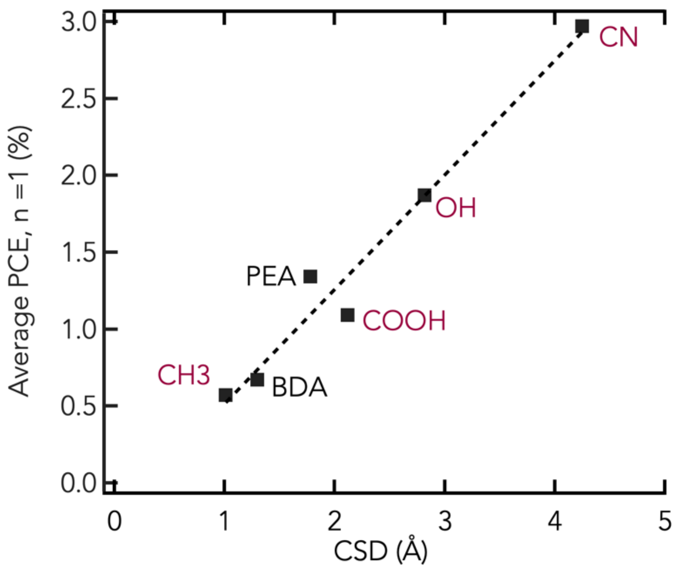

Supplementary Figure 28. PCE *vs*. CSD for the four ligands studied and additional ligands from literature. The pink text denotes the four ligands from this study. Although the CSD describes molecular interactions on the molecular length scale, we find it fascinating that the CSD is able to correlate so strongly with device performance, with $R^2 = 0.9662$. These findings further imply that 2D perovskites that exhibit larger CSD tend to result in solar cells with higher device performance (provided that the device characteristics are not limited by extrinsic factors), and we believe that our design strategy is robust and that the results from this study will be of great interest to the perovskite community.

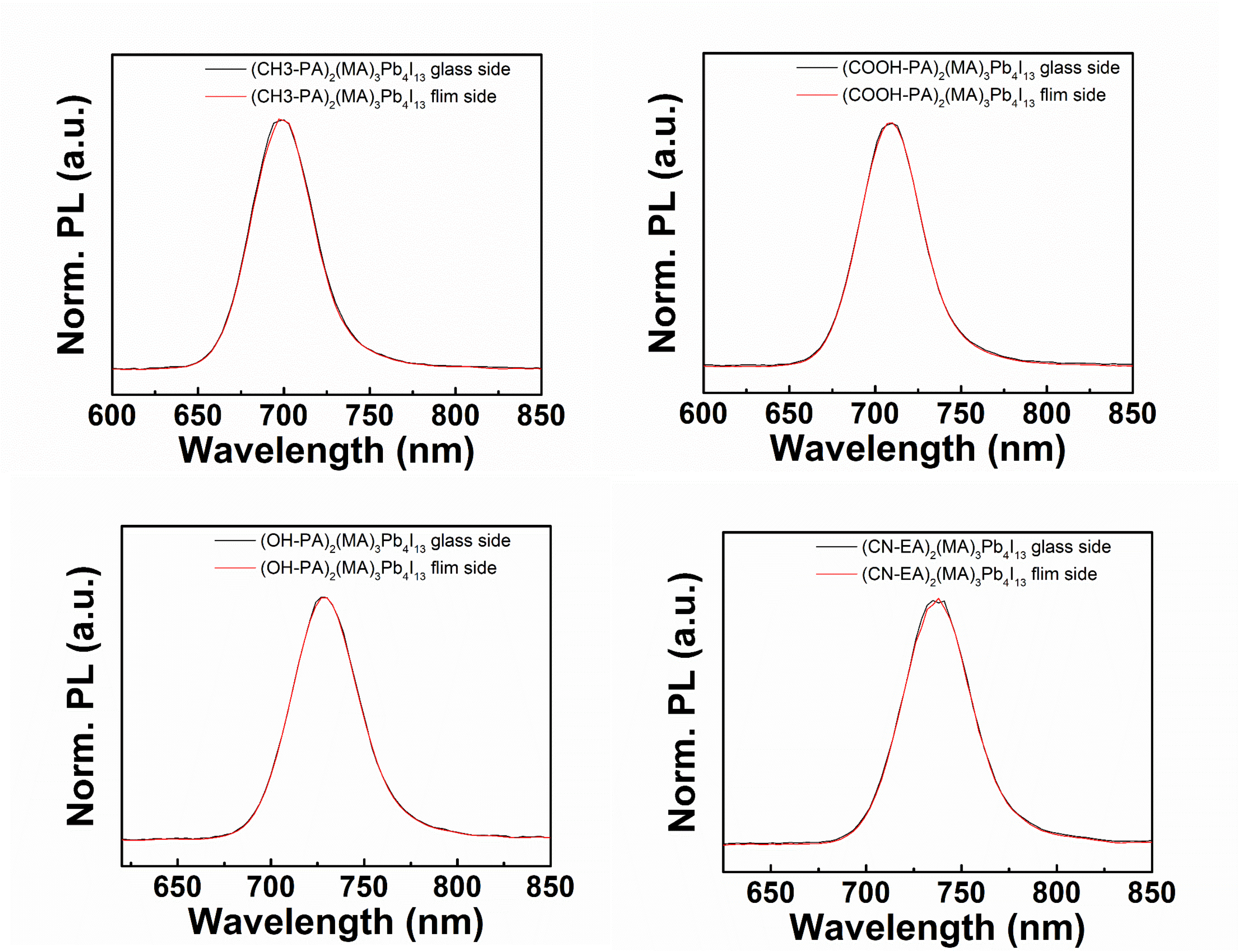

Supplementary Figure 29. PL spectra of n = 4 2D perovskite thin films excited from glass or film side. The PL spectra of our n=4 films do not show significant emission from higher-n-phases of the 2D perovskites or from the n=inf $MAPbI_3$. Furthermore, the PL spectra show strong emission that are identical whether the samples are excited from the glass sides or the film sides, indicating compositional homogeneity throughout the depths of the films.

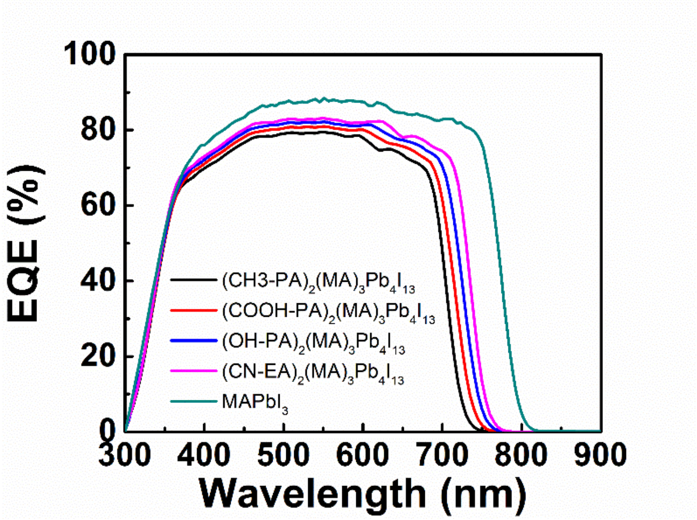

Supplementary Figure 30. EQE spectra of solar cells based on n = 4 2D perovskite thin films and reference $MAPbI_3$. The EQE band edge of our four n=4 2D perovskite solar cells all exhibit more than 40-nm blueshift to solar cells with their 3D counterparts $MAPbI_3$. The extended EQE from $(CH3\text{-}PA)_2(MA)_3Pb_4I_{13}$, $(COOH\text{-}PA)_2(MA)_3Pb_4I_{13}$, $(OH\text{-}PA)_2(MA)_3Pb_4I_{13}$ to $(CN\text{-}EA)_2(MA)_3Pb_4I_{13}$ consistent with their bandgap narrowing. We note that each of the four EQE onsets are at different wavelengths, suggesting there is no contribution from $MAPbI_3$.

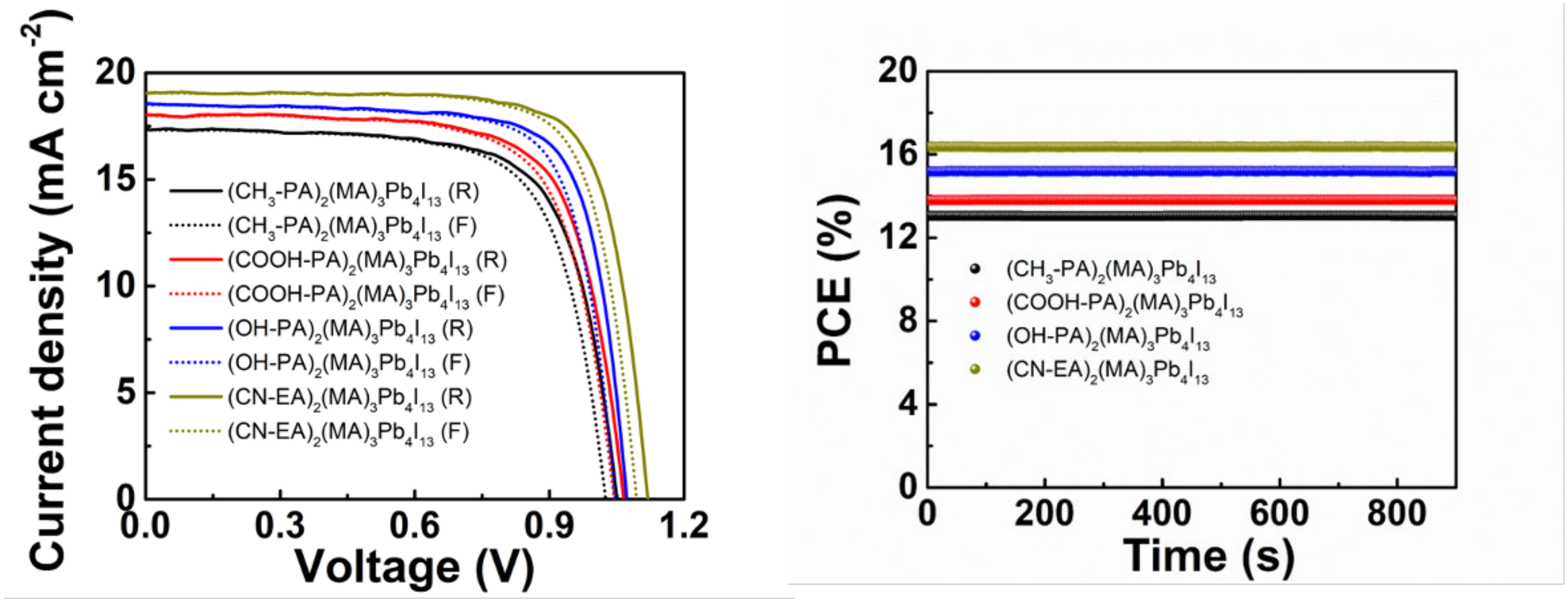

Supplementary Figure 31. (Left) *J-V* characteristics under forward (F) or reverse (R) voltage scan direction and (right) stabilized PCEs of solar cells using $n = 4$ 2D perovskite thin films.

page 18

Princeton University

Perovskite 1-J Cell

Device ID: XZ3-1	Device temperature: 24.7 ± 0.3 °C
5:24 PM 10/1/2019	Device area: 0.119 cm^2 ± 0.6%
Spectrum: ASTM G173 global	Irradiance: 1000.0 W/m^2

NREL
NATIONAL RENEWABLE ENERGY LABORATORY
OSMSS IV System
PV Performance Characterization Team

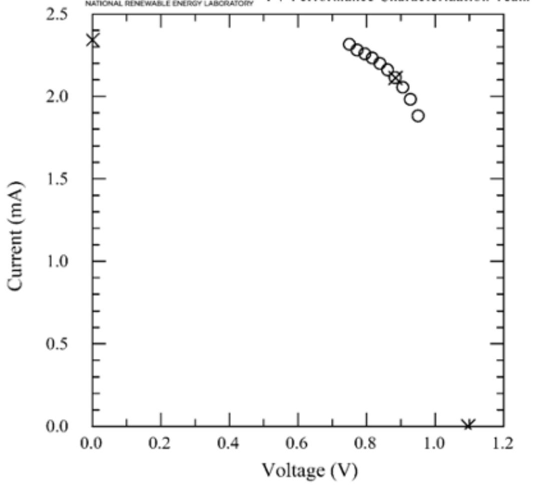

V_{oc} = 1.096 V ± 0.33%	I_{max} = 2.112 mA ± 0.59%
I_{sc} = 2.349 mA ± 0.57%	V_{max} = 0.884 V ± 0.63%
J_{sc} = 19.754 mA/cm^2 ± 1.25%	P_{max} = 1.867 mW ± 0.79%
Fill Factor = (72.5 ± 0.79)%	Efficiency = (15.70 ± 0.22)%

Asymptotic P_{max} scan
Elapsed time:1170.2s.

Supplementary Figure 32. Certified *J-V* characteristics of an $(CN\text{-}EA)_2(MA)_3Pb_4I_{13}$ PSC. Parameter deviations are from instrumental and temperature variations.

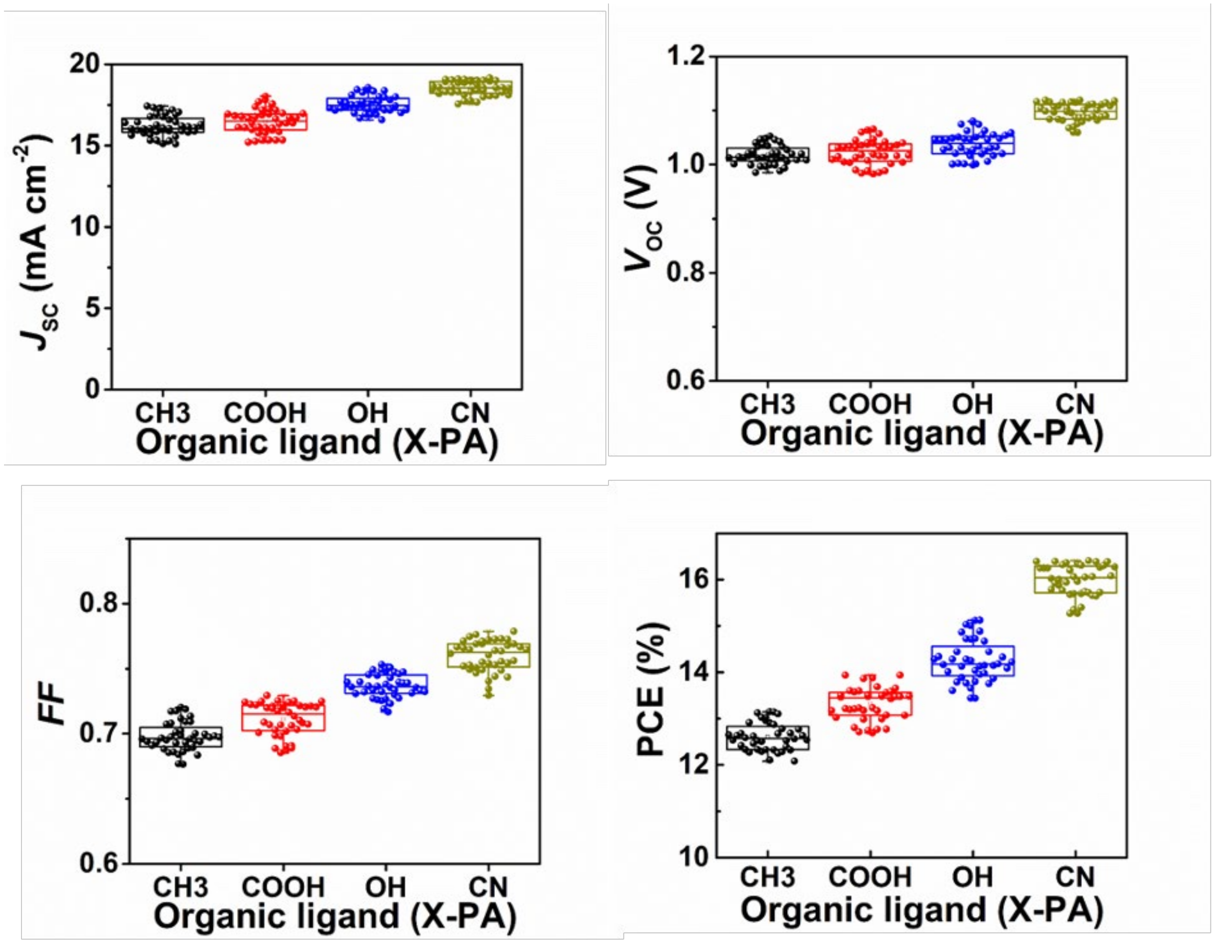

Supplementary Figure 33. Solar-cell performance statistics of PSCs with $n = 4$ 2D perovskite thin films. Values were extracted from testing 40 devices at each condition. Note: CN represents the organic ligand CN-EA.

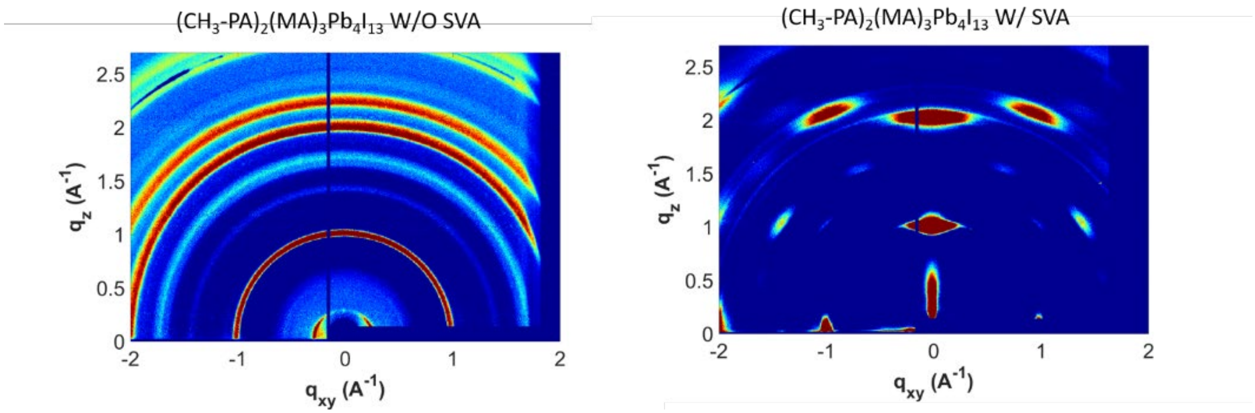

Supplementary Figure 34. GIWAXS patterns of $(PA\text{-}CH_3)_2(MA)_3Pb_4I_{13}$ without and with SVA treatment

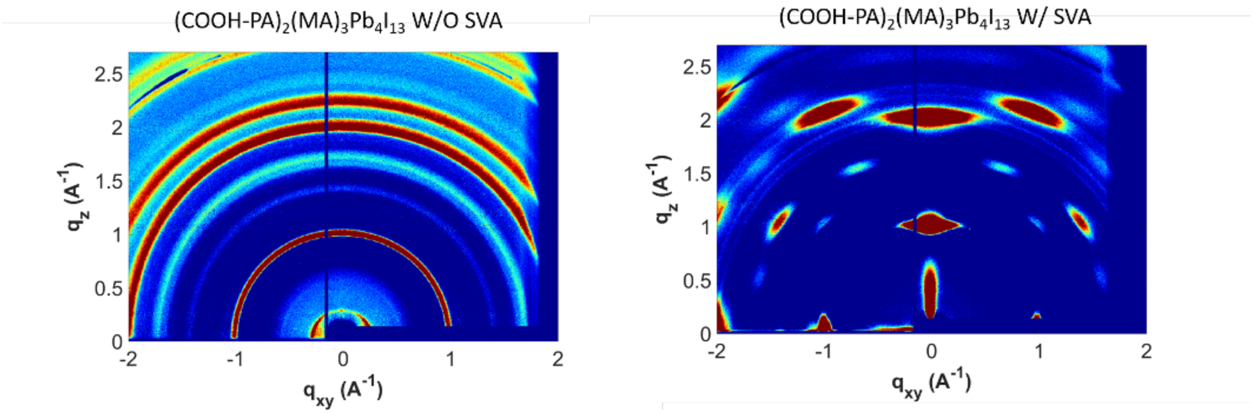

Supplementary Figure 35. GIWAXS patterns of $(PA\text{-}COOH)_2(MA)_3Pb_4I_{13}$ without and with SVA treatment.

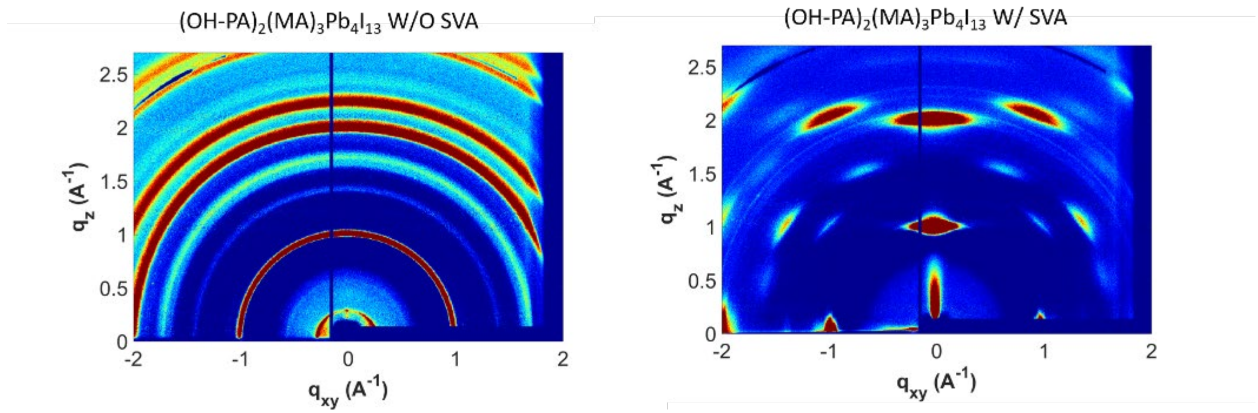

Supplementary Figure 36. GIWAXS patterns of $(PA\text{-}OH)_2(MA)_3Pb_4I_{13}$ without and with SVA treatment.

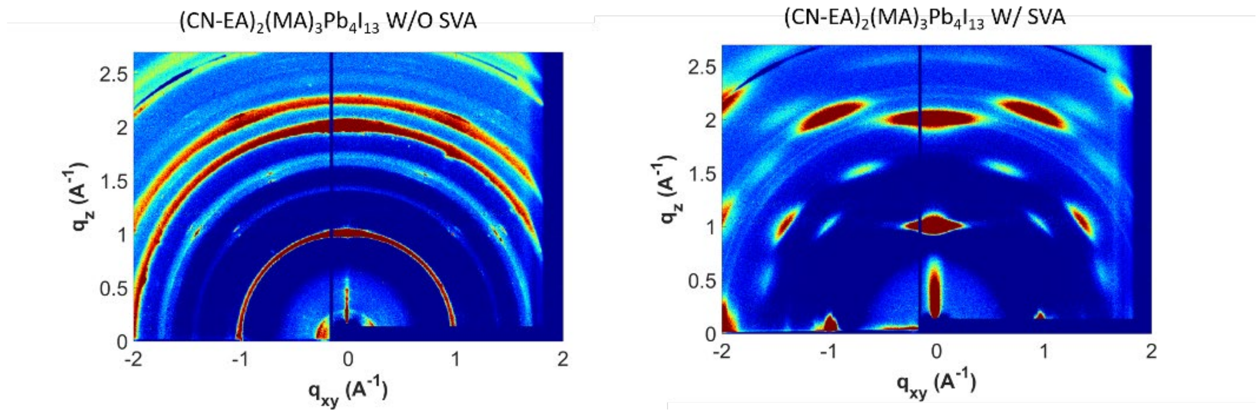

Supplementary Figure 37. GIWAXS patterns of $(CN\text{-}EA)_2(MA)_3Pb_4I_{13}$ without and with SVA treatment.

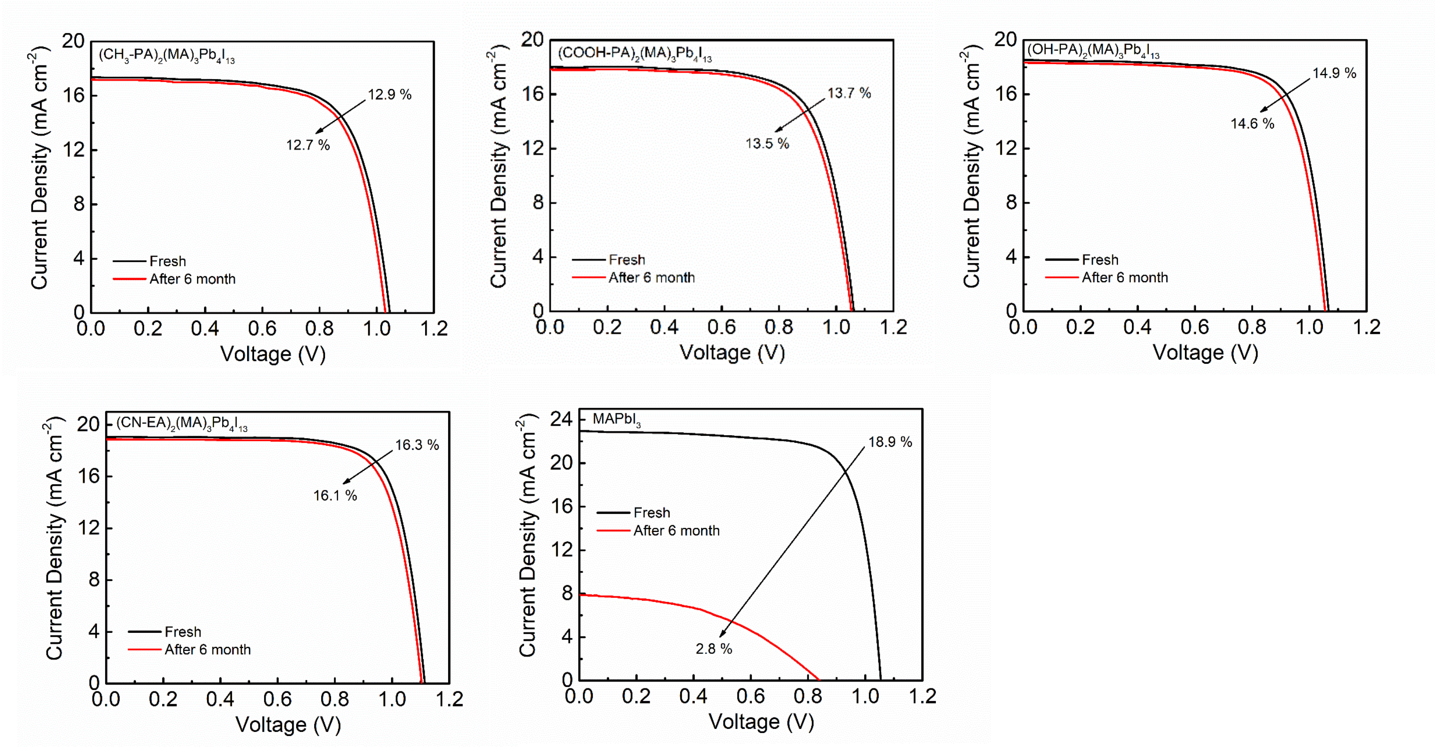

Supplementary Figure 38. J-V characteristics of fresh and 6-month aged un-encapsulated 2D perovskite and MAPbI3 reference solar cells in the dark at 25℃ in 40-60% R.H. in air.

Supplementary Tables

Supplementary Table 1. Comparison between calculated and experimental .CIF files

	CSD (Å)	Pb-I-Pb (°)		Pb-I interplane distance (Å)		Band Gap (eV)	
	**Theory**	**Theory**	**Exp.**	**Theory**	**Exp.**	**Theory**	**Exp.**
**$(CH_3\text{-PA})_2PbI_4$**	1.01	144	149	3.23	3.17	2.26	2.24
**$(COOH\text{-PA})_2PbI_4$**	2.12	153	159	3.20	3.15	2.13	2.21
**$(OH\text{-PA})_2PbI_4$**	2.82	160	164	3.19	3.16	2.01	2.10
**$(CN\text{-EA})_2PbI_4$**	4.25	174	176	3.20	3.18	1.93	1.98

Supplementary Table 2. Lengths and d-spacing of ligands studied from experimental structural files

	d-spacing (Å)	**Length of ligand (Å)**
CH3-PA	13.12	4.91
COOH-PA	12.13	6.13
OH-PA	10.62	4.29
CN-EA	10.17	3.97
PEA	16.07	5.03
BDA	10.10	5.67
NAPH	22.95	8.24

Supplementary Table 3. N-H bond lengths in theoretical .cif file

	**N-H Bond Length from Theoretical CIF file (Å)**			
	**$(CH_3\text{-PA})_2PbI_4$**	**$(COOH\text{-PA})_2PbI_4$**	**$(OH\text{-PA})_2PbI_4$**	**$(CN\text{-EA})_2PbI_4$**
**$N\text{-}H_{L/Equatorial}$**	1.05	1.04	1.06	1.06
**$N\text{-}H_{axial}$**	1.05	1.05	1.04	1.05
**$N\text{-}H_{axial}$**	1.04	1.04	1.04	1.04

For $(OH\text{-PA})_2PbI_4$ and $(CN\text{-EA})_2PbI_4$, the longest N-H bond length is with the proton-like hydrogen that makes a lateral intermolecular interaction with the adjacent ligand.

Supplementary Table 4 Pb-I-Pb Tilt vs Relevant Distance (Å)

	Theory	Experimental
**CSD**	**0.9812**	**0.9601**
Nitrogen-to-Axial Iodides	0.8506	0.8467
Nitrogen-to-Equatorial Iodides	0.9482	0.8042
Hydrogen-to-Axial Iodides	0.5658	NA
Hydrogen-to-Equatorial Iodides	0.9322	NA
Interlayer Spacing	0.1981	0.1783

Supplementary Table 5 Band Gap* vs Relevant Distance (Å)

	Theory	Experimental
**CSD**	**0.9507**	**0.8385**
Nitrogen-to-Axial Iodides	0.7401	0.8054
Nitrogen-to-Equatorial Iodides	0.8394	0.7069
Hydrogen-to-Axial Iodides	0.6001	NA
Hydrogen-to-Equatorial Iodides	0.7721	NA
Interlayer Spacing	0.2892	0.1479

*The band gap for PEA and BDA were taken from the literature.[1,2]

Supplementary Table 6. Summary of structural properties, band gap and mobility of 2D perovskite single crystals.

Material	CSD (Å)	Pb-I-Pb[a] (°)	λ_{onset} (nm)	E_g[b] (eV)	E_{VB}[c] (eV)	E_{CB}[d] (eV)	$\mu_{h,in\text{-}plane}$[e] ($cm^2\ V^{-1}\ s^{-1}$)	$\mu_{h,out\text{-}of\text{-}plane}$[f] ($cm^2\ V^{-1}\ s^{-1}$)
$(CH_3\text{-}PA)_2PbI_4$	1.01	145/149	554	2.24	-5.56	-3.32	0.15 ± 0.04	0.003 ± 0.0012
$(COOH\text{-}PA)_2PbI_4$	2.12	153/159	561	2.21	-5.54	-3.33	0.39 ± 0.08	0.005 ± 0.0011
$(OH\text{-}PA)_2PbI_4$	2.82	160/164	590	2.10	-5.49	-3.39	0.78 ± 0.18	0.006 ± 0.0010
$(CN\text{-}EA)_2PbI_4$	4.25	174/176	626	1.98	-5.43	-3.45	2.4 ± 0.43	0.004 ± 0.0012

[a] Theoretical/Experimental Pb-I-Pb angle. Average angle used for $(CN\text{-}EA)_2PbI_4$ due to two distinct angles in the structural files. [b] Calculated from the equation, $E_g = (1240/\lambda_{onset})$. [c] Determined from the UPS spectra. [d] Determined from the equation, $E_{CB} = E_{VB} + E_g$. [e] In-plane hole mobility. [f] Out-of-plane hole mobility. Mobilities were calculated using Child's law, $J = 9\varepsilon_0\varepsilon_T\mu_h V^2/8L^3$, where J is the current density, L is the active thickness of the 2D perovskite single crystals, μ_h is the hole mobility, ε_T is the relative dielectric constant of the transport medium, ε_0 is the permittivity of free space (8.85×10^{-12} F m^{-1}), V is the applied voltage to the device (the configuration of hole-only device is Au/perovskite/Au). Average values were obtained on 16 devices fabricated and tested under the same conditions. We find the in-plane mobility correlates well with CSD, and increases over an order of magnitude by introducing lateral supramolecular interactions.

Supplementary Table 7. Summary of bandgaps and hole mobility of n =1 and n =4 2D perovskites studied in this work. Properties for n=1 2D perovskites were obtained from their single crystals while those for n = 4 2D perovskites were achieved from their thin films. The table shows that both the n=1 and n=4 trends are analogous.

Perovskite	Bandgap (eV)	$\mu_{h,in\text{-}plane}$ (cm^2 V^{-1} s^{-1})	$\mu_{h,out\text{-}of\text{-}plane}$ (cm^2 V^{-1} s^{-1})
$(CH_3\text{-}PA)_2PbI_4$	2.24	0.15 ± 0.04	0.003 ± 0.0012
$(COOH\text{-}PA)_2PbI_4$	2.21	0.39 ± 0.08	0.005 ± 0.0011
$(OH\text{-}PA)_2PbI_4$	2.10	0.78 ± 0.18	0.006 ± 0.0010
$(CN\text{-}EA)_2PbI_4$	1.98	2.4 ± 0.43	0.004 ± 0.0012
$(CH_3\text{-}PA)_2(MA)_3Pb_4I_{13}$	1.71	2.9 ± 0.39	0.38 ± 0.08
$(COOH\text{-}PA)_2(MA)_3Pb_4I_{13}$	1.70	4.0 ± 0.48	0.45 ± 0.11
$(OH\text{-}PA)_2(MA)_3Pb_4I_{13}$	1.67	5.2 ± 0.99	0.50 ± 0.12
$(CN\text{-}EA)_2(MA)_3Pb_4I_{13}$	1.64	7.8 ± 1.19	0.43 ± 0.10

Supplementary Table 8. Summary of the champion and average photovoltaic parameters of 2D perovskite solar cells under reverse voltage scan. Parameters followed by * are champion device parameters. Parameters in bracket are average parameters. Average parameters are calculated based on 40 devices in each condition.

Active layer	J_{SC} (mA cm^{-2})	J_{SC} by EQE (mA cm^{-2})	V_{OC} (V)	FF	PCE (%)
$(CH_3\text{-}PA)_2PbI_4$	3.00*<br>(2.79 ± 0.11)	2.85*	0.80*<br>(0.76 ± 0.03)	0.34*<br>(0.32 ± 0.01)	0.82*<br>(0.57 ± 0.15)
$(COOH\text{-}PA)_2PbI_4$	3.22*<br>(2.97 ± 0.13)	3.05*	0.86*<br>(0.80 ± 0.04)	0.47*<br>(0.45 ± 0.02)	1.30*<br>(1.09 ± 0.13)
$(OH\text{-}PA)_2PbI_4$	3.57*<br>(3.41 ± 0.10)	3.41*	1.00*<br>(0.92 ± 0.04)	0.59*<br>(0.57 ± 0.01)	2.11*<br>(1.87 ± 0.13)
$(CN\text{-}EA)_2PbI_4$	4.12*<br>(4.00 ± 0.10)	4.00*	1.07*<br>(1.04 ± 0.03)	0.71*<br>(0.70 ± 0.01)	3.13*<br>(2.97 ± 0.13)
$(CH_3\text{-}PA)_2(MA)_3Pb_4I_{13}$	17.3*<br>(16.2 ± 0.6)	15.9*	1.05*<br>(1.02 ± 0.02)	0.72*<br>(0.70 ± 0.01)	13.1*<br>(12.6 ± 0.3)
$(COOH\text{-}PA)_2(MA)_3Pb_4I_{13}$	18.0*<br>(16.5 ± 0.7)	16.6*	1.06*<br>(1.03 ± 0.02)	0.73*<br>(0.71 ± 0.01)	13.9*<br>(13.3 ± 0.3)
$(OH\text{-}PA)_2(MA)_3Pb_4I_{13}$	18.6*<br>(17.5 ± 0.5)	17.4*	1.08*<br>(1.05 ± 0.02)	0.75*<br>(0.74 ± 0.01)	15.1*<br>(14.2 ± 0.5)
$(CN\text{-}EA)_2(MA)_3Pb_4I_{13}$	19.1*<br>(18.5 ± 0.4)	17.9*	1.11*<br>(1.10 ± 0.02)	0.77*<br>(0.76 ± 0.01)	16.4*<br>(16.0 ± 0.3)

Supplementary Methods

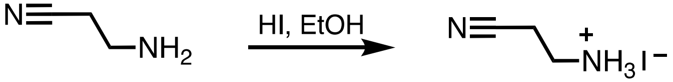

Procedure: 3-amino-propanenitrile (2.00 g, 28.6 mmol, 1.00 equiv.) and EtOH (5.00 ml) was added to a round bottom flask and cooled to 0°C. Hydroiodic acid (47% weight, 9.26 g, 6.18 ml, 1.20 equiv.) was added dropwise and the reaction was allowed to react overnight while warming to room temperature. The reaction was dried under vacuum and the solids were washed extensively with diethyl ether. Upon evaporation, a fine white powder was isolated (2.138 g, 38%). ^{1}H NMR (500 MHz, 298K, DMSO) δ 7.96 (br s), 3.12 (t, J = 7.0 Hz, 2H), 2.83 (t, J = 6.9 Hz, 2H). ^{13}C (125 MHz, 298K, DMSO) δ 118.33, 35.54, 15.29. (ESI+/QTOF): m/z predicted for protonated amine (M = amine) is 70.05310 m/z and found 71.06094 m/z, [M+H]+. A previous manuscript reported the perovskite structure using this ligand;[3] we provide our synthesis and characterization for completion.

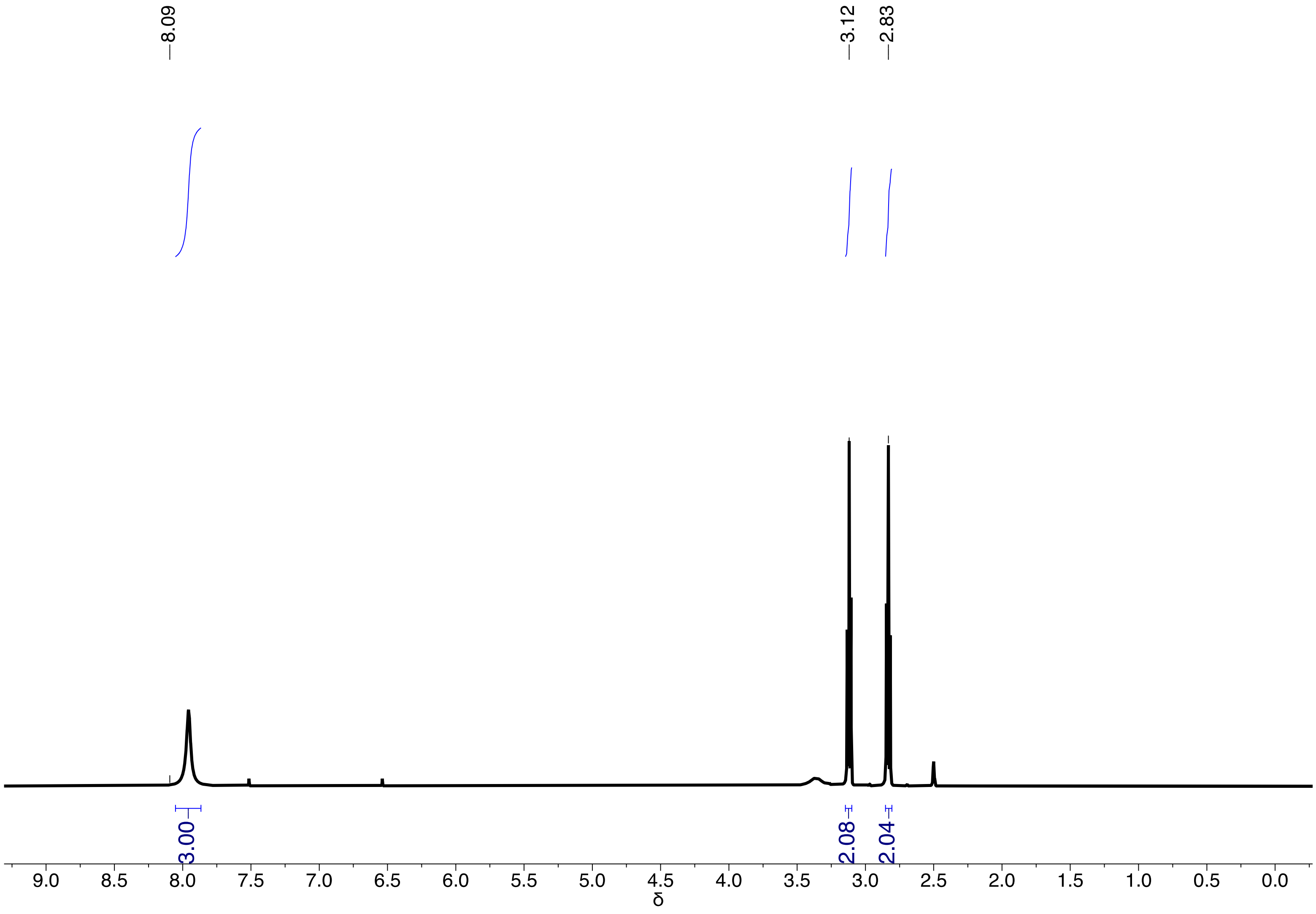

8.09
3.12
2.83
3.00
2.08
2.04
9.0
8.5
8.0
7.5
7.0
6.5
6.0
5.5
5.0
4.5
4.0
3.5
3.0
2.5
2.0
1.5
1.0
0.5
0.0
δ

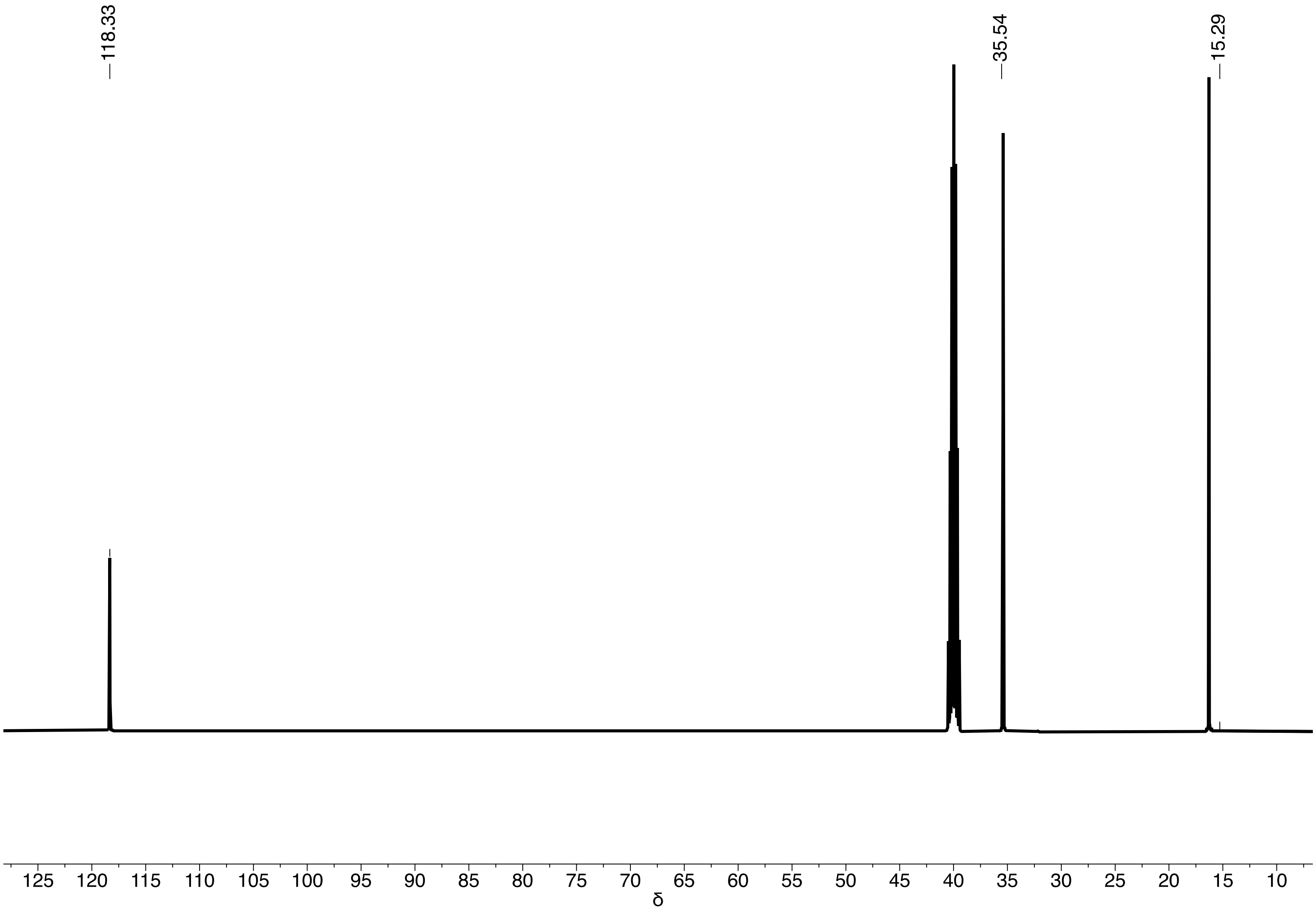
118.33
35.54
15.29
125 120 115 110 105 100 95 90 85 80 75 70 65 60 55 50 45 40 35 30 25 20 15 10
δ

Supplementary Note 1

Some details of our DFT calculations:

The sampling of the k-space (for the vc-relax calculations) is obviously dependent on the case-specific choice of simulation cells and lattice parameters, for example:

For CN-EA:

```
CELL_PARAMETERS (angstrom)
   8.598770996  -0.000006764   0.000003732
   0.000015038  20.074186782  -0.000015524
   0.000004246  -0.000007214   9.863242626

K_POINTS automatic
8 1 8 0 0 0
```

For CH3-PA:

```
CELL_PARAMETERS (angstrom)
   8.395041773  -0.000002111   0.000000000
  -0.000002270   9.025221531   0.000000000
   0.000000000   0.000000000  26.491380350

K_POINTS automatic
8 8 1 0 0 0
```

For COOH-PA:

```
CELL_PARAMETERS (angstrom)
   8.788279236   0.000000577   0.000000000
```

```
  0.000000312   9.177379002   0.000000000
  0.000000000   0.000000000  24.892274581
K_POINTS automatic
8 8 1 0 0 0
```

For OH-PA:

```
CELL_PARAMETERS (angstrom)
  10.659938264   0.000013657  -0.123907549
   0.000011531   9.174561837  -0.000002945
  -0.598238383  -0.000003782   9.215897087

K_POINTS automatic
2 8 8 0 0 0
```

Molecular orbitals in our plane-wave calculations:

In Supplementary Figure 8-10, which contain the molecular orbitals (MOs) of the ligand systems, we derive such MOs using post-processing options in Quantum Espresso and plotting contribution of selected wavefunction(s) to the (pseudo-)charge density. For these calculations we perform "molecule-in-the-box" calculations, in which the size of the box is chosen to be (a relatively large value) 15 Å in each direction. Since the ligands in these cases is a cation or a dimer of cations, a jellium uniform countercharge background is added to the simulation cell to retain the cell neutrality. This is a common practice for simulation of cationic or anionic systems in periodic plane-wave-based DFT calculations. Since for such calculations we only sample the k-space at the gamma point, the bands (in the periodic calculation) can well-represent the MOs of the modeled molecular systems. We then plot each wave-function corresponding to different bands and analyze their relative spacings and shapes. It should be noted that for these figures the molecular structures are fully-relaxed in the box. Thus, the dimer structure is slightly different from when it is embedded in the perovskite background, we will discuss this in the context of Supplementary Figure 11 and 12.

Exploring different conformations of the organic ligands:

In Supplementary Figure 4, we try to explore different local minima for the organic ligands. In addition to exploring the (relaxed) structures that closely mimic the conformation of the ligand once embedded in the perovskite (top row), we deform and re-relax the ligands to obtain other local minima (conformations) for the organic ligands. Since the ligands in these cases is a cation, a jellium uniform countercharge background is added to the simulation cell to retain the cell neutrality.

Electron density differences (EDD) plots:

To generate the plots in Supplementary Figure 5, three charge densities ($\rho(\vec{r})$) are calculated and the quantity ($\rho_{with-ligand}(\vec{r}) - \rho_{without-ligand}(\vec{r})$- $\rho_{only-ligand}(\vec{r})$)) is calculated. Here, the first term is the vc-relaxed structure of the perovskite, the structures for the second and the third term are frozen at this initial vc-relaxed structure. For the second term, the specific ligand (that we are interested to understand its bonding to the environment) is removed; while for the third term, the simulation cell only has that specific ligand. Since a) the ligand is cationic, b) the system without the ligand is anionic, and c) we are required to have charge neutral simulation cells under periodic boundary conditions (we use a plane-wave-based DFT code), we introduce a homogeneous positive background charge (+1 *e*) for the second term, while we introduce a negative background charge with the same magnitude for the third term to keep the chemically relevant charge-balance for every term of this quantity. Such homogeneous charge background is known in the literature as a jelium model. The purpose of such EDD plots is to understand how charge density rearranges itself upon "turning-on" the ligand's interaction with its environment. Such quantity has been previously shown to provide valuable information about bonding phenomena in both inorganic, and hybrid organic-inorganic systems.[4,5]

For Figures 2a and 2b, the EDD is defined as the charge density of the two isolated monomers (frozen at their dimer structure) subtracted from the charge density of the dimer. We also introduce the appropriate charge background to keep the overall charge of the simulation cell equal to zero, as explained above. Thus, the calculation with a (cationic) monomer has a -1*e* background charge, while that with the dimer has a -2*e* background charge per simulation cell. In Figure 2c, the EDD is calculated as $\rho_{system} - \rho_{system/no-dimer} - \rho_{dimer}$ and shows the electron density redistribution upon embedding the dimer in the perovskite. The reason why this EDD plot is calculated in this fashion is to capture the interactions between dimer and the perovskite background rather than internal monomer-monomer interactions. Here, the (anionic) system without dimer has a +2*e* neutralizing background charge while the (cationic) dimer has a -2 *e* neutralizing background charge.

Characteristics of CBM and VBM at our hybrid organic-inorganic systems:

To understand the character of the CBM and VBM in our studied systems we plotted the contribution of selected wavefunctions to the (pseudo-)charge density for the bands that constitute the very bottom of the CBM and the very top of VBM, (in Supplementary Figure 16) we call these LUMO and HOMO, respectively. Supplementary Figure 17 depicts the PDOS for our studied systems.

On the electrostatic versus covalent nature of ligand-ligand bonds:

In the context of lone-pair bonding between N and O lone-pairs (NH_3, H_2O, amines, alcohols, ethers) and a wide range of inorganic surfaces, it has been shown that the electrostatics is the determining factor in bonding between the lone-pair (Lewis base) and the surface Lewis acid sites.[5] This concept has been generalized for the hybrid organic-inorganic systems.[6] The electrostatic nature of the lone-pair bonding means that the hybridization between orbitals is not the main driving force for the bond formation, rather it is electrostatic interaction between the lone-pair and the electrostatic field of the Lewis acid site (enhanced by lone-pair polarization toward the acid site) that drives forth the bonding. Recently, this picture has also been confirmed using maximally localized Wannier functions (MLWFs) analyses.[7] In this work, our calculations also paint a similar picture. As per Fig. 2(a), upon the dimer formation, the electron count on cyano group's nitrogen only decreases by a small amount (0.03), thus the picture of a covalently driven bond in which there is significant charge transfer from the Lewis base to Lewis acid site does not hold. As mentioned in the context of Fig. 2b, the bonding is driven by charge displacement around the dimer ring so that the CN group's electrons can effectively get closer to the electrostatic potential well (ESPW) made by the proton-like hydrogens. This concept is similar to the lone-pair polarization phenomenon discussed in the context of lone pair-surface bonds. Also similar to the surface-bonding case, the Lewis acid site (here proton-like hydrogen) gets elongated and acquires more positive charge to create a deeper ESPW. This is known as adsorbate-induced lifting in the context of inorganic surface chemistry.[5] In order to get a better idea about the charge transfer, from cyano group's nitrogen to the proton-like hydrogen (of the other ligand) it binds to, we have overlaid the Bader volume of the protonlike hydrogen with the EDD plot to see whether there is an electron accumulation (within the boundary of the proton-like hydrogen) between the N and the proton-like hydrogen.

Structural changes in the dimers upon embedding in the perovskite backbone:

In Figure 2 (a) and (b), we showed the bound dimer geometry of two cationic $(CN\text{-}EA)^+$ ligands. In order to focus on the chemistry associated with the dimer formation, as indicated in the main text, those calculations were done with a neutralizing uniform charge background (jellium), in a large supercell (a cube each side 20 Å). This is required for DFT simulations of cationic or anionic systems in DFT codes based on plane-waves and periodic boundary conditions. In order to understand the effect of the perovskite system on the characteristics of the dimer and the bonding between the two systems, we made the EDD plot depicted in Figure 2(c). In this figure one can notice the formation of effective hydrogen bonds between the proton-like hydrogens of the ammonium groups

(not the one bonding to the cyano group) and the axial iodides. Such additional “side” hydrogen bonding as well as the dispersion interactions between the dimer and the perovskite environment change the structure of the dimer. Such changes are quantified and tabulated in Supplementary Figure 8. Some of the important changes include the weakening of the bonding between the ammonia’s proton-like hydrogen and the cyano group (associated with an increased bond length), and a relative widening of the dimer (due to side interactions with axial iodides). The former is due to the re-distribution of the positive charge on the ammonia group: upon formation of additional hydrogen bonds with axial iodides, the positive charge is less concentrated on the original proton-like hydrogen thus the bond with the cyano group relatively weakens.

Bonding between cationic dimers and the neutralizing charge background:

In this work we discussed the formation of organic cationic dimers embedded in the hybrid perovskite structure. The formation of such entities is a direct result of the neutralizing charge background of the inorganic backbone. The anionic inorganic background acts as a “glue” that helps cationic entities with similar charge sign to favorably attach to one another. This is evident in Supplementary Figure 6, where the dimer binding energy is plotted against inverse box size. Here, the simulation cell with periodic boundary conditions (box) has a cubic shape, and the binding energy scales as $\frac{1}{L}$, which is dictated by the Madelung energy. Such energy is associated with the interaction of periodic charge arrays due to the added neutralizing background in our box. Such uniform neutralizing charge background (jellium) is a necessary part of charged-cell calculations with periodic boundary conditions (as is the case in the Quantum Espresso package implemented via plane-waves basis set). As per Supplementary Figure 6, In the limit of an infinite box ($\frac{1}{L} = 0$), i.e., infinitely dilute neutralizing charge background, the binding energy for the cationic (CN-EA) dimer is a positive value. This means without a neutralizing charge background the two monomers do not favor forming a dimer, and the bound-state is merely a local energy minimum. Thus, in order to assign a binding energy to the dimers embedded in the hybrid perovskite structure with the specific array of neutralizing inorganic backbone background, one cannot rely on the molecules-in-box calculations or calculations based on atomic basis sets. Instead we need to design a numerical experiment that can produce a physically-relevant number to the specific charge environment and cationic dimer arrangement in the hybrid perovskite structure.

The numerical experiment we designed to assign a binding energy to the dimer bonds is shown in Supplementary Figure 7. We turn off the van der Waals add-on (Grimme-D3) correction, and calculate the energy of the bare perovskite structure and the broken perovskite structures. With van der Waals interactions absent in the calculations, the energy difference between the bare and broken (scf calculation with monomers displaced by 5 Å along the perpendicular direction to inorganic plane) perovskite structures, is dominated by the penalty for breaking the dimer bonds. We will add the effect of vdW using another numerical experiment: we take the embedded dimer geometry and replace the rest of the perovskite with a uniform neutralizing charge background (jellium), then we calculate the binding energy between the two monomers with and without Grimme-D3 correction. Although one cannot read the

absolute value of the binding energy due to the finite box size (as explained above), the amount of vdW correction can be measured using such calculation, as the box size and charge states are the same with/without the vdW interaction, and such interaction is much less sensitive to the neutralizing charge background than the total energy of the cell used to calculate the overall binding energy. The amount of vdW interaction is then added to the value calculated using the numerical experiment in Supplementary Figure 7. For, CN-EA and OH-PA dimers the contribution of non-vdW and vdW interactions to the binding energy of the dimer (per monomer) is calculated as -0.45 and -0.05 eV (for CN-EA), and -0.36 and -0.1eV (for OH-PA), respectively. Thus, based on our numerical experiments, the overall bond energy (per monomer) is estimated to be -0.50 eV (for CN-EA) and -0.45 eV (for OH-PA).

Dimer interactions as a driving force to tune CSD:

In this work, we have performed several types of calculations across the range of our different ligands which point to the importance of lateral interactions in tuning the CSD and thus the structural and electronic properties of 2D hybrid perovskites. The lateral interactions which are caused by the bifunctional nature of our ligands are an effective tuning knob for the CSD. We have defined the CSD to reflect the distance between center of charge of the cationic ligand as measured relative to the inorganic plane. The secondary interactions caused by the bifunctionality of these ligands effectively increase the CSD and decrease the in-plane distortions in the 2D perovskite structure.

The intuition behind the correlation above can be understood based on couple of molecular-level basic chemistry considerations. First, we note that the nature of such secondary interactions includes some sort of lone pair-positive center interaction between the oxygen or nitrogen lone pair and cationic proton-like hydrogens of the ammonium groups. The nature of such interactions is the (oxygen or nitrogen) lone-pair polarization toward the positive center of one of the proton-like hydrogens of the ammonium group (cite: https://doi.org/10.1063/1.5087290). This lone-pair polarization phenomenon can be basically thought of as a combination of ionic and covalent effects in binding between the O- or N- lone pair and a proton-like hydrogen. As the oxygen lone-pair has negative charge and the ammonium group of the ligands is encaged in the negative environment of I^- anions in the inorganic plane, the polarization of the lone-pair toward the hydrogen group can energetically benefit from the withdrawal of the ammonium group away from the negatively charged iodine cage in the inorganic plane. Such withdrawal decreases the interaction between the cationic ammonium groups and the equatorial anionic iodines, thus decreases the in-plane distortion in the 2D perovskite structures (increases mobility).

Such withdrawal of the NH_3 head toward the organic bilayer can also be rationalized based on the notions of the effective medium theory (EMT) (please cite: https://doi.org/10.1103/PhysRevB.21.2131). Here, the proton-like hydrogens of the ammonium group need to find optimum electron charge density in their immediate environment. Initially, without secondary interactions, such optimum electron density is mostly provided by the equatorial iodine anions of the inorganic plane, but upon the creation of secondary lateral interactions part of the optimum charge density can be provided by the polarized nitrogen or oxygen lone-pair, thus the ammonium group can increase its distance (decrease its orbital overlap) with the inorganic plane. The counterpart

phenomenon in the field of inorganic surface chemistry is known as adsorbate-induced cation lifting (cite: https://doi.org/10.1021/acs.inorgchem.8b00902 & https://www.nature.com/articles/ncomms12888).

In this work we provide a set of calculations which shows that the extent of such lateral interactions (dominated by lone pair-positive center interactions) controls the CSD. For example, in the context of Supplementary Figure 7, we compared CN-EA and OH-PA dimers and showed that the contribution of non-vdW interactions to the binding energy of the dimer (per monomer) is -0.45 eV (for CN-EA), and -0.36 eV (for OH-PA), respectively; which shows that the CSD is a function of the lateral point interactions in the dimer. Also, the EDD plots of the Supplementary Figure 5 across perovskites with different ligands, show that the greater the interaction between the two ligands (forming dimers), the less the interaction with the equatorial iodines is. Yet another measure for the strength of the lateral interactions (here dominated by lone pair-proton-like hydrogen interaction) is the effective positive Bader charge on the proton-like hydrogens of the ammonium group. Such positive charge drives the attraction between the N- or O- lone pair and the ammonium group. Such total Bader charge (for proton-like hydrogens of the ammonium group) is calculated to be 1.36, 1.37, 1.42, and 1.44 $+e$ for the ligands with $-CH_3$, -COOH, -OH, and -CN, respectively. We can see that such a charge is also correlated with CSD value, which is another indication that the strength of the lateral ligand interactions controls the CSD and thus the structural and electronic properties of this class of 2D hybrid perovskites.

In addition to the chemical arguments above discussing the nature and strength of chemical bonds as a responsible agent to tune the CSD, we also note that the correlation between the CSD and lateral interactions (leading to the formation of molecular dimers or zippers) can also be partially rationalized using steric hinderance arguments. In the case of ligands with a CH_3 tail and absence of significant lateral interactions between the organic plane ligands, the NH_3 head has full geometric freedom and can well penetrate into the iodines cage at the top of the inorganic plane and interact with and distort the equatorial iodines. This is the case as there is no additional constraint stemming from interactions between this NH_3 head and a bifunctional tail of another ligand. In contrast, introducing the lateral interactions and formation of favorable molecular dimers and zippers creates additional constrains for interaction between the NH_3 head and the equatorial iodines in the inorganic planes. For example, the effective size of a dimer or molecular zipper is much bigger than a single ligand, and as a result the NH_3 head cannot fully penetrate the iodine cage, being withdrawn toward the organic bilayer, which increases the CSD and decreases the in-plane lattice distortions in the inorganic plane.

Supplementary References

(1) Liu, Y.; Zhang, Y.; Yang, Z.; Ye, H.; Feng, J.; Xu, Z.; Zhang, X.; Munir, R.; Liu, J.; Zuo, P.; et al. Multi-Inch Single-Crystalline Perovskite Membrane for High-Detectivity Flexible Photosensors. *Nat. Commun.* **2018**, *9* (1), 5302.

(2) Shen, Y.; Liu, Y.; Ye, H.; Zheng, Y.; Wei, Q.; Xia, Y.; Chen, Y.; Zhao, K.; Huang, W.; Liu, S. (Frank). Centimeter-Sized Single Crystal of Two-Dimensional Halide Perovskites Incorporating Straight-Chain Symmetric Diammonium Ion for X-Ray Detection. *Angew. Chemie Int. Ed.* **2020**, *59* (35), 14896–14902.

(3) Mercier, N.; Louvain, N.; Bi, W. Structural Diversity and Retro-Crystal Engineering Analysis of Iodometalate Hybrids.

CrystEngComm **2009**, *11* (5), 720–734.
(4) Kakekhani, A.; Ismail-Beigi, S. Polarization-Driven Catalysis via Ferroelectric Oxide Surfaces. *Phys. Chem. Chem. Phys.* **2016**, *18* (29), 19676–19695.
(5) Kakekhani, A.; Roling, L. T.; Kulkarni, A.; Latimer, A. A.; Abroshan, H.; Schumann, J.; AlJama, H.; Siahrostami, S.; Ismail-Beigi, S.; Abild-Pedersen, F.; et al. Nature of Lone-Pair–Surface Bonds and Their Scaling Relations. *Inorg. Chem.* **2018**, *57* (12), 7222–7238.
(6) Kakekhani, A.; Katti, R. N.; Rappe, A. M. Water in Hybrid Perovskites: Bulk MAPbI3 Degradation via Super-Hydrous State. *APL Mater.* **2019**, *7* (4), 41112.
(7) Fu, Z.; Yang, B.; Wu, R. Understanding the Activity of Single-Atom Catalysis from Frontier Orbitals. *Phys. Rev. Lett.* **2020**, *125* (15), 156001.
(8) Silber, D.; Kowalski, P. M.; Traeger, F.; Buchholz, M.; Bebensee, F.; Meyer, B.; Wöll, C. Adsorbate-Induced Lifting of Substrate Relaxation Is a General Mechanism Governing Titania Surface Chemistry. *Nat. Commun.* **2016**, *7* (1), 12888.
