# Supplementary material for: A charge transfer framework that describes supramolecular interactions governing structure and properties of 2D perovskites
Source: Nat Commun. 2022 Jul 8;13:3970. doi: 10.1038/s41467-022-31567-y (PMC9270412; doi:10.1038/s41467-022-31567-y)
Supplement: Supplementary file 3 — Reporting Summary [file 41467_2022_31567_MOESM3_ESM.pdf]

nature research

Corresponding author(s): Yueh-Lin Loo

Solar Cells Reporting Summary

Nature Research wishes to improve the reproducibility of the work that we publish. This form is intended for publication with all accepted papers reporting the characterization of photovoltaic devices and provides structure for consistency and transparency in reporting. Some list items might not apply to an individual manuscript, but all fields must be completed for clarity.

For further information on Nature Research policies, including our data availability policy, see Authors & Referees.

• Experimental design

Please check: are the following details reported in the manuscript?

1. Dimensions

Area of the tested solar cells — [x] Yes [ ] No — 0.12 cm2, confirmed by NREL (Supplementary Figure 32)

Method used to determine the device area — [x] Yes [ ] No — Methods

2. Current-voltage characterization

Current density-voltage (J-V) plots in both forward and backward direction — [x] Yes [ ] No — Supplementary Figure 27 and Supplementary Figure 31

Voltage scan conditions
For instance: scan direction, speed, dwell times — [x] Yes [ ] No — Methods

Test environment
For instance: characterization temperature, in air or in glove box — [x] Yes [ ] No — Methods

Protocol for preconditioning of the device before its characterization — [x] Yes [ ] No — Methods

Stability of the J-V characteristic
Verified with time evolution of the maximum power point or with the photocurrent at maximum power point; see ref. 7 for details. — [x] Yes [ ] No — Supplementary Figure 27 and Supplementary Figure 31

3. Hysteresis or any other unusual behaviour

Description of the unusual behaviour observed during the characterization — [x] Yes [ ] No — Supplementary Figure 27 and Supplementary Figure 31

Related experimental data — [x] Yes [ ] No — Supplementary Figure 27 and Supplementary Figure 31

4. Efficiency

External quantum efficiency (EQE) or incident photons to current efficiency (IPCE) — [x] Yes [ ] No — Figure 4b

A comparison between the integrated response under the standard reference spectrum and the response measure under the simulator — [x] Yes [ ] No — Supplementary Table 8

For tandem solar cells, the bias illumination and bias voltage used for each subcell — [ ] Yes [x] No — Not relevant

5. Calibration

Light source and reference cell or sensor used for the characterization — [x] Yes [ ] No — Methods

Confirmation that the reference cell was calibrated and certified — [x] Yes [ ] No — Methods

Calculation of spectral mismatch between the reference cell and the devices under test

- [x] Yes — Methods
- [ ] No

6. Mask/aperture

Size of the mask/aperture used during testing

- [x] Yes — Methods
- [ ] No

Variation of the measured short-circuit current density with the mask/aperture area

- [x] Yes — Methods
- [ ] No

7. Performance certification

Identity of the independent certification laboratory that confirmed the photovoltaic performance

- [x] Yes — National Renewable Energy Laboratory, Department of Energy, US
- [ ] No

A copy of any certificate(s)
Provide in Supplementary Information

- [x] Yes — Supplementary Figure 32
- [ ] No

8. Statistics

Number of solar cells tested

- [x] Yes — Supplementary Figure 27 and Supplementary Figure 33
- [ ] No

Statistical analysis of the device performance

- [x] Yes — Supplementary Figure 27 and Supplementary Figure 33
- [ ] No

9. Long-term stability analysis

Type of analysis, bias conditions and environmental conditions
For instance: illumination type, temperature, atmosphere humidity, encapsulation method, preconditioning temperature

- [x] Yes — Methods
- [ ] No
