# Supplementary material for: A charge transfer framework that describes supramolecular interactions governing structure and properties of 2D perovskites
Source: Nat Commun. 2022 Jul 8;13:3970. doi: 10.1038/s41467-022-31567-y (PMC9270412; doi:10.1038/s41467-022-31567-y)
Supplement: Supplementary file 4 — Author Checklist [file 41467_2022_31567_MOESM4_ESM.docx]

| 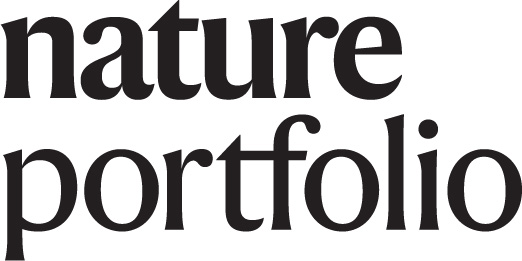 |  |
| --- | --- |

| **Author Checklist** | NCOMMS-22-14447-T |
| --- | --- |
| 0000000000000000000000000000000000000000000000000000000000000 | 0000000000000000000000000000000000000 |
| **Please check the items below carefully and add a response in each row of the table to indicate the changes that you have made. Please also check through any additional marked-up edits we may have provided within the manuscript file.** | |
|  |  |
|  |  |
|  |  |
| Abstract and editor's summary |  |
| Our guidance: | Your response: |
| Your paper will be accompanied by the following editor's summary. Please let us know if there are any inaccuracies: 'Understanding structure-property relationships is important when designing functional materials. Here, authors propose a descriptor to help understand and predict the electronic properties of two-dimensional lead iodide perovskites for photovoltaic applications.' | The editor’s summary is appropriate for the manuscript. |
|  |  |
| Author information |  |
| Our guidance: | Your response: |
|  |  |
| Please review your complete author list to verify that it is complete and accurate. We ask that you consult with your coauthors to ensure that all names, affiliations, and titles are represented correctly. Note that if any authors are added or removed after this point then all authors will be requested to provide approval documentation that could potentially delay the production of your paper. | We confirm that the author list is complete and accurate. |
|  |  |
| Article structure |  |
| Our guidance: | Your response: |
| We can accommodate up to 10 display items (Figures or Tables) in the main article. Each Figure and Table must fit easily within an A4 page (210 x 297 mm). Please ensure that the number and size of your Figures and Tables fulfil these requirements to avoid any delay in the acceptance of your article. | We confirm the number and size of Figures and Tables fulfil these requirements. |
| **To comply with this format and optimise the presentation of data in your Article, we suggest the following changes to the display items in your paper:** |  |
| *When scaled to fit our article template, text and detail in Figures may be too small to be legible. Please provide your final figures at article scale and as a guideline, ensure that text is not smaller than 6 pts. For example, you might want to consider displaying the panels of Figure 4 vertically.* | We have adjusted the layout of Figure 4. |
| **Please ensure your main manuscript file includes the following sections, in this order:** | We have adjusted sections in the order as required.  The section headings of supplementary information have been revised following the instructions.  The labelling of each figure and table have been revised accordingly.  The citations to each supplementary information have been revised |
| *Title Author list Affiliations Abstract Introduction Results Discussion (optional) Results and Discussion (optional) Methods Data Availability Code Availability (if relevant) References Acknowledgements Author Contributions Statement Competing Interests Statement Tables Figure Legends/Captions (for main text figures)* |  |
| We do not edit Supplementary Information files; they will be uploaded with the published article as they are submitted with the final version of your manuscript. Any tracked changes should be removed from the file and the file should be provided as a PDF file. Supplementary Figures do not need to be provided separately.  The only section headings permitted in the Supplementary Information are Supplementary Figures, Supplementary Tables, Supplementary Methods, Supplementary Notes, Supplementary Discussion, Supplementary References. Please note the use of "Supplementary" and that we do not use the "S" prefix.  Please relabel the Supplementary sections accordingly, and make sure to use these labels to cite them in the main text.  Please label each of the main section as “Supplementary Note 1” etc, and Figures and Tables as “Supplementary Figure 1”, “Supplementary Table 1” etc, removing the S prefix. Please also rename the section “References” as “Supplementary References”.  Please replace general citations to the Supplementary Information (e.g. "see Supplementary Information") with specific citations (e.g. "See Supplementary Figure 1", etc.). | . |
|  |  |
|  |  |
| Main text |  |
| Our guidance: | Your response: |
| Presently, there is no clear divide between the introduction and results section. Please consider the introduction should be structured so that all discussion of previous work appears first. The final paragraph should contain only a concise summary of the current work, in the present tense, and begin with a phrase like “In this work” or “Here, we show”. | We have restructured the introduction section and have added the section headings “Introduction” and “Results” to divide the respective sections  We have also structured the summary according to the suggestion. |
| Please consider dividing the Results section into subsections, each with a title of 60 characters or fewer including spaces. | We have divided the Results section into subsections with sub tiles. |
| Please remove or rename the Conclusion heading, as the main text should only include the sections Abstract, Introduction, Results, optional Discussion and optional Methods. We also allow a combined Results and Discussion section. | We have removed the Conclusion heading. |
| Please do not use italics, bold font, underlining or speech marks except in headings unless required for technical terms (in both the main text and the display items). | We have revised the main text and display items accordingly. |
| Please make sure that mathematical terms throughout your manuscript and Supplementary Information (including in figures, figure axes, and legends) conform strictly to the following guidelines. Equations must be supplied in editable format, and not as images. Scalar variables (e.g. x, V, χ) must be typeset in italic, whereas multi-letter variables and functions (e.g. log) must be formatted in roman. Vectors (such as the wavevector k or the magnetic field vector B) must be typeset in bold without italics. | We have revised them accordingly. |
| Please label equations sequentially as (1), (2), (3), etc. | We have labelled the equation accordingly. |
|  |  |
| Figures and Tables |  |
| Our guidance: | Your response: |
| Please see the guidelines linked below for detailed instructions about how your figures should be prepared. Following these instructions will reduce the chances of delays should we need to request replacement artwork from you at a later stage. | All Figures have been amended according to the Guidelines. |
| <https://www.nature.com/documents/NRJs-guide-to-preparing-final-artwork.pdf> |  |
| Chemical line-structures that appear in figures in the manuscript should also be drawn using our ChemDraw stylesheet (linked below) or using the settings from our template — structures can be scaled in proportion to fit our figure dimensions — if you do this though, please only scale atom labels and bond lengths, please do not reduce (or increase) the bond thickness. Also please take note of the chemical structure guide (also linked below). Please try to use consistent ChemDraw settings for all of the structures throughout the manuscript files.  Please note that Extended Data Figures cannot be submitted in .cdx format. If your Extended Data Figures are prepared in ChemDraw, you will need to convert them .jpg, .tif or .eps format for resubmission (.jpg, .tif or .eps file) | All Chemdraw art have been amended according to the Guidelines |
| https://www.nature.com/documents/nr-chemdraw-stylesheet.cds |  |
| https://www.nature.com/documents/nr-chemical-structures-guide.pdf |  |
| For figures that contain ChemDraw structures but are submitted as something other than .cdx files (these are usually figures that have a handful of structures as part of a larger composite figure), please also submit a single .cdx file for each figure that contains all of the ChemDraw structures in that figure. Please supply a separate ChemDraw file for each figure; do not combine all of the ChemDraw structures from all figures into a single file. These additional ChemDraw files should be uploaded to our Manuscript Tracking system as ‘Related Manuscript’ files (not figure files) and labelled clearly. They should not be listed on the Inventory of Supporting Information document. This applies to the following figures: | We have supplied an additional Chemdraw for Figure 1a. |
| **1.a** |  |
| All figure legends must include a brief title that summarises the whole figure. | All figure legends have a brief title. |
| Please make sure that the terms ‘atomic units (a. u.)’ or ‘arbitrary units (arb. units)’ are appropriately used. | We have ensured they are appropriately used. |
| Any abbreviations, symbols or colours present in your figures must be defined in the associated legends. | We have defined them in the associated legends. |
| Chemical structures in figures should be drawn using the Nature Chemistry template or its settings: http://www.nature.com/authors/guides/NR_chemdraw_stylesheet.cds Refer to the Nature Research Chemical Structures Guide for all details: https://www.nature.com/authors/guides/ChemStructureGuide.pdf | We have used the associated template to create the Chemdraws in Figure 1a. |
| In each Figure and Supplementary Figure where error bars are used, they must be defined. | We have defined them in the associated legends. |
| In the legend of Figure 3 please describe the acronym CSD in the first use. | We have addressed this. |
|  |  |
| Data and Code |  |
| Our guidance: | Your response: |
| Nature journals strongly support public availability of data and code. Please deposit the data and code used in your paper into a public data repository, or alternatively, present the data as Supplementary Information. If data can only be shared on request, please explain why in your Data Availability Statement, and also in the correspondence with your editor.   Please note that for some data types, deposition in a public repository is mandatory. Any restrictions on sharing of these data types must be clearly indicated in the statement and discussed with the editor. More information on our data deposition policies and available repositories can be found here: | We have revised the Data Availability Statement as “Data supporting the findings of this work are provided in in the paper and/or the Supplementary Information. The single-crystal X-ray structures data used in this study are available in the Cambridge Crystallographic Data Center (CCDC) database under accession code CCDC 665689 [https://doi.org/[10.5517/ccqbpvk](https://dx.doi.org/10.5517/ccqbpvk)], CCDC 267398 [https://doi.org/[10.5517/cc8z7r6](https://dx.doi.org/10.5517/cc8z7r6)], CCDC 746125 [https://doi.org/[10.5517/cct1dks](https://dx.doi.org/10.5517/cct1dks)], CCDC 705087 [https://doi.org/[10.5517/ccrnprt](https://dx.doi.org/10.5517/ccrnprt)], CCDC 1841681 [https://doi.org/[10.5517/ccdc.csd.cc1ztf29](https://dx.doi.org/10.5517/ccdc.csd.cc1ztf29)], CCDC 1053651 [https://doi.org/[10.5517/cc14cdrn](https://dx.doi.org/10.5517/cc14cdrn)] and CCDC 1840803 [https://doi.org/[10.5517/ccdc.csd.cc1zshr0](https://dx.doi.org/10.5517/ccdc.csd.cc1zshr0)]. Other relevant data can be obtained from the corresponding authors upon request.” |
| <https://www.nature.com/nature-research/editorial-policies/reporting-standards#availability-of-data> |  |
| All published manuscripts reporting original research in Nature Portfolio journals must include a data availability statement, as a separate section before the References and under the heading 'Data Availability'.   The data availability statement must make the conditions of access to the “minimum dataset” that are necessary to interpret, verify and extend the research in the article, transparent to readers.   This minimum dataset may be provided through deposition in public community/discipline-specific repositories, custom proprietary repositories or general repositories like Figshare, Zenodo and Dryad. Providing large datasets in supplementary information is strongly discouraged and the preferred approach is to make data available in repositories. Scientific Data, a Nature Portfolio journal, maintains a list of approved and recommended data repositories to support researchers seeking suitable repositories for their data (https://www.nature.com/sdata/policies/repositories).  The Data Availability Statement should also reference any source data published alongside the paper.  If DOIs are provided, we also strongly encourage including these in the Reference list (authors, title, publisher (repository name), identifier, year).  For clinical datasets or third party data, please ensure that the statement adheres to our policy (https://www.nature.com/nature-research/editorial-policies/reporting-standards#availability-of-data) | We have revised the statement accordingly. Please see above. |
| Please use the following template to provide all the information stated above:  The XX data generated in this study have been deposited in the YY database under accession code ZZ [add hyperlink here]. The XX data are available under restricted access for {insert reason}, access can be obtained by {explain how}. The raw XX data are protected and are not available due to data privacy laws. The processed XX data are available at YY. The XX data generated in this study are provided in the Supplementary Information/Source Data file. The XX data used in this study are available in the YY database under accession code ZZ [Add hyperlink here]. | We have revised the statement accordingly. Please see above. |
| Specific advice on your Data Availability Statement: |  |
| We would prefer that your data be made available in a public repository, but if this is not possible the word "reasonable" should be removed from the Data Availability Statement, which should state that data and code are available on request or the specify the conditions of access. A requirement for publication is that materials, data, code, and associated protocols are made promptly available to readers without undue qualifications (see https://www.nature.com/nature-portfolio/editorial-policies/reporting-standards). | We have revised the statement accordingly. Please see above. |
| An updated solar cells reporting summary must be completed and uploaded as a supplementary information file with the revised manuscript. All points on the reporting summary must be addressed; if needed, please revise your manuscript in response to these points. This checklist is published alongside your manuscript online. Please note that this form is a dynamic "smart pdf" and must therefore be downloaded and completed in Adobe Reader, instead of opening it in a web browser. https://www.nature.com/authors/policies/Photovoltaic.pdf | We have updated the solar cells reporting summary. |
|  |  |
| Methods |  |
| Our guidance: | Your response: |
| Sufficient details of the experiments must be provided in the Methods section such that they could be reproduced without reference to published papers. Use of the term "as described previously" is not encouraged. | We have provided sufficient experimental details in the Method section. |
| All accession codes must be accompanied with their hyperlinks throughout (for example, "5XRN [http://doi.org/10.2210/pdb5XRN/pdb]", "1483958 [https://doi.org/10.5517/ccdc.csd.cc1lt5m6]", "SRP109982 [https://www.ncbi.nlm.nih.gov/sra/?term=SRP109982]", "GSE101099 [https://www.ncbi.nlm.nih.gov/geo/query/acc.cgi?acc=GSE101099]" or "NQLW00000000 [https://www.ncbi.nlm.nih.gov/assembly/GCA_002312845.1/]"). | In the Method section. we have updated the accessions accordingly:  (CH3-PA)_2_PbI_4_: CCDC 665689 [https://doi.org/10.5517/ccqbpvk]  (COOH-PA)_2_PbI_4_: CCDC 267398 [https://doi.org/10.5517/cc8z7r6],  (OH-PA)_2_PbI_4_: CCDC 746125 [https://doi.org/10.5517/cct1dks],  (CN-EA)_2_PbI_4_: CCDC 705087 [https://doi.org/10.5517/ccrnprt]  (PEA)_2_PbI_4_: CCDC 1841681 [https://doi.org/10.5517/ccdc.csd.cc1ztf29],  (BDA)PbI_4_: CCDC 1053651 [https://doi.org/10.5517/cc14cdrn]  (NAPH)_2_PbI_4_: CCDC 1840803 [https://doi.org/10.5517/ccdc.csd.cc1zshr0]. |
|  |  |
| References |  |
| Our guidance: | Your response: |
| All references must be cited in numerical order. The reference list will be formatted according to the Nature style by our journal production team, however please ensure that references contain all of the information required, eg:  Kurumada, S., Takamori, S. & Yamashita, M. An alkyl-substituted aluminium anion with strong basicity and nucleophilicity. <i>Nat. Chem.</i> <b>12</b>, 36–39 (2020). | We have revised all references accordingly. |
| Supplementary References should appear at the end of the Supplementary Information file, and must be self-contained and numbered from 1. References mentioned in both the main text and the Supplementary Information should be part of both reference lists so that the Supplementary Information does not refer to the reference list in the main paper and vice versa. | We have revised Supplementary References accordingly. |
|  |  |
| End matter |  |
| Our guidance: | Your response: |
| Nature Portfolio defines Competing Interest (CI) as financial and non-financial interests (including but not limited to funding, employment, stocks, shares, patents, personal or professional relationships with individuals or institutions, and unpaid membership advocacy) that could be perceived to directly undermine the objectivity, integrity, and value of a publication, or could be seen as having an influence on the judgments and actions of authors with regard to objective data presentation, analysis, and interpretation.  Please thoroughly review our policy on Competing Interests and include a detailed statement both in your final manuscript file and in our manuscript tracking system. Please ensure the statements are identical in both. Be specific about how each point stated relates to the research and list applicable author initials, and/or patent numbers.  If there are no competing interests, a negative statement must be included. |  |
| <https://www.nature.com/nature-research/editorial-policies/competing-interests> | The authors declare no competing interests. |
| Please confirm that all relevant funding awarded to each author is described in the Acknowledgements section. List each grant number, followed by the initials of the author who received it. | We confirm all relevant funding awarded to each author is described in the Acknowledgements section |
|  |  |
| Preparing your manuscript files |  |
| Our guidance: | Your response: |
| Unless otherwise stated please limit individual file sizes to approximately 30MB. We strongly encourage the use of repositories for large datasets or source data due to size considerations. | All individual file sizes are below 30MB. |
| To ensure maximum visibility for your work, we may tweet about your paper following publication. If you would like us to include the Twitter handles of the first author(s), corresponding author(s), lab or institution in this tweet, please provide them in your cover letter. We would also welcome your suggestions for hashtags to use when tweeting about the work. |  |
| Please provide figures as individual vector files with editable text. Acceptable file types for figures are .ai, .eps, .pdf or Chem Draw for fully editable vector-based art. For detailed guidance on figure preparation, see https://www.nature.com/documents/aj-artworkguidelines.pdf | All Figures are supplied as .ai files |
| Please note that all Supplementary Information must be provided as a single separate PDF file, not within the manuscript file.  All Supplementary Information items (e.g. Supplementary Figures, Supplementary Tables, Supplementary Methods, Supplementary Notes, Supplementary Discussion, Supplementary References) must be included in one PDF document. Please refer to our formatting guide when preparing your supplementary information file: https://www.nature.com/documents/ncomms-formatting-instructions.pdf   All Supplementary Information files (e.g. Supplementary Data, Supplementary Software, etc.) must be cited in the main text.  Every Supplementary Figure must be accompanied by a legend of up to 350 words, referring to all panels, and a brief title that summarises the whole figure.  Only Supplementary Movie, Audio, Data and Software files should be submitted separately from the Supplementary Information. | We have provided the Supplementary information in a single PDF file. |
| The use or adaptation of previously published images is strongly discouraged. If this is unavoidable, please request the necessary rights documentation to re-use such material from the relevant copyright holders and return this to us when you submit your revised manuscript. Please check whether your manuscript or Supplementary Information contain third-party images, such as figures from the literature, stock photos, clip art or commercial satellite and map data.  For more information on what constitutes ownership by a third party, please contact our Editorial Assistant at naturecommunications@nature.com | There is no use or adaptation of previously published images. |
|  |  |
|  |  |
| Forms to complete |  |
| Our guidance: | Your response: |
| **Editorial Policy Checklist** |  |
| Please update and upload a final version of the Editorial Policy Checklist with your revised manuscript files. A blank Editorial Policy Checklist can be found via the link below. Note that this form is a dynamic ‘smart pdf’ and must be downloaded and completed in Adobe Reader. | We have updated and uploaded the Editorial Policy Checklist. |
| Please update your current checklist or download from: |  |
| <https://www.nature.com/documents/nr-editorial-policy-checklist.zip> |  |
|  |  |
|  |  |
| **You will need to upload:** |  |
| Editorial Policy Checklist |  |
| Completed Third Party Rights Table (if relevant) |  |
| A completed copy of this checklist |  |
|  |  |
| Separate Figure files |  |
| Inventory of Supporting Information |  |
| A Supplementary Information file |  |
| Reporting Summary |  |
| Solar Cells Reporting Summary |  |
| ChemDraw files |  |
|  |  |
